# Consumer Perspectives for a Future Mobile App to Document Real-World Listening Difficulties: Qualitative Study

**DOI:** 10.2196/47578

**Published:** 2024-07-23

**Authors:** Karyn Galvin, Dani Tomlin, Barbra H B Timmer, Zoe McNeice, Nicole Mount, Kathleen Gray, Camille E Short

**Affiliations:** 1 Department of Audiology and Speech Pathology University of Melbourne Carlton Australia; 2 School of Health and Rehabilitation Sciences University of Queensland Brisbane Australia; 3 Sonova AG Staefa Switzerland; 4 Centre for Digital Transformation of Health University of Melbourne Carlton Australia; 5 Melbourne Centre for Behaviour Change Melbourne School of Psychological Sciences University of Melbourne Carlton Australia; 6 Melbourne School of Health Sciences University of Melbourne Carlton Australia

**Keywords:** adults, hearing loss, listening difficulties, digital health, app, self-management, mobile health, smartphone, mobile phone

## Abstract

**Background:**

By enabling individuals with hearing loss to collect their own hearing data in their personal real-world settings, there is scope to improve clinical care, empower consumers, and support shared clinical decision-making and problem-solving. Clinician support for this approach has been established in a separate study.

**Objective:**

This study aims to explore, for consumers with hearing loss, their (1) experiences of listening difficulties, to identify the data an app could usefully collect; (2) preferences regarding the features of mobile apps in general; and (3) opinions on the potential value and desirable features of a yet-to-be designed app for documenting listening difficulties in real-world settings.

**Methods:**

A total of 3 focus groups involved 27 adults who self-reported hearing loss. Most were fitted with hearing devices. A facilitator used a topic guide to generate discussion, which was video- and audio-recorded. Verbatim transcriptions were analyzed using inductive content analysis.

**Results:**

Consumers supported the concept of a mobile app that would facilitate the documenting of listening difficulties in real-world settings important to the individual. Consumers shared valuable insights about their listening difficulties, which will help determine the data that should be collected through an app designed to document these challenges. This information included early indicators of hearing loss (eg, mishearing, difficulty communicating in groups and on the phone, and speaking overly loudly) and prompts to seek hearing devices (eg, spousal pressure and the advice or example provided by others, and needing to rely on lipreading or to constantly request others to repeat themselves). It also included the well-known factors that influence listening difficulties (eg, reverberation, background noise, group conversations) and the impacts and consequences of their difficulties (eg, negative impacts on relationships and employment, social isolation and withdrawal, and negative emotions). Consumers desired a visual-based app that provided options for how data could be collected and how the user could enter data into an app, and which enabled data sharing with a clinician.

**Conclusions:**

These findings provide directions for the future co-design and piloting of a prototype mobile app to provide data that are useful for increasing self-awareness of listening difficulties and can be shared with a clinician.

## Introduction

Hearing loss affects an individual’s ability to communicate with others, can significantly impact participation in activities and quality of life [1], and may lead to profound social and emotional consequences, including feelings of loneliness, isolation, and frustration [2,3]. The primary treatment for hearing loss is fitting hearing aids. Hearing aids enhance access to sound, including speech signals, potentially leading to increased social activity and improved quality of life [2,4]. Moreover, the use of hearing aids has been shown to reduce listening effort [5] and decrease communication effort for both the hearing aid wearer and their communication partner [6]. At a neurological level, hearing aids have been shown to reverse the cross-modal reorganization of the auditory cortex by vision [7]. Furthermore, a meta-analysis [8] indicated that hearing aid use decreases the risk of long-term cognitive decline and is associated with improved general cognition scores in the short term. Despite the clear and wide-ranging benefits of hearing aids, many individuals choose either not to obtain them or not to use them [9,10].

Lack of self-awareness regarding listening difficulties and their impacts has been identified as a key factor in the low uptake of hearing aids, according to systematic reviews from 2012 [11] and 2023 [12]. One potential intervention to improve self-awareness is a mechanism that helps individuals document their listening difficulties as they occur in real-world settings. Such an intervention could promote help-seeking behavior and the uptake of hearing aids by increasing self-awareness of the social, emotional, and environmental contexts in which listening difficulties occur; the degree of difficulty experienced; and the associated impacts. Furthermore, this intervention could support the ongoing use of hearing aids by facilitating the collection of personally meaningful data that demonstrate change as a result of hearing aid use [13]. Ultimately, this approach could improve care by empowering consumers and supporting shared clinical decision-making and problem-solving. The need for consumer empowerment and support for shared decision-making are relevant at all levels of hearing loss as consumers first seek help, consider being fitted with initial or updated hearing aids, decide whether their current hearing aids provide benefit, and, for those with more significant losses, consider receiving 1 or even 2 implanted hearing devices, such as cochlear implants. Audiological research consistently highlights the need for more patient-centered care of this type [14,15].

Mobile apps are a recognized mechanism for capturing real-world patient data and are used to support self-management and shared decision-making in a range of chronic health conditions [16], including hearing loss [17]. Access to and use of mobile devices are sufficiently well established, making this approach to health data collection feasible across all demographics in economies worldwide [18,19]. It is crucial that the design of usable apps is informed by the perspectives and priorities of the intended users regarding data collection and management, as well as the motivations and outcomes they associate with using the app [16]. This requires a deep understanding of the psychosocial context of these individuals and their views on potential intervention options. Qualitative research is recommended for gaining such an understanding [20].

The overall objective of this study was to gather information to guide the future co-design and development of a mobile app. This app aims to assist consumers with varying degrees of hearing loss in documenting their listening difficulties in real-world settings. The app development project has followed a systematic research road map [21]. Recent contextual inquiry into telehealth in audiology concluded that hearing service providers should further develop and expand telecare to align with client expectations [22]. Additionally, 2 separate focus groups with audiologists, distinct from the consumer focus groups discussed elsewhere, have underscored the clinical necessity of the information consumers would gather through such an app and share with their clinicians [23]. Our study clarifies the value proposition and functional requirements of such an app for consumers, laying the foundation for future co-design and prototype testing involving both stakeholder groups.

The objectives of this study were to explore, with consumers experiencing hearing loss, their:

- Experiences of listening difficulties, including what prompted them to seek hearing devices, how they assessed the benefit of hearing devices, and the specific information defining their listening difficulties, to identify data that an app could collect.
- Preferences regarding the features of mobile apps in general.
- Opinions on the potential value and desirable features of a yet-to-be-designed app for documenting listening difficulties in real-world settings.

## Methods

### Recruitment

The number of focus groups required to address a research question depends on its complexity. Researchers typically consider data saturation—the point where additional data no longer reveal new insights—to determine when enough focus groups have been conducted. The most common practice involves 2-4 separate groups with 6-12 participants each. Fewer than 6 participants may limit the discussion, while more than 12 can be challenging for the moderator to manage [24,25]. Consideration also needs to be given to the likelihood of some participants not showing up, as well as potential communication difficulties among participants. In the context of this study, the initial goal was to recruit 9 participants for each of the 3 focus groups, with an expectation of 1-2 no-shows per group. Additional groups would be planned if data saturation was not reached.

Purposive sampling was applied for focus group recruitment, as is standard practice [26]. This method involves selecting participants based on their ability to meet specific criteria—such as having relevant experience and knowledge—to contribute meaningfully to discussions on the topic. Participants are also chosen for their willingness to engage in such discussions. Random sampling was not utilized due to the small sample size, which precluded statistical analysis. Rather, the aim was to prevent unintended bias in the selection process by recruiting participants from various sources. It is recognized that focus groups have limitations due to their small participant numbers, nonrandom sampling, and the self-selection of participants, which restricts the generalizability of findings to the broader target population [27]. This limitation applies to this study, where participants were required to travel to a central city location and discuss their personal experiences of hearing loss, which can be a deeply personal topic for some individuals.

Participants were recruited from a variety of sources. An advertising flyer was distributed through the authors’ personal and professional networks, including social media accounts, colleagues from private and public audiology clinics, and University of the Third Age organizations, which offer learning opportunities primarily for individuals not in the workplace.

To recruit participants with diverse hearing histories, selection criteria were being 18 years or older and self-identifying as having hearing loss. While the duration of smartphone ownership was included as a demographic question and app use was discussed in the focus group, no related inclusion criterion was applied. The intention was to recruit participants with varying levels of smartphone and app experience. Table 1 presents the demographic information for the 27 participants. Each consumer chose their preferred date and time and participated in 1 of the 3 focus groups. All recruited participants attended as scheduled, with no no-shows. Further recruitment was unnecessary as data saturation was achieved. Participants provided written informed consent and received a gift voucher to cover travel expenses and acknowledge their time commitment.

**Table 1:** Demographic information for the 27 participants.

Age range and gender		Self-reported degree of hearing loss^a^	Hearing devices fitted; years of use	Years of smart device use
**20-29 years**				
	Male	Right: moderate-severe; left: severe	HAs^b^; 12	15
**60-69 years**				
	Female	Moderate-severe	HAs; 17	10
	Male	35%	HAs; 9	6
	Female	Severe-profound	HAs; 6	9
	Male	Profound	CIs^c^; 12	10
	Female	Moderate-severe	HAs; 10	7
	Female	Mild	None	10
	Female	Moderate	HAs; 4	10
**70-79 years**				
	Male	Mild	HAs; 5	5
	Female	Right: moderate-severe; left: moderate-profound	HAs; 8	10
	Female	Moderate-severe	HAs; 20	10
	Female	40%	HAs; 6	10
	Male	Moderate	HAs; 7	0
	Female	Mild	None	0
	Female	Right: severe-profound; left: profound	CI + HA^d^; 10	15
	Male	Moderate	HAs; 10	10
	Female	40%	HAs; 6	6
	Male	Mild-profound	None	5
	Female	Not known	HAs; 8	10
	Female	Moderate	HAs; 18	7
**80-89 years**				
	Female	40%	None	10
	Female	Right: mild-severe; left: mild-moderate	HAs; 4	4
	Male	Moderate	HAs; 10	5
	Male	Severe-profound	HAs; 10	15
	Female	Right: 68%; left: 32%	HAs; 40	0.5
	Male	75%	HAs; 0.5	15
	Male	Severe	HAs; 25	3

^a^Bilateral hearing loss unless otherwise specified.

^b^HA: bilateral hearing aid.

^c^CI: bilateral cochlear implant.

^d^CI in 1 ear and HA in the contralateral ear.

### Materials

Authors KGalvin, BHBT, and DT, each experienced clinical audiologist and hearing researcher, developed a topic guide for the focus group discussions to address the research objectives. The original text of the topic guide is provided as Multimedia Appendix 1. Following focus group 1, minor adjustments were made to enhance clarity and better guide the conversation, resulting in a revised version (Multimedia Appendix 2) used for focus groups 2 and 3. The guide included instructions for the facilitator to explain the purpose and structure of the session, along with introductions for each subtopic. Subtopic 1 (Hearing Devices and App Use) aimed to stimulate discussion on factors influencing the decision to seek hearing devices, how consumers assessed their effectiveness, and their general preferences regarding mobile apps. Subtopic 2 (Listening Difficulties) aimed to generate discussion on the specific details and impacts of participants’ listening difficulties. Both subtopics 1 and 2 aimed to indirectly gather information relevant to the future design of an app by exploring the criteria consumers used or would consider using when making decisions about hearing devices and assessing their listening difficulties. Subtopic 3 (Assessment of Listening Difficulties via an App) was more direct, seeking consumers’ ideas on the potential value and features of a future app. The topic guide included specific prompts under each subtopic for the facilitator to generate and guide discussion in relevant directions. The discussions prompted by the first 2 subtopics were also expected to inform the future co-design of an app. For example, conversations about the information consumers used when deciding to get hearing devices are relevant to developing questions that future app users might consider when assessing their own need for hearing devices.

As part of the session conduct explanation, the topic guide included a set of basic “rules of engagement” (eg, 1 speaker at a time) to encourage active participation from all attendees. This was crucial, given the varying hearing levels and hearing device fittings among participants, as well as the communication challenges often faced by individuals with hearing loss in group settings. To enhance accessibility, a PowerPoint (Microsoft Corporation) slide deck was used to present key information in written form, including the current topic of discussion as per the topic guide. Multimedia Appendices 3 and 4 contain the original and slightly revised versions of the slide decks, which aligned with the topic guides used in focus group 1 and focus groups 2 and 3, respectively.

As shown in the topic guides in Multimedia Appendices 1 and 2, the facilitator described the concept for the yet-to-be-designed app in general terms. For example: “We aim to create an app that serves as a data collection tool, capturing real-life situations and individuals’ listening experiences in those situations,” and “We envision this data aiding joint decision-making regarding hearing aid fitting and measuring change or benefit over time.”

### Procedures

The 3 focus groups were conducted in June and July 2019. Using the topic guide, the facilitator (KGalvin) explained the use of the overhead slides, provided a high-level introduction to the focus group’s purpose, and outlined the “rules of engagement.” The facilitator then used the topic guide to stimulate discussion and followed the directions set by the participants when appropriate. Each 1-hour session was video- and audio-recorded. Observers included 2 Masters of Clinical Audiology students and at least one coauthor who occasionally contributed to the discussion.

### Data Analysis

The student observers transcribed the discussions verbatim from the video and audio recordings, ensuring to redact any information that could identify individuals (eg, names) or potentially identify them (eg, names of clinics attended). The video recordings were not utilized in subsequent stages of data analysis. The draft transcriptions were then imported into NVivo (Version 12, 2018; QSR International Pty Ltd) qualitative data analysis software. ZM, an audiologist with experience in hearing aid fitting and a PhD student studying consumer experiences related to real-world listening difficulties, reviewed the audio recordings and refined the draft transcriptions to create the final versions. During this process, care was taken to ensure that all identifying or potentially identifying information had been redacted.

The 3 transcripts were analyzed together using inductive qualitative content analysis, a method previously applied in hearing science. This method primarily categorizes manifest content—what the text explicitly states—to generate a descriptive overview of the data [28]. An inductive approach allows themes to emerge directly from the transcripts, making it suitable when there is limited existing knowledge about the investigated phenomenon. The content areas were defined based on the topics outlined in the topic guide. ZM reviewed the transcripts and excluded parts irrelevant to the study objectives. Using inductive content analysis, relevant sections of text were identified, coded, and categorized. This iterative process involved relistening to session recordings, re-reading transcripts, and revising codes and categories as new insights emerged. KGalvin then reviewed and revised the transcripts and codes. Subsequently, ZM, KGalvin, BHBT, and DT met to discuss the codes and categories, achieving consensus on the inclusion and coding of text sections.

### Ethics Approval

Approval for this study was provided by The University of Melbourne ethics committee (ID 1953773).

## Results

### Subtopic 1: Hearing Devices and App Use

#### Early Indicators of Hearing Loss and Prompts to Seek Hearing Devices

When asked about early indicators or missed signs of their hearing loss or listening difficulties, consumers reported difficulties in participating in group meetings, instances of mishearing leading to confusion or amusement, increased concentration demands, and speaking loudly:

If I was sitting in the wrong spot, and obviously I was unaware [of] how much lipreading I was doing, I couldn’t hear somebody at the other end of the table. I couldn’t hear the question. If I asked a question...I couldn’t hear the answer.

P1.2

We had a lot of confusion at home. There’d be things that I would distort that we’d have lots of laughs about.

P1.4

What I noticed is how much concentration I had to put in in certain situations. So, I’m working twice as hard as other people to keep up.

P2.9

When I was at work, they told me once I got my hearing aids my voice was a lot softer. I was considered to be really loud.

P3.1

Consumers also reported being in denial and ignoring feedback from others:

I was in denial. But I was getting all kinds of feedback about my hearing that I wasn’t paying attention to, not just from my wife, from other people [as well].

P2.2

The consumers were asked about their decision-making process in obtaining hearing devices, including the information they used and how they assessed the benefits. However, their discussion primarily centered on the factors that prompted them to seek hearing devices, which appeared to be a critical juncture in their hearing care journey. Consumers reported feeling prompted to obtain hearing aids due to significant spousal pressure, advice from others, hearing about others’ experiences, or observing behavioral examples from others:

My wife nagged me until I got hearing aids.

P2.3

I knew some people who had cochlear implants. [They] said to me, gee, I reckon you’re at the point where you’re [going to] benefit from implants. So, I talked to a couple of people, and I was persuaded actually pretty quickly. One of [my daughter’s] best friends...I could just see how amazingly she was doing in the playground with all the noise at school. I thought if she can make that work, I might...make that work.

P2.5

One person who I work with [who] had hearing aids said: “Your hearing is absolutely bad, do something”. I talked to some other people...[and] I bit the bullet and made a decision.

P3.7

The most common reason consumers cited for obtaining hearing devices was difficulties in speech comprehension, which included mishearing, frequently asking for repetitions, and relying on lipreading:

It got embarrassing to keep asking people to repeat. I thought “I need to get past that”. So I got them [hearing aids].

P2.8

I saw an audiologist eventually because I realised I was lipreading a lot.

P2.7

Speech comprehension difficulties with specific speakers, such as children and spouses, and in particular settings, including background noise, group settings, and meetings, were also identified as reasons for obtaining hearing aids:

What’s really prompted me into action [is] that grandchildren are starting to talk and they are down low, and they speak in high pitched frequencies and they mumble a bit and I can’t understand.

P3.2

The reason I got them...was...I found it difficult to participate in a group situation.

P1.2

There are many situations in which I couldn’t hear, particularly in restaurants, where there is background noise...Where there were lots of extraneous noises, like air conditioning or heating, it was very troubling.

P2.6

If I was sitting in the wrong spot, I couldn’t hear somebody...at the other end of the table, I couldn’t hear the question. And...if I asked a question, if somebody responded that was a bit further away, I couldn’t hear the answer.

P1.2

Consumers also mentioned difficulty comprehending speech transmitted via telephone or television as a reason for obtaining hearing aids:

I started having problems...hearing the television.

P2.8

I decided to get something done...when I couldn’t understand anybody on the telephone.

P1.9

Many consumers identified the workplace as the setting where they found the consequences of their speech comprehension difficulties to be most unacceptable, prompting them to obtain hearing aids:

If you are a professional...you...realise the ethics of your working in a situation where you are not hearing everything and that becomes very compromising of your profession and other people that you’re trying to help.

P1.4

I just had to hear the students because I was doing a lot of outreach and I had to interact.

P3.3

...[W]hen I was a school administrator, I just couldn’t stand going into the staff room. I always had a lot of criticism that I was the kind of person who didn’t get out there and go and talk to them. I couldn’t. [There were] one hundred women talking at once.

P1.4

For some consumers, it was the social isolation resulting from their speech comprehension difficulties that prompted them to obtain hearing aids:

It’s the social isolation that you suffer, missing out on conversations and what have you.

P3.2

Aside from speech comprehension difficulties, experiences while listening to music were also identified as a reason for obtaining hearing aids:

I started to go to [classical music] concerts and I wanted to hear more.

P3.8

#### Mobile Apps in General

Among the 27 consumers, 25 owned a smart device, with an average ownership duration of 8.7 years (SD 3.9; Table 1). Consumers utilized apps for data storage, entertainment, accessing information, booking services, and connecting with others.

Consumers noted that appealing apps were those designed for specific purposes and available for free download. Once installed, these apps were appreciated for being user-friendly, not consuming excessive storage or battery life, and for providing reminders. Visual presentation of information was particularly valued by these adults with listening difficulties, who preferred reading and seeing information rather than hearing it:

I’ll choose to look at something visual always. If I can see words, that’s what I’ll go for.

P1.4

Consumers reported that they discontinued using apps that were either not useful or had become outdated and unusable. Among those who did not use apps, some cited being too busy, considering them unnecessary, or lacking knowledge of how to use them:

I haven’t got around to it. I’ve got a computer [that] does everything I need

P2.2

Technology bewilders me.

P2.1

### Subtopic 2: Listening Difficulties

#### Personal Definition and Contributing Factors

Consumers were asked about their understanding of the term “listening difficulties” and what challenges they faced while listening. They were also queried on what information could aid in better comprehending their own listening difficulties or comparing them with others. However, no direct answers were provided to these inquiries.

Consumers overwhelmingly focused their discussion on speech comprehension when defining “listening difficulties.” They reported that characteristics such as a person’s accent, speed, volume, or pitch of speech could significantly impact comprehension:

Accents are a problem.

P1.1

Some people talk very quickly, and it makes it very difficult, because when they talk slowly you can take time to concentrate

P2.3

...[I]t’s the actual pitch. I find some of the guys I work with I can’t hardly hear them and some I can hear really clearly even if they speak softly.

P1.8

I can’t hear the grandchildren.

P1.6

Speech comprehension was also more difficult if the speaker’s face was not fully visible:

If men feature a moustache, forget it.

P1.3

It also seems to be necessary to see the face of the person who’s speaking and almost lipread.

P2.1

Aside from the visibility or characteristics of the speaker, consumer discussions on listening difficulties primarily centered around aspects of the acoustic environment that hindered speech comprehension. These included factors such as reverberation; distance from the speaker; and the presence of ambient noise, music, or other people talking:

Before [my hearing loss] I would go anywhere. [Now I] look at the room size, is there going to be an echo issue? Echo seems to be a problem for many people with hearing loss. Are there hard surfaces? Is there carpeting?

P2.6

If people are far away [I don’t hear].

P1.3

So often it’s the subterranean noises that impact, like refrigeration in a cafe or air conditioning popping on and off.

P2.6

You get ten people in a room. Even [if] you [have] got just two people talking...I can’t hear.

P1.9

...[W]e have a break for tea and coffee...and I have no idea what people are talking about. They can be right next to me...The noise from everyone talking makes it impossible.

P1.7

...[T]he chemist was talking to me but I couldn’t hear her, even though I was facing her, because there was conversation behind me.

P1.5

Consumers identified an inability to focus on a single speaker when many people were talking as a significant contributor to their speech comprehension difficulties:

...[T]he inability to filter. If I remember...when I could hear, many people talking in a café and you could hone in [on] the person talking to you, but now it’s like a cacophony of galahs to me.

P1.3

Consumers also reported difficulties comprehending transmitted speech:

Even with the hearing aids and even with the phone on volume [I have to say] “Can you repeat that?”

P1.1

I really can’t listen to the radio.

P1.6

[I] find it impossible to hear when people use a microphone.

P1.7

Airports. A nightmare. They’re announcing: “Group A gets in the plane”. Well, you have no idea who’s getting in the plane.

P1.9

When I go to church, I find it very difficult because the speaker system is highly directional and depends on where I sit.

P2.2

Consumers reported experiencing the greatest difficulties when multiple factors, such as group conversations in restaurants or speech transmitted with background music, combined to create particularly challenging situations for speech comprehension:

Even with my family all sitting around, I find it very difficult.

P1.7

I find in restaurants [it’s] impossible. I was sitting next to someone recently, and I said I can see your lips moving, but I have no idea what you are saying.

P2.9

I have to turn the captions on when I’m watching television, because they put all this stupid music on, even when they’re reading the news.

P2.3

When discussing what listening difficulties meant to them, consumers also mentioned the challenges they faced in locating sound sources and how this affected their safety and ability to participate in activities:

I have no sense of direction. I can’t tell whether the fire is there or down there.

P3.3

My husband’s a birdwatcher, and he hears something and he knows exactly where to look at it, and I am still sort of [searching]...Loss of localisation of sound is significant.

P3.5

#### Impacts and Consequences

In addition to discussing the factors that made listening difficult, consumers spoke about the impacts and consequences of these difficulties, particularly in relation to their relationships and employment:

My wife complains. She says, “I have to tell you things ten times”.

P2.2

I’m quite sure it led to my divorce. Because I never heard what he said.

P3.3

I think hearing is so integral to our relationships...If we don’t get it right, we’re frustrating everybody. I get sick of saying “What did they say?”

P2.9

One of the big problems [is that] people talk to me and I walk away, and they’re standing behind me. I’ve had people snub me later on because they think I’ve snubbed them, and I haven’t heard them.

P3.3

And I think if someone’s going to say something confidential. And sometimes it can be...a loving thing that someone wants to say quietly...then they lower their voice and then I can’t get it!

P1.4

...[I]t’s affected my employment. I’ve had massive problems.

P1.1

Consumers also reported that social isolation and withdrawal were consequences of their listening difficulties, along with other negative emotions such as stress, embarrassment, sadness, irritability, and frustration:

I miss all the jokes, that’s the worst thing.

P1.7

Like being at work, and not being [included], kind of left out of some talk in the lunchroom.

P3.4

And you think, I won’t ask the person to repeat it now, especially if this is a group context, I’ll try and catch up. And sometimes the conversation has gone [on] for five minutes [and] I haven’t got back into the conversation at all.

P2.5

And waiting to speak because you’re not quite sure whether you got the right end of the stick there.

P3.4

In the beginning I was really trying to keep up with everybody; over time I’m finding I’m switching off totally.

P2.8

I must admit I don’t [go out socially] much anymore because I just don’t enjoy it.

P1.1

It saddens me that I don’t lie in bed and listen to talkback radio and conversations on the ABC like my partner does.

P1.4

I have to keep saying, “Sorry I missed that. Sorry I missed that,” and it’s rather embarrassing.

P1.8

You can’t hear very well, [you] get grumpier...I think...Irritable.

P3.4

[It’s] very frustrating being a grandad and not be[ing] able to hear what your granddaughter is saying.

P2.8

In addition to negative emotions, consumers reported that their listening difficulties led to fatigue:

[The] degree of concentration that one needs to engage in, in order to understand what somebody is saying; [there’s] exhaustion and fatigue after a difficult hearing context.

P2.5

#### What the Clinician Needs to Know and Understand

Consumers were asked what their clinicians needed to know about their listening difficulties and how they could effectively convey their listening experiences. However, they did not directly address these questions. Instead, they proposed and discussed the viewpoint that hearing care appointments focused less on the client’s experiences and more on hearing aid sales and adjustments. Additionally, consumers discussed reasons why a clinician’s understanding of their clients’ listening difficulties might be limited:

They’re busy concentrating on the screen and doing the adjustments and getting them right, and not listening to what the client’s saying.

P1.9

Another thing that aggravates me, you’re in this really quiet little room where they’re doing their adjustments, and you think “Yeah, that’s great”. And you’re out in the real world outside the door, and [you think] “Hell, that’s worse.”

P1.9

Consumers also identified some ways clinicians could better understand their clients’ listening difficulties. These included actively listening to and believing the client, and setting up the client to provide useful feedback:

[They need] to listen [to] what you’re saying...and believe what you’re saying. It’s the client that’s got the ears and that’s having the difficulty.

P1.9

[It would be better if the audiologist] said...“Come back in two weeks [and] I want you to remember the good and the bad situations” rather than [the audiologist] saying [after two weeks] “How’re you going?” and [the client] thinking “Oh [I don’t know]”.

P1.8

### Subtopic 3: Assessment of Listening Difficulties via an App

Consumers were broadly supportive of the concept of an app that they could use to document their listening difficulties in real-world settings:

It would certainly help. When you get hearing aids for the first time, you’re not really aware of what things you should be looking at. But if a professional came up with like a checklist...that would be really helpful, because that would be a more formal way. And you wouldn’t be just remembering, “Oh when I was down the street, I heard this sort of thing”.

P3.2

If you were to set [it] out in terms of situations, it would be useful.

P3.4

I think we should be more responsible for our own health and actually think about it and not just put it all on [the audiologist].

P1.8

When asked for their input on how an app should function, consumers expressed a desire for flexibility and user choice regarding how and when data are collected, entered, and shared. They considered notifications prompting them to collect or enter data into the app to be valuable:

If you’re prompted, you can always choose to ignore it. But if you [are] prompted with things, you’ve probably [a] higher chance that you’ll actually use it.

P3.2

Consumers generally agreed that users should have control over the presence, number, and timing of notifications to collect or enter data into the app. They also felt that users should be able to define the real-world settings in which they would document their listening difficulties:

It would be useful. And you could give the title to the situations yourself...Whatever was important to you in making life bearable.

P3.4

Consumers were asked what information should be collected or entered into an app to describe real-world settings where listening difficulties were experienced. They suggested that information about the listening environment should include the type of physical space, factors affecting reverberation (eg, carpet), background noise, the number of people in the conversation, visibility of the speaker’s face, and distance from the speaker. Consumers also wanted information about the listener’s physiological and emotional state, as well as their behavioral responses to listening difficulties, to be entered into the app. This includes levels of listening effort, fatigue, frustration, unhappiness or discomfort, and social withdrawal:

I think emotions play a huge part [in] what you want to hear, and what you do hear.

P1.9

Tiredness can be a result of your hearing problem, but being tired then also impacts on what you’re hearing once you’re tired.

P1.1

If I feel frustrated in a hearing situation, I’d like to be able to press a button and capture that for the audiologist, because I don’t know what’s causing it.

P2.2

I think [the] level of happiness and unhappiness is something that makes a situation memorable, because whether you go back to it again, or not, is often determined by that...Me being uncomfortable, or not liking the situation, is sort of quite important to record.

P3.4

The other thing is just giving up. Just going quiet and sitting there but not participating because you think, “Hmm, ok, I can’t hear”.

P2.9

There was extensive discussion about the methods an app could use to collect data and the formats through which users could input information. Opinions on the use of audio recordings and photographs varied; some consumers felt it should be straightforward for clinicians to envision a particular listening situation described by the client, while others believed that a photo would offer valuable details:

I don’t think that’s really [valuable] because if you can’t envisage a room with ten people around a table having a having a meal...Why do you need a photo [to understand that]?

P1.9

...Is the floor polished concrete, are there carpets, are there drapes, [is there a] high ceiling, low ceiling, [are there] sound-reflective surfaces, sound-absorbing [surfaces]? I think there’s probably quite a bit of information that someone might glean from that in a listening situation.

P3.2

Consumers who endorsed the use of photographs acknowledged that video recordings would offer more comprehensive information, though concerns about the privacy of other individuals were raised. During the discussion on data collection methods for the app, there was recognition of the importance of balancing the effort required of the app user with the value of the information gathered.

Opinions varied among consumers regarding the effectiveness of rating scales using numbers or descriptive words, with or without the option for open-text comments versus solely relying on open-text descriptions. Some consumers found open-text descriptions to be more time-consuming. However, most consumers supported the inclusion of a 1-5 rating scale with descriptive words, alongside an open-text option for providing additional information.

In terms of the period of app use, most consumers considered a 2-week period to be appropriate for using the app to ensure that their listening difficulties could be documented across a variety of real-world settings.

Consumers identified that forgetting to input data and the effort required to use the app would be barriers to its use. They also mentioned that receiving feedback on the data collected or entered would serve as a motivation to use the app:

I’d like to have some feedback...I’m prepared to enter as many times as required, as long as I get the feeling that someone’s actually listening.

P1.2

Two types of feedback were suggested. Some consumers believed it would be beneficial for an app to provide acknowledgment of the data collected or entered, such as summarizing the frequency of data collection or the actual data entered (such as ratings of listening difficulties). One consumer suggested that the app could include congratulatory statements when targets for data collection or entry were achieved. Another consumer thought it would be valuable for the app to deliver feedback from the individual’s clinician:

It would be really good if there was...an actual message from the clinician [so] that you knew the data was actually going [somewhere].

P1.4

Generally, consumers agreed that there would be value in an app sharing information with the user’s clinician, as long as the information was appropriately considered by the clinician.

Aside from the primary function of documenting listening difficulties and sharing data with clinicians, consumers also proposed additional functions for the app, including recording communication partner feedback, documenting communication breakdowns, and evaluating the benefit of assistive listening devices.

## Discussion

### Principal Findings

The topic guide was crafted to prompt discussion on consumers’ experiences with listening difficulties, their preferences for app features in general, and their opinions on the features of a forthcoming app designed to document listening difficulties. The overarching goal is to leverage insights gained from these focus groups to guide the future co-design and prototype development of a mobile app aimed at helping consumers document their real-world listening challenges. As anticipated, the findings help clarify the value proposition and functional requirements of such an app.

Consumers in this study identified a lack of awareness regarding the severity and impact of their listening difficulties, as well as resistance to accepting feedback about these difficulties from communication partners. This lack of self-awareness has been recognized in other studies as a barrier to seeking help [29,30]. Once hearing aids have been fitted, the perception that they provide no benefit has been identified as the most common reason for their nonuse, according to a scoping review [10]. This highlights a clear need for a tool that enables consumers to document and track their own listening performance in real-world settings. Such a tool can help raise self-awareness of listening difficulties, understand their impacts and consequences, and assess the effectiveness of interventions in real-world scenarios.

Consumers in this study expressed broad support for using an app to document real-world listening difficulties, noting several benefits: (1) it would provide a guided and structured approach to data collection, which is especially valuable early in the hearing care journey when individuals may be unsure of what to report to clinicians; (2) it would alleviate the need to remember all relevant real-world experiences for later reporting to clinicians; and (3) it would empower consumers to take more responsibility for their own health. Importantly, preliminary testing of this approach in a recent study provided evidence of both acceptability and efficacy. In the study, 29 older adults used a smartphone app to collect real-world listening experiences, and the findings indicated that the approach was user-friendly and resulted in increased awareness and positive discussions regarding hearing loss [31]. Consumers in this study indicated that having a list of questions to prompt them to recall listening experiences in various settings, or even a prompt to recall both positive and negative experiences prospectively, would be beneficial when describing their listening difficulties to their clinician. This aligns with the primary goal of the proposed app, which aims to provide consumers with a tool to document their listening difficulties in real-world settings, thereby eliminating the need to recall experiences over extended periods such as weeks or months. This need for objective and subjective data logging aligns with current perspectives on the global potential of apps to support effective personal informatics systems for well-being [32].

The general app characteristics valued by consumers (purposeful, limited use of device storage space, low battery consumption, free, easy to use, and up-to-date) have been identified as influential factors in the adoption of mobile health services [33]. For consumers with listening difficulties, access to visual information was particularly important to reduce reliance on hearing. It is crucial for health care apps to have a clear purpose that meets the needs of consumers and is intuitive to use, especially for those who may not be familiar with app usage or who consider them unnecessary. This principle is particularly relevant for mobile apps designed specifically for deaf and hard-of-hearing consumers. The study on app quality for deaf and hard-of-hearing consumers revealed a high turnover rate and identified a lack of high-level features [34]. The authors concluded that essential features necessary for this demographic were often overlooked or poorly implemented, resulting in limited scope for available apps.

Regarding features of an app to document listening difficulties, consumers emphasized flexibility as crucial. They viewed it as highly desirable for an app to offer choices in documenting listening difficulties across various real-world settings. The need for such documentation to be tailored to settings important to the individual has long been recognized in the field. Fixed-item questionnaires such as the Satisfaction with Amplification in Daily Life Scale [35] have utilized co-design processes to achieve this customization. By contrast, other questionnaires such as the Client Oriented Scale of Improvement [36] have allowed individuals to identify a small number of personally important real-world settings. A digital tool offers enhanced flexibility, allowing users to customize both the number and type of real-world settings they document. The consumer preferences identified in this study align with critical factors observed across various health and care apps, including design customization, cost-effectiveness, information validity, privacy and security protection, personalization capabilities, and ease of use [37,38].

Insight into the information an app could effectively gather was derived from discussions about listening difficulties (including early indicators, personal definitions and experiences, contributing factors, impacts, and consequences), as well as direct responses to queries about the specific details an app should capture regarding individual listening situations. Alongside providing a means to document the type and degree of listening difficulties in real-world settings, consumer discussions indicated that it would also be beneficial for an app to collect data about the speaker, the environment, and the listener. The data related to the speaker and environment could include factors well-known to affect listening comprehension, such as speaker accent, number of speakers, background noise, and reverberation. The consumer discussion highlighted the importance of collecting listener-related data for an app, focusing on the physiological and emotional states of the listener, as well as their behavioral responses to listening difficulties. Consumers noted that physiological and emotional states both contribute to and are impacted by listening difficulties. For instance, they mentioned that fatigue can exacerbate listening challenges, while difficult listening situations themselves can lead to fatigue. The impact of physiological and emotional states on listening difficulties is often disregarded in objective testing and inadequately addressed in the administration of subjective questionnaires. Questionnaires typically solicit ratings or evaluations that depict “typical” listening performance without adequately considering the diverse factors that variably influence listening challenges. Some questionnaires, such as the Hearing Handicap Inventory for the Elderly (HHIE) [39], include inquiries about the emotional effects of hearing loss (eg, Does a hearing problem make you irritable?) and specific listening scenarios (eg, Does a hearing problem cause you to feel embarrassed when meeting new people?). However, these questionnaires do not explore the correlation between physiological and emotional states and listening difficulties (eg, does a higher level of embarrassment experienced when meeting new people result in greater listening difficulties?). This is the type of information consumers wanted the app to collect to enhance their understanding of these relationships. Regarding behavioral responses to listening difficulties, consumers emphasized that the key response they wanted the app to document was withdrawing from social interactions when these difficulties arose. They deemed this crucial as it signaled when listening challenges became unmanageable.

The consumer discussion underscored the critical importance consumers placed on the broader and longer-term impacts and consequences of their ongoing listening difficulties. Therefore, in addition to collecting data related to specific real-world settings, consumers expressed that there would be value in an app gathering data on several aspects: the frequency and intensity of negative emotions stemming from ongoing listening difficulties, the impact of these difficulties on relationships and job performance, and the extent of social withdrawal and isolation experienced as a result. Such data would enhance consumers’ understanding of their emotional, physiological, and behavioral responses to their experienced listening difficulties over time. This understanding would empower consumers to communicate effectively about their listening difficulties with their clinician, prioritize intervention options available to them, and assess the outcomes of interventions using personally meaningful measures.

Consumers had varying opinions on how data should be collected or entered into an app, underscoring the need for the app to offer multiple options. Following the consumer discussion, the proposed app should gather listening difficulty data over up to 2 weeks. It should feature a 5-point scale with numerical and categorical labels, along with an open-text field for users to detail their experiences of listening difficulties. Additionally, the app should allow for photography, video recording, and audio recording of the settings where these difficulties occur. Moreover, the app should, at a minimum, offer feedback that acknowledges the data entered or collected. An identified barrier to app use was the perceived effort involved; therefore, providing flexibility in how data are collected and entered would allow users to determine when the benefits outweigh the effort required. Consumers expressed support for sharing data with their clinicians and believed that clinician feedback through the app could serve as a motivational factor for its use. Sharing app-collected data would also enhance the clinician’s understanding of the consumer’s personal experiences with real-world listening challenges. The evidence suggests that facilitating access to professionals is a crucial design feature in mobile health apps [38]. Adoption and continued use of health apps can be challenging, with a complex mix of influential factors. A recent review of sociotechnical factors highlighted the need for a more patient-centered approach to enhance usability and overcome barriers [33].

In this study, 26 of the 27 consumers were aged 60-89 years. This age distribution reflects the higher prevalence of listening difficulties among older adults, attributed to poorer hearing thresholds and auditory processing challenges [40], as well as the limited availability of working-age individuals to participate in focus groups. Beyond hearing barriers, older adults are recognized as having specific preferences and needs concerning mobile health apps [41]. The findings of this study align with existing knowledge and can inform the co-design of a prototype app that takes into account cognitive and physical abilities, motivation, and perceptual issues crucial for usability.

Separate focus groups involving a total of 10 audiologists were conducted to gather clinicians’ perspectives on the concept and desirable features of an app for consumers to document listening difficulties [23]. The main categories identified in the analysis of these clinician focus groups were (1) the type of data the app would collect; (2) potential additional features; and (3) benefits. The findings from consumers were largely consistent with those from clinicians, although clinicians identified additional data points for collection (eg, information to aid in selecting appropriate models of care for individual clients), additional functions (including goal setting, tracking changes in auditory lifestyle, training, advice, and prompts to enhance listening performance), and additional benefits (such as data-driven clinical decisions, improved long-term management, increased clinical efficiency, strengthening therapeutic relationships, support between appointments, and identifying the need for counseling). Recognizing the numerous barriers to integrating health apps into clinical practice [42], it is crucial to ensure that clinicians can identify potential clinical benefits and efficiencies from an app idea before the co-design phase.

### Conclusions

Consumers expressed strong support for a mobile app designed to help them document their listening difficulties, as well as the impacts and consequences in real-world settings. The findings of this study offer valuable guidance for developing a prototype app, outlining the types of data that describe consumers’ listening experiences and can be collected via an app, along with essential design principles. The information describing listening-related experiences included early or missed indicators of listening difficulties, such as mishearing, difficulty communicating in groups and on the phone, and speaking overly loudly. It also covered prompts to seek hearing devices, such as spousal pressure, advice or examples provided by others, and the need to rely on lipreading or constantly request others to repeat themselves. Consumers also discussed well-known factors that influence listening difficulties, such as reverberation, background noise, and group conversations. They also highlighted the impacts and consequences of these difficulties, including negative effects on relationships and employment, social isolation and withdrawal, and negative emotions. This information will be instrumental in guiding the future co-design of a mobile app aimed at collecting data relevant to users’ real-world experiences of listening difficulties. Such an app aims to enhance both self-awareness and clinician understanding. In terms of broad design principles, consumers expressed a preference for a visually oriented app that offers flexibility in how data are collected and entered, tailored to the individual’s real-world settings. Consumer support for the app concept and its desired features aligns with findings from separate clinician focus groups [23]. With insights gathered from these consumer and clinician discussions, there is now a comprehensive understanding of the context and value needed to formulate a development brief for a prototype app. This marks the next stage in the CeHRES (Centre for eHealth Research) road map for developing eHealth technologies [21]. A future prototype will undergo co-design and development in an iterative process involving both consumers and clinicians. This process will validate the conclusions drawn here through real-life user experience testing.

## Appendix

Multimedia Appendix 1 Topic guide used in focus group 1.

Multimedia Appendix 2 Slightly revised topic guide used in focus groups 2 and 3.

Multimedia Appendix 3 Original PowerPoint slide deck used during focus group 1.

Multimedia Appendix 4 Slightly revised PowerPoint slide deck used during focus groups 2 and 3.

## Abbreviations

CeHRES: Centre for eHealth Research
HHIE: Hearing Handicap Inventory for the Elderly
