# Supplementary material for: Consumer Perspectives for a Future Mobile App to Document Real-World Listening Difficulties: Qualitative Study
Source: JMIR Form Res. 2024 Jul 23;8:e47578. doi: 10.2196/47578 (PMC11303898; doi:10.2196/47578)
Supplement: Multimedia Appendix 1 [file formative_v8i1e47578_app1.docx]

**Multimedia Appendix 1:** Original topic guide used in focus group 1

The non-italicised text is the gist of what the facilitator will say – with key points displayed on overhead projector for the benefit of people with greater degree of hearing impairment. The italicised text will be used as prompts for discussion as required.

### Introduction (5 minutes)

Thank you for coming along today. We really appreciate your willingness to participate in our research.

I will introduce my co- researchers: Dani Tomlin, and Barbra Timmer.

Dani and Barbra will be taking notes so that we have a summary of the key points in our discussion.

And we have two student audiologists with us too: Dora Lee, and Hai Trinh. Dora and Hai will be managing the recording, so we have a detailed record in case we can’t remember all of the important things that were discussed.

Housekeeping: tea and coffee, bathroom location, taking your own notes, key information on the overhead projector, we’d like everyone to be able to participate (variety of levels of hearing impairments and hearing devices among you, please speak up and speak clearly, just one person speaking at a time (facilitator will direct this), and ask for repeats if you need)

What we would like to do today is to learn from you so that we can get an understanding of what people with hearing loss think would be a helpful direction for us to take in our app development research. To do that, we would like you to tell us about your experiences and opinions. If you wish, you can also talk about the experiences of other people you know with hearing impairment. We will be asking some questions, but they are just to guide the discussion to the areas relevant to our research. There are no right or wrong answers to these questions, and we hope that there will be a variety of answers because this will show us we have a broad range of viewpoints.

### Hearing devices and app use (15mins)

We’ll start off with some general discussion about hearing devices and apps: what you choose to use - or choose not to use - and why.

*Can you tell us about your hearing device use – have you used them in the past and decided not to continue, do you use hearing devices now, or you have never used any hearing devices?*

*How did make your decisions about hearing devices?*

*Do you use – or have you ever used - any mobile devices, such as smart phones or iPads?*

*And what do you use them for?*

We are trying to explore why people do or don’t use apps. For those who aren’t familiar with what an app is or how it works, it is a small pieces of computer software that allow you to do specific tasks of all kinds on your mobile device, most often connected to the Internet - for example, book a taxi, share a photo with friends, see the current weather report, record your mood, etc.

*For those who use who don’t use apps, can you tell us a bit about why? And for those people who do use apps, can you tell us why you use certain apps, and if there’s other that you don’t use for a particular reason?*

### Our aims for the app + discussion of listening difficulties (20mins)

As you know, our research is about developing a mobile device app. We hope to make an app which is a data collection tool. A tool which collects data about the real-life situations a person is in each day, and their listening experiences in those situations. And we want to collect that data from the person at the time they are in those situations. It is our intention for the app to collect data that accurately reflects listening difficulties so that the individual and their clinician will have a better understanding of the person’s listening experiences. We hope that this data will help joint decision making about hearing aid fitting and the measurement of change or benefit over time.

*What does the phrase “listening difficulties” mean to you?*

*What situations have there been when you wish your clinician was with you? And what information would you like them to have so they can better understand what listening is like for you in the real world?*

*What information do you think would help you to get a better understanding or measure of your own listening difficulties as and when these arise for you?*

*What sort of information would you need, to compare your own listening difficulties to those of someone else with or without a hearing loss?*

*What did it take time for you to understand about your own listening difficulties?*

*What is the likelihood that other people around you were also having listening difficulties in a particular situation you were in?*

*What makes a situation a difficult listening situation? Anything about the environment, or the people you are communicating with, or how you feel in that situation?*

### Break (5 min)

### Assessment of listening difficulties - now & via an app (45 mins)

Today a hearing care clinician may get information about a person’s listening difficulties by asking them questions, giving them a survey to fill out, and testing their hearing levels and speech understanding in the test booth. There are problems with this approach because testing may only happen once, the testing is not in real-life situations, appointment times can be short, and the person must rely on their memory to answer the questions or complete the survey. Thinking about this, and what we have discussed already, if we were to develop an app which provided better or more useful data about a person’s listening difficulties in real-life, what data do you think the app should collect, and how should it collect that data?

We are most interested in your own ideas and not what we think might work. But just to generate discussion we have a few suggestions about what data and how to collect it.

*How would you prefer to assess your own listening difficulties: with a numbered rating scale, or by providing a description in your own words, or something else?*

*What aspects of a listening situation would you like to be able to report on? What information would give some context to your assessment of your own listening difficulties?*

*How useful (or not) would it be if you could report your listening difficulties at any time in a situation that you choose, or should the app request your input?*

*What do you think about an app that was able to take photos of listening situations?*

*What do you think about an app that was able to audio-record listening situations?*

*What do you think about an app that was able to record how you are feeling about communicating in the particular listening situation you are reporting on?*

*What do you think about an app that was had an option to upload the data to your clinician?*

*Over what length of time should an assessment of your listening difficulties occur: over a short time - 1 hour? - or over a longer time so that you experience different listening situations – 1 day? 1 week?*

*How many times and how often would you be prepared to answer the same question so that the data collected is reliable?*
