# Supplementary material for: Consumer Perspectives for a Future Mobile App to Document Real-World Listening Difficulties: Qualitative Study
Source: JMIR Form Res. 2024 Jul 23;8:e47578. doi: 10.2196/47578 (PMC11303898; doi:10.2196/47578)
Supplement: Multimedia Appendix 2 [file formative_v8i1e47578_app2.docx]

# **Appendix 2:** Slightly revised topic guide used in focus groups 2 and 3.

The non-italicised text is the gist of what the facilitator will say – with key points displayed on overhead projector for the benefit of people with greater degree of hearing impairment. The italicised text will be used as prompts for discussion as required. The strikethrough text indicates the deleted text from the original topic guide. The underlined text indicates the added text from the original topic guide.

### Introduction (5 minutes)

Thank you for coming along today. We really appreciate your willingness to participate in our research.

I will introduce my co- researchers: Dani Tomlin.

Dani will be taking notes so that we have a summary of the key points in our discussion.

And we have two student audiologists with us too: Dora Lee, and Hai Trinh. Dora and Hai will be managing the recording, so we have a detailed record in case we can’t remember all of the important things that were discussed.

Housekeeping: tea and coffee, bathroom location, taking your own notes, key information on the overhead projector, we’d like everyone to be able to participate (variety of levels of hearing impairments and hearing devices among you, please speak up and speak clearly, just one person speaking at a time (facilitator will direct this), and ask for repeats if you need)

What we would like to do today is to learn from you so that we can get an understanding of what people with hearing loss think would be a helpful direction for us to take in our app development research. To do that, we would like you to tell us about your experiences and opinions. If you wish, you can also talk about the experiences of other people you know with hearing impairment. We will be asking some questions, but they are just to guide the discussion to the areas relevant to our research. There are no right or wrong answers to these questions, and we hope that there will be a variety of answers because this will show us we have a broad range of viewpoints.

### Hearing devices and app use (15mins)

We’ll start off with some general discussion about hearing devices and apps: what you choose to use - or choose not to use - and why.

*~~Can you tell us about your hearing device use – have you used them in the past and decided not to continue, do you use hearing devices now, or you have never used any hearing devices?~~*

*~~How did make your decisions about hearing devices?~~*

*After you discovered that you had a hearing loss, how did you make the decision about whether or not to try hearing devices? What sorts of information helped you to make the decision?*

*How did you get the information you needed to help you make a decision about whether to get hearing aids?*

*If you think back to the time when you were first getting used to hearing aids, how did you decide if the hearing aids were helping you?*

*What information did you use*

*What other information would have helped you to decide?*

*Do you use – or have you ever used - any mobile devices, such as smart phones or iPads?*

*And what do you use them for?*

We are trying to explore why people do or don’t use apps. For those who aren’t familiar with what an app is or how it works, it is a small piece of computer software on your smartphone that allows you to do specific tasks of all kinds on your mobile device, most often connected to the Internet - for example, book a taxi, share a photo with friends, see the current weather report, record your mood, etc.

*For those who don’t use apps, can you tell us a bit about why?*

*For those who do use apps, What makes an app appealing? What makes an app unappealing? Why might you stop using an app that you’ve downloaded?*

### Discussion of listening difficulties (20mins)

From a clinician’s point of view, we often feel that we’re missing some fundamental information for understanding what your listening difficulties are. We’ve got your hearing graph, we talk to you and ask questions, we know you find it hard when it’s noisy and ok when it’s quiet, but the world is a lot more complicated than ‘noisy,’ ‘quiet,’ ‘TV,’ so we feel like we’re missing a step to actually understanding on an individual level whether to recommend hearing aids, or to adjust your hearing aids, or whether to recommend new hearing aids or move on from hearing aids and look at cochlear implants. We want a better understanding of your needs.

*What does the phrase “listening difficulties” mean to you?*

*What sorts of things does your clinician need to know to better understand what listening is like for you in the real world?*

*How could you convey to your clinician what listening is like for you?*

*What information do you think would help you to get a better understanding or measure of your own listening difficulties?*

*What sort of information would enable you to compare your own listening difficulties to those of someone else with or without a hearing loss?*

*Is there anything about your own hearing or listening difficulties that took time for you to notice or fully understand?*

*Other than noise, what makes listening difficult for you? Aspects of the environment, or the person or thing you are listening to, or the way you feel in that situation?*

### Break (5 min)

### Assessment of listening difficulties - now & via an app (45 mins)

Today a hearing care clinician may get information about a person’s listening difficulties by asking them questions, giving them a survey to fill out, and testing their hearing levels and speech understanding in the test booth. There are problems with this approach because testing may only happen once, the testing is not in real-life situations, appointment times can be short, and the person must you rely on their memory to answer the questions or complete the survey. Thinking about this, and what we have discussed already, if we were to develop an app which provided better or more useful data about a person’s listening difficulties in real-life, what data do you think the app should collect, and how might collect that data?

We are most interested in your own ideas and not what we think might work. But just to generate discussion we have a few suggestions about what data and how to collect it.

*Imagine that you’ve been asked to assess your own performance in various listening situations*

*What would you hope an app might be able to do? What features could be in an app that could help overcome some of those issues (hearing test happening in a booth or remembering how you’ve performed in the outside world in order to answer a questionnaire)?*

*If you imagine that you’re rating your own performance in a particular listening situation, what would be the best way to record your rating: numbered rating scale, “Extremely difficult, very difficult, somewhat difficult” options, writing a description, etc?*

*What aspects of a listening situation would you like to be able to report on? What information would give some context to your own rating of your listening performance? What information would help someone else to understand why some situations are more difficult than others?*

*How useful (or not) would it be if you could report your listening difficulties at any time in a situation that you choose? How would you feel if an app chose when to remind you to report your listening difficulties?*

*What do you think about an app that was able to take photos of listening situations?*

*What do you think about an app that was able to audio-record listening situations?*

*What do you think about an app that was able to record how you are feeling about communicating in the particular listening situation you are reporting on?*

*What do you think about an app that was had an option to upload the data to your clinician?*

*Think back to when you first came in for a hearing test or first noticed your hearing problems; if you were going to record an assessment of your listening difficulties, how long would you need to make observations? One day, one week, two weeks, one month? The longer the period the more likely you would experience a range of listening situations*

*How many times and how often would you be prepared to answer the same question so that the data collected is reliable?*
