# Supplementary material for: Consumer Perspectives for a Future Mobile App to Document Real-World Listening Difficulties: Qualitative Study
Source: JMIR Form Res. 2024 Jul 23;8:e47578. doi: 10.2196/47578 (PMC11303898; doi:10.2196/47578)
Supplement: Multimedia Appendix 3 [file formative_v8i1e47578_app3.pptx]

## Slide 1
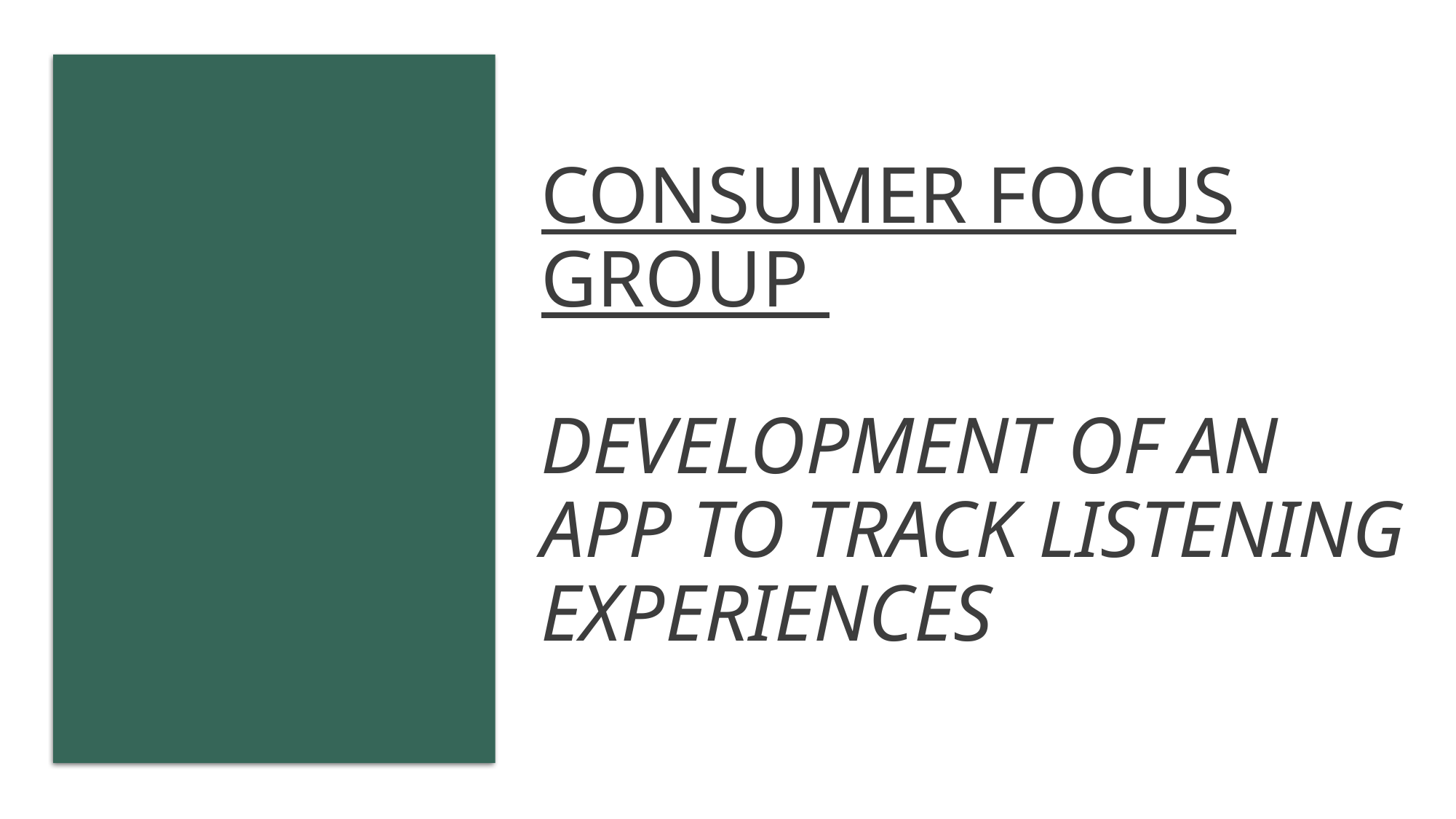

# Consumer focus group development of an app to track listening experiences

## Slide 2
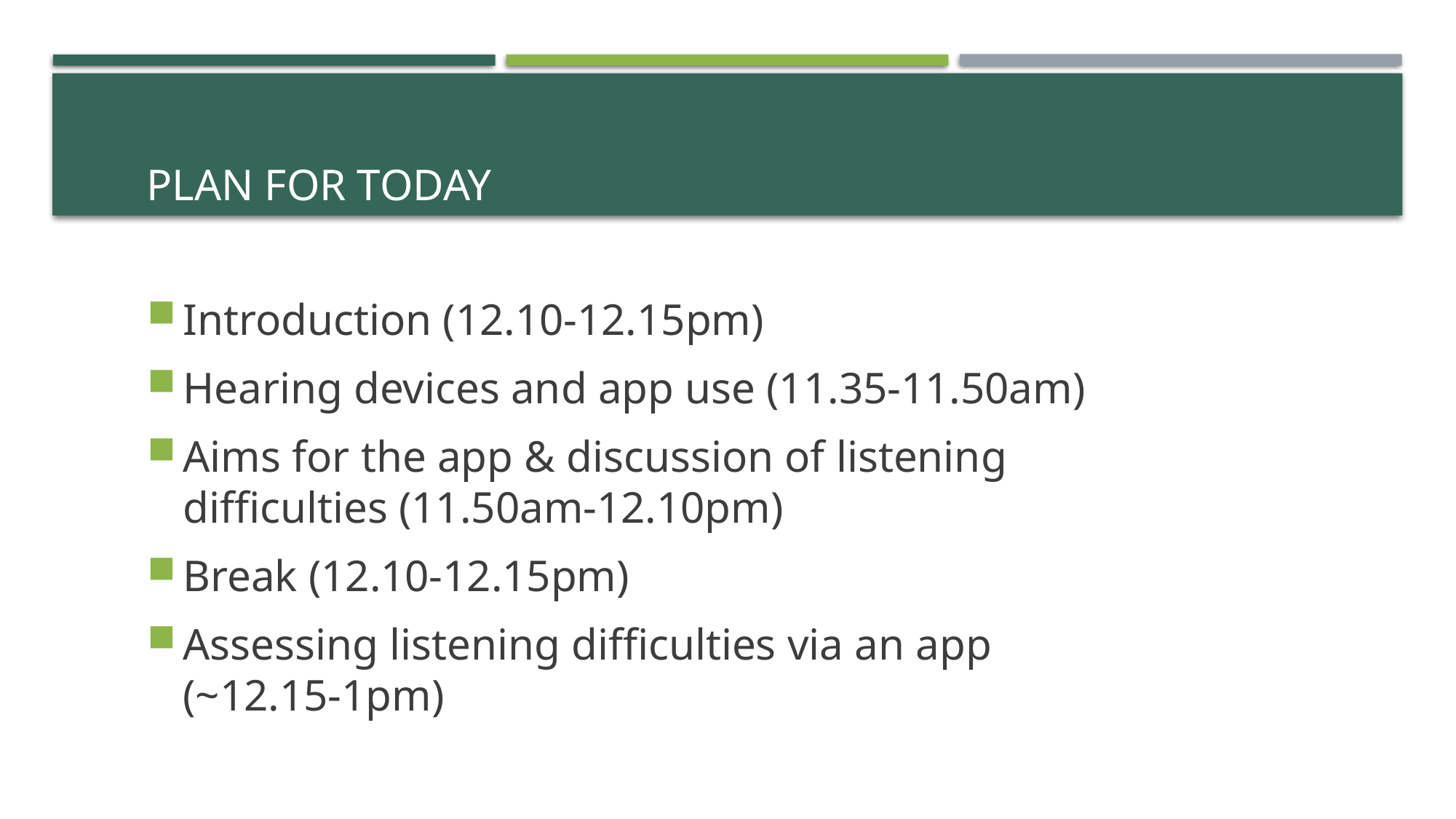

# Plan for today
Introduction (12.10-12.15pm)
Hearing devices and app use (11.35-11.50am)
Aims for the app & discussion of listening difficulties (11.50am-12.10pm)
Break (12.10-12.15pm)
Assessing listening difficulties via an app (~12.15-1pm)

## Slide 3
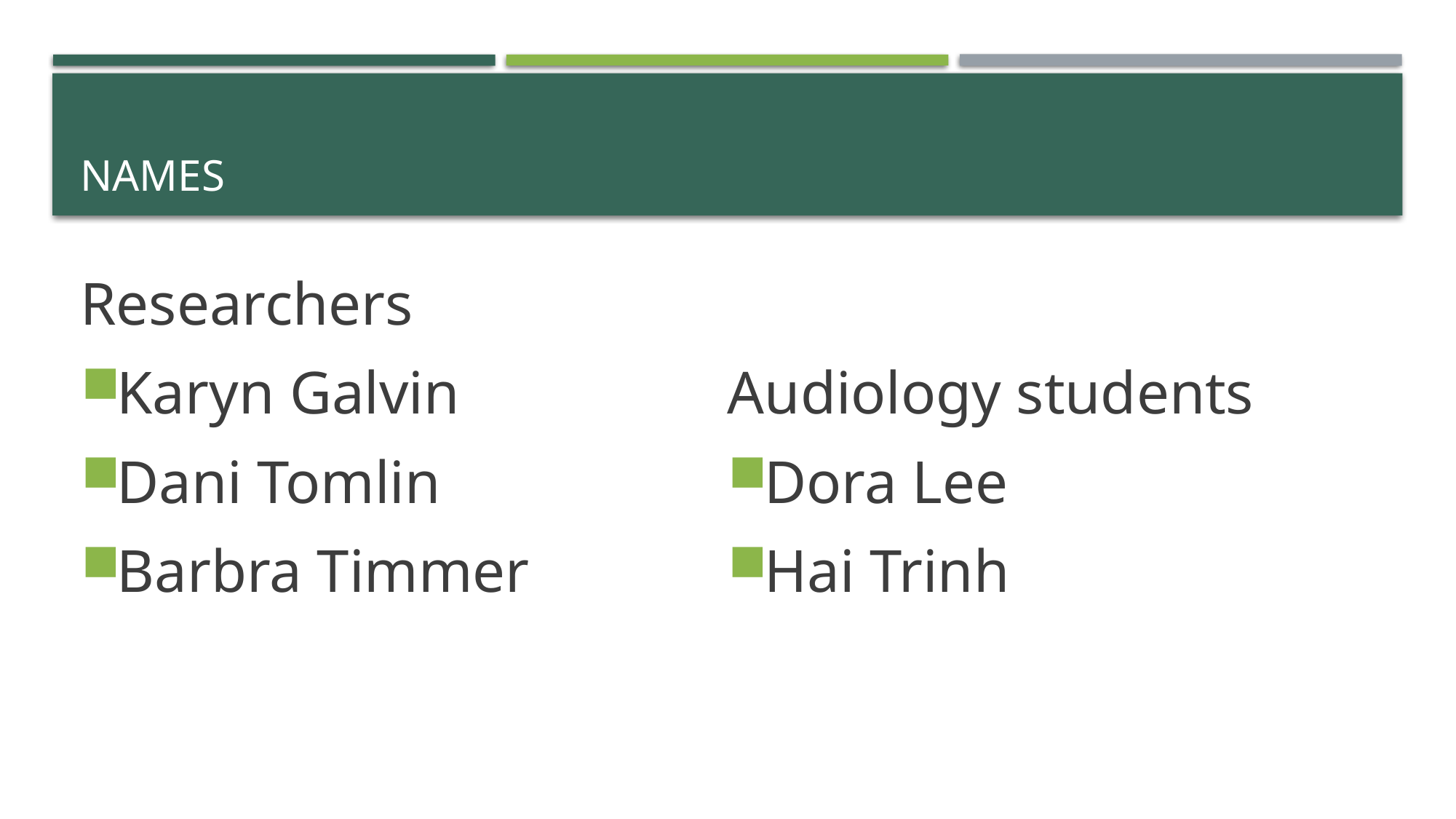

# Names
Researchers
Karyn Galvin
Dani Tomlin
Barbra Timmer
Audiology students
Dora Lee
Hai Trinh

## Slide 4
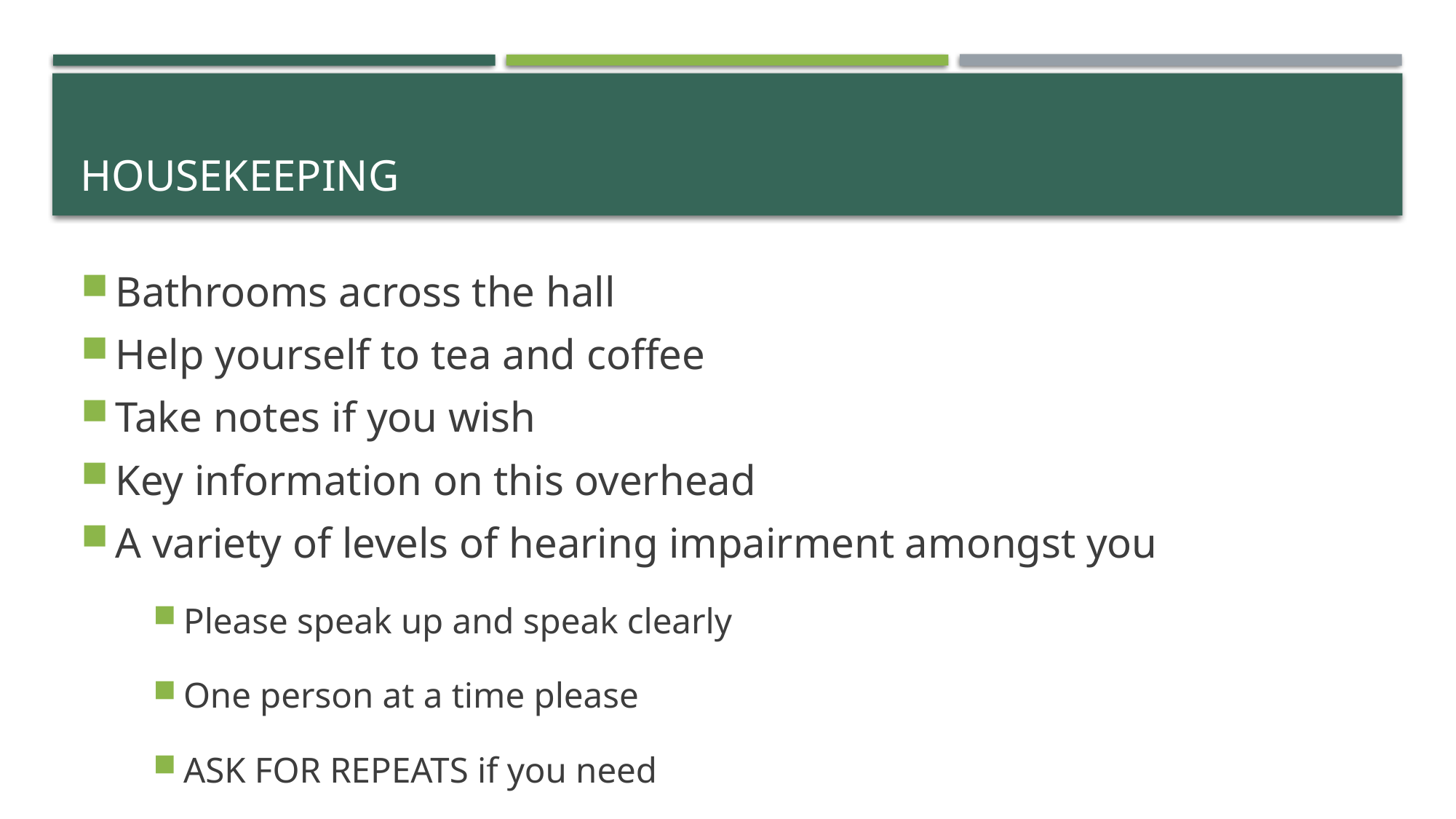

# Housekeeping
Bathrooms across the hall
Help yourself to tea and coffee
Take notes if you wish
Key information on this overhead
A variety of levels of hearing impairment amongst you
Please speak up and speak clearly
One person at a time please
ASK FOR REPEATS if you need

## Slide 5
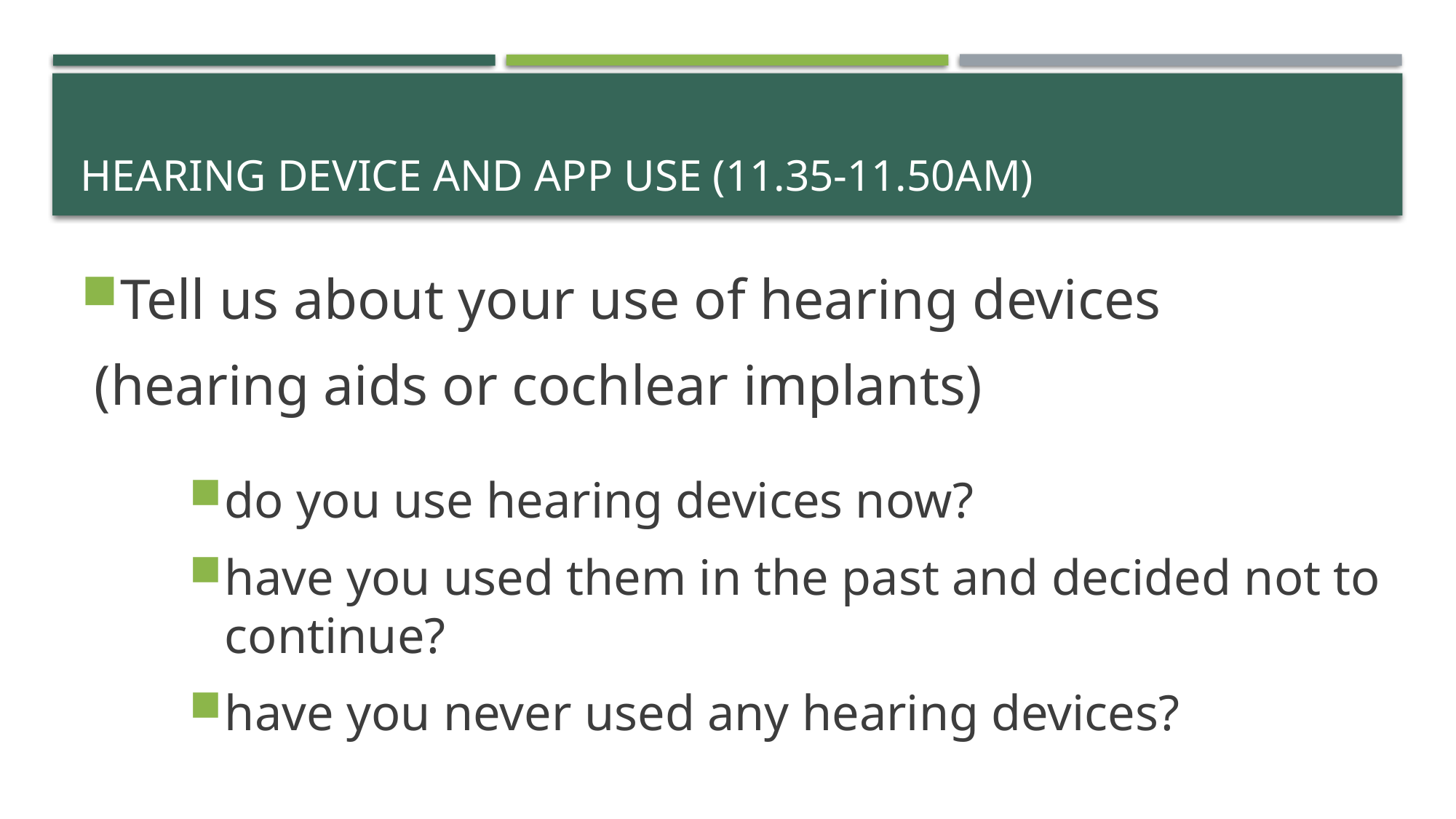

# Hearing device and app use (11.35-11.50am)
Tell us about your use of hearing devices
 (hearing aids or cochlear implants)
do you use hearing devices now?
have you used them in the past and decided not to continue?
have you never used any hearing devices?

## Slide 6
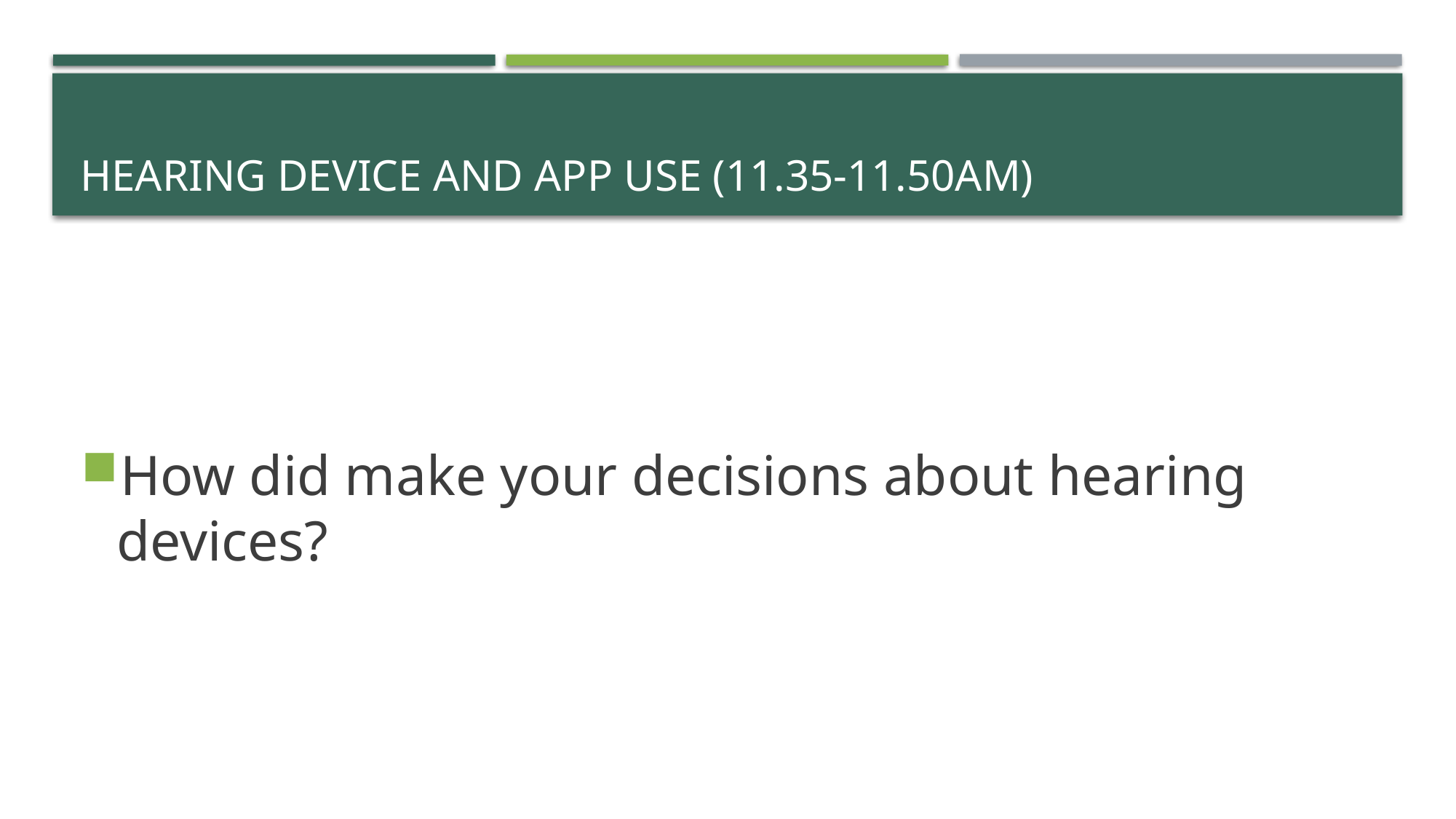

# Hearing device and app use (11.35-11.50am)
How did make your decisions about hearing devices?

## Slide 7
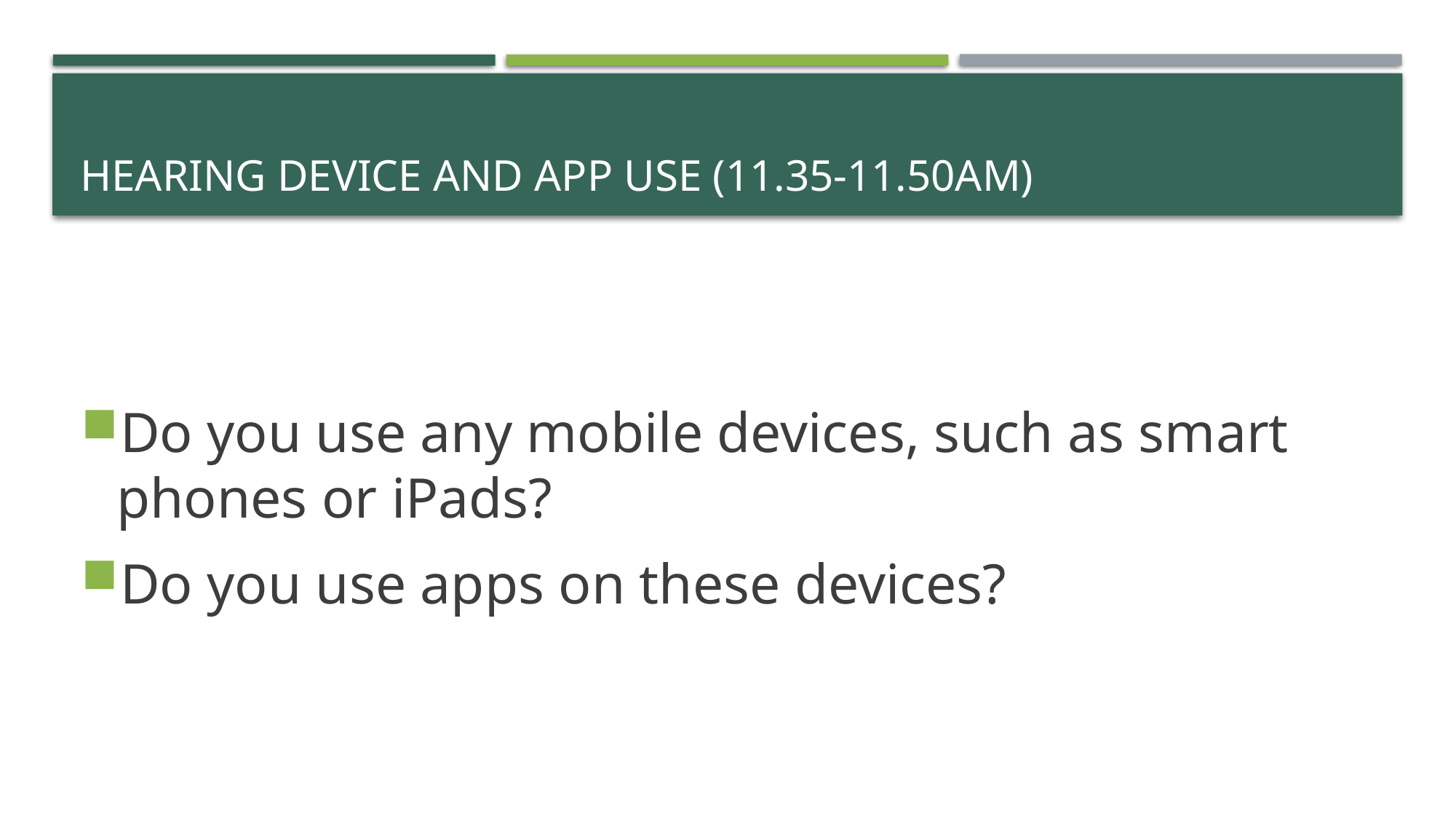

# Hearing device and app use (11.35-11.50am)
Do you use any mobile devices, such as smart phones or iPads?
Do you use apps on these devices?

## Slide 8
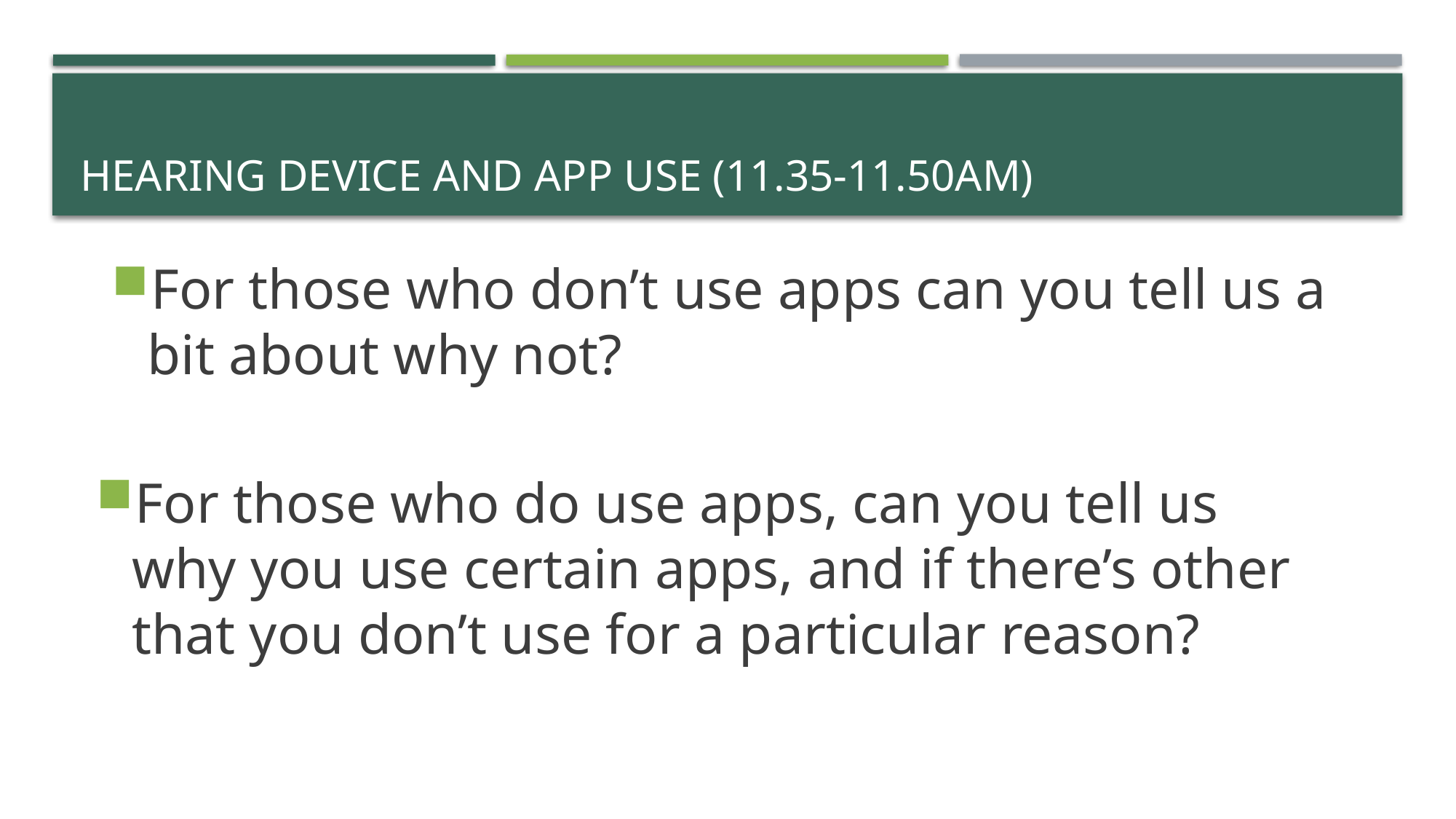

# Hearing device and app use (11.35-11.50am)
For those who don’t use apps can you tell us a bit about why not?
For those who do use apps, can you tell us why you use certain apps, and if there’s other that you don’t use for a particular reason?

## Slide 9
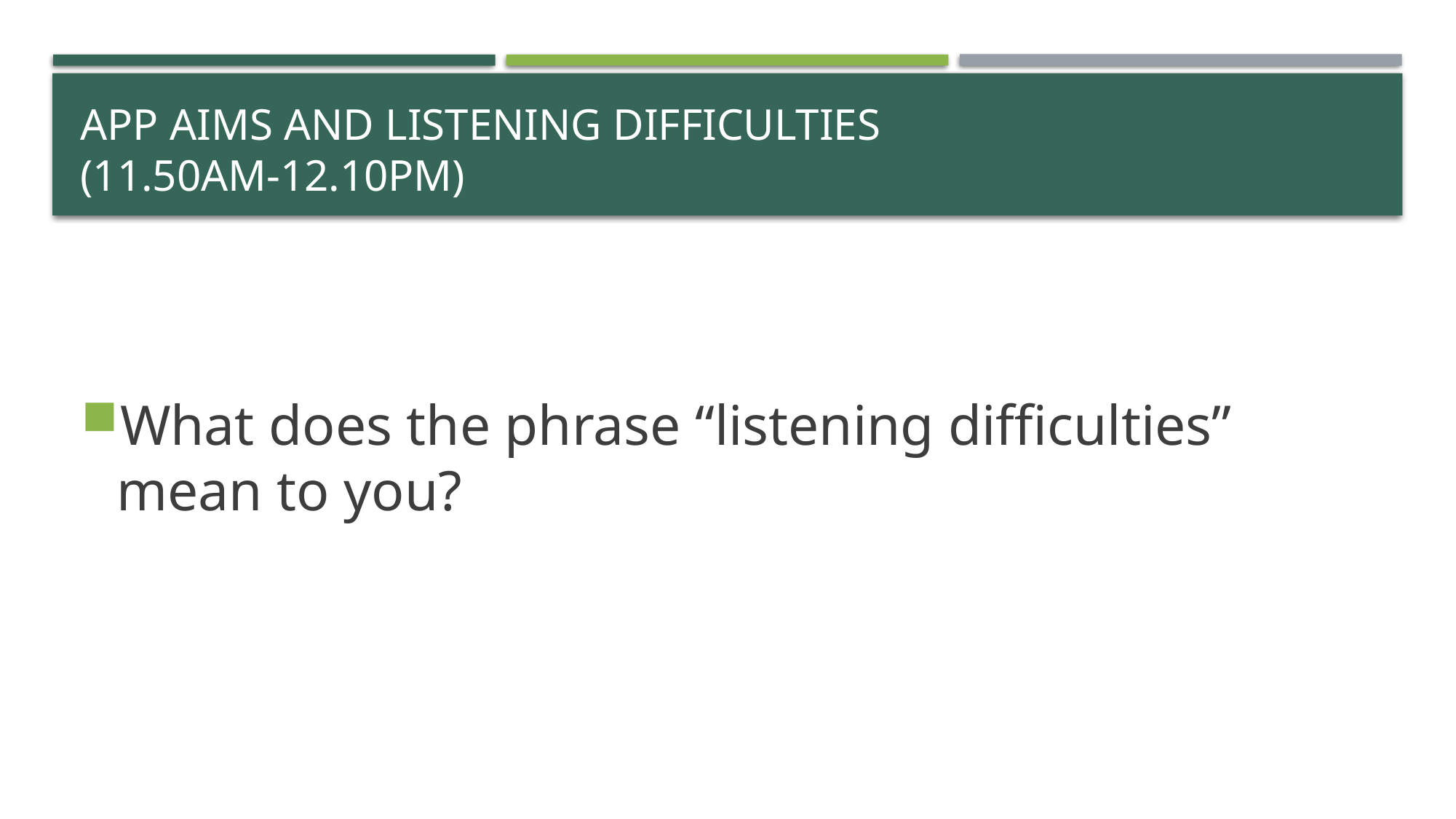

# App aims and listening difficulties (11.50am-12.10pm)
What does the phrase “listening difficulties” mean to you?

## Slide 10
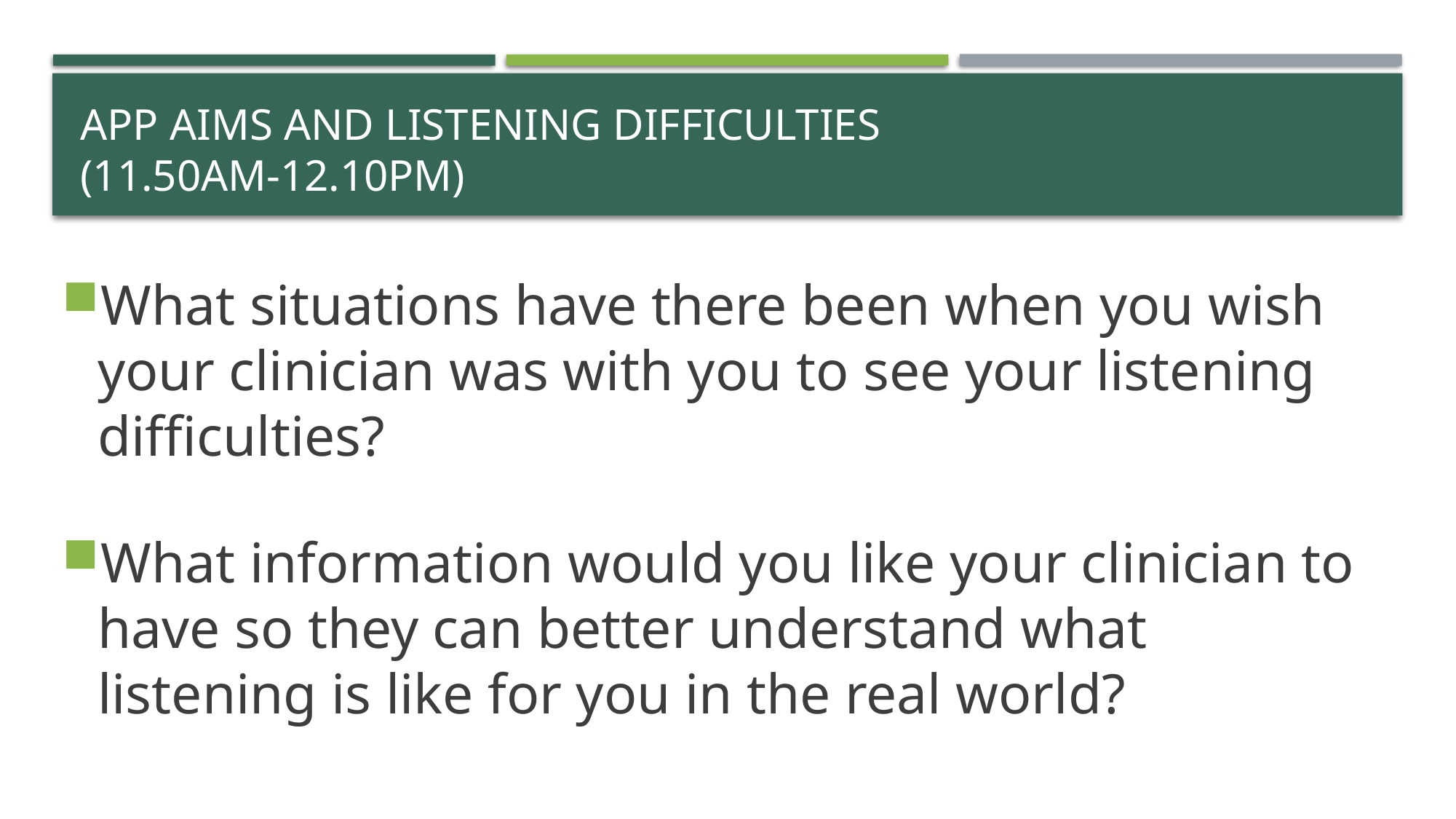

# App aims and listening difficulties (11.50am-12.10pm)
What situations have there been when you wish your clinician was with you to see your listening difficulties?
What information would you like your clinician to have so they can better understand what listening is like for you in the real world?

## Slide 11
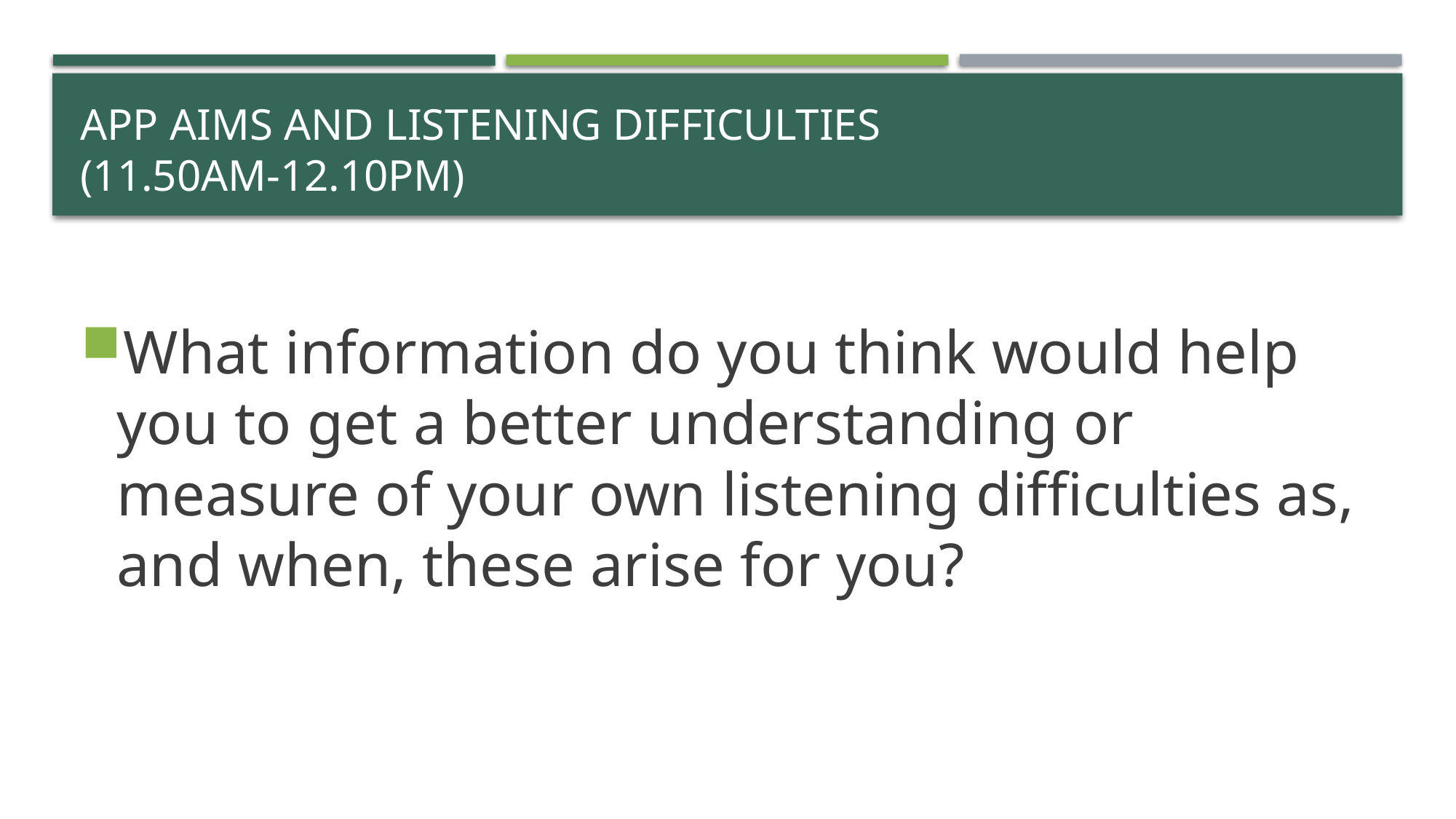

# App aims and listening difficulties (11.50am-12.10pm)
What information do you think would help you to get a better understanding or measure of your own listening difficulties as, and when, these arise for you?

## Slide 12
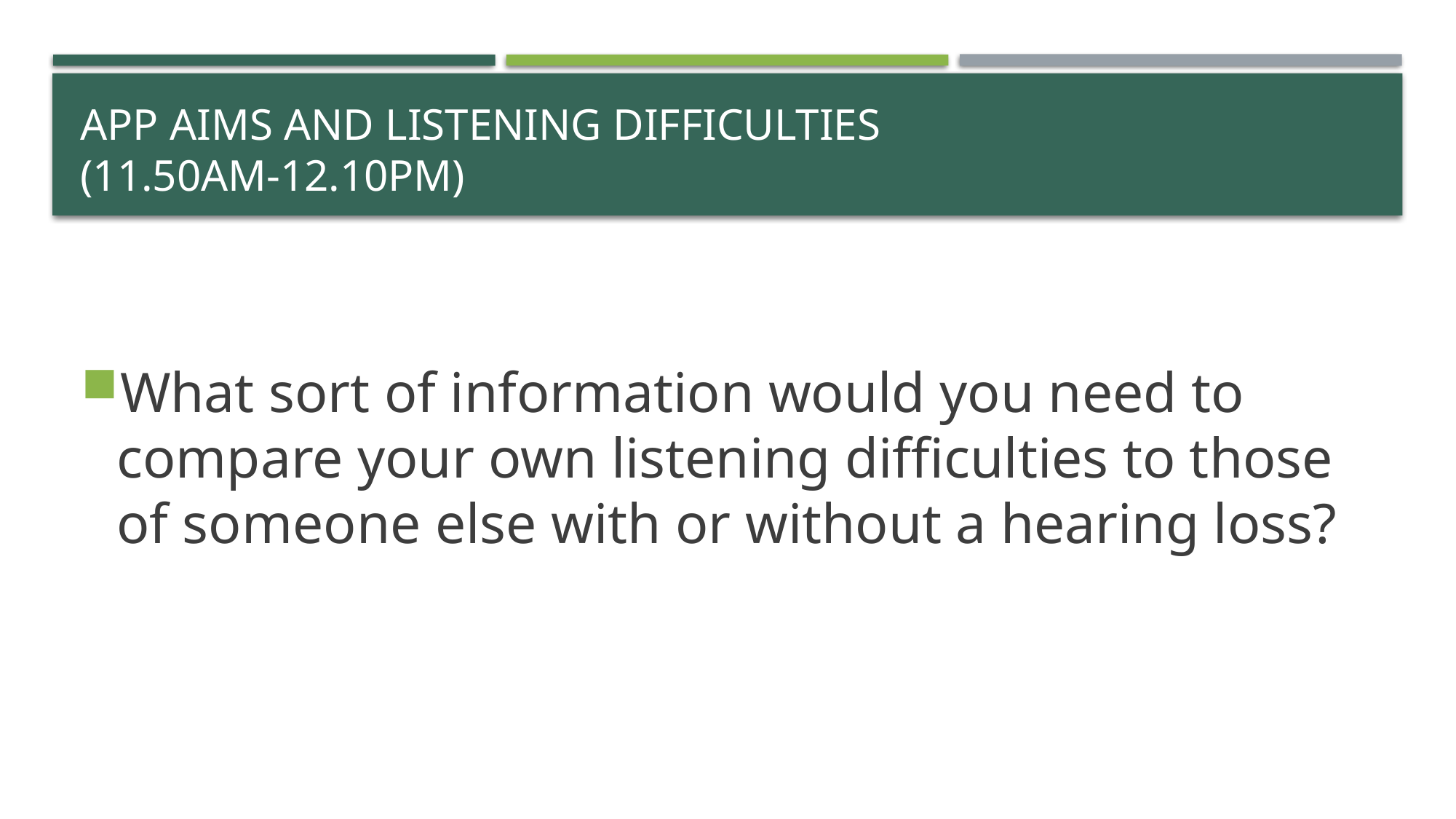

# App aims and listening difficulties (11.50am-12.10pm)
What sort of information would you need to compare your own listening difficulties to those of someone else with or without a hearing loss?

## Slide 13
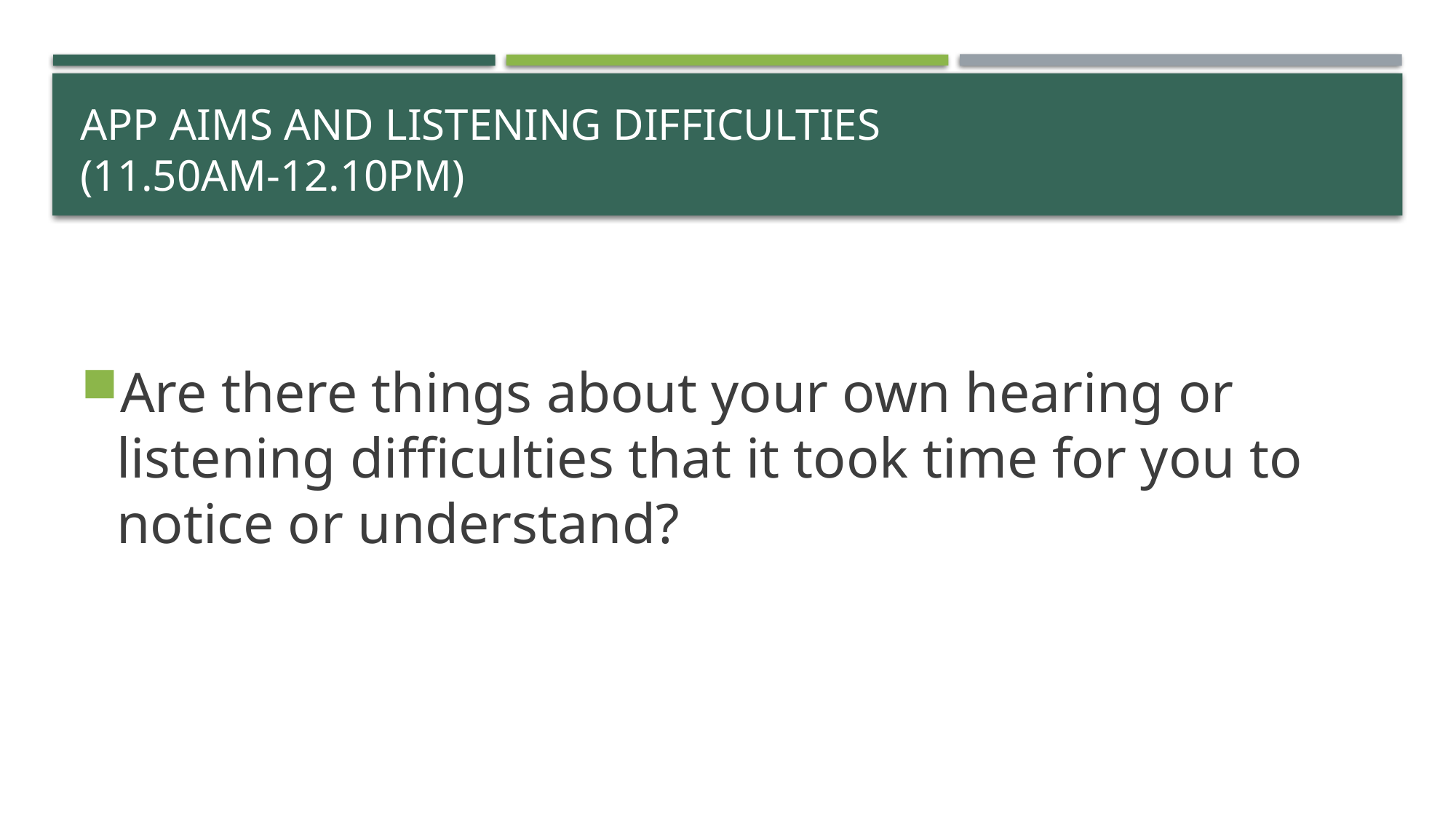

# App aims and listening difficulties (11.50am-12.10pm)
Are there things about your own hearing or listening difficulties that it took time for you to notice or understand?

## Slide 14
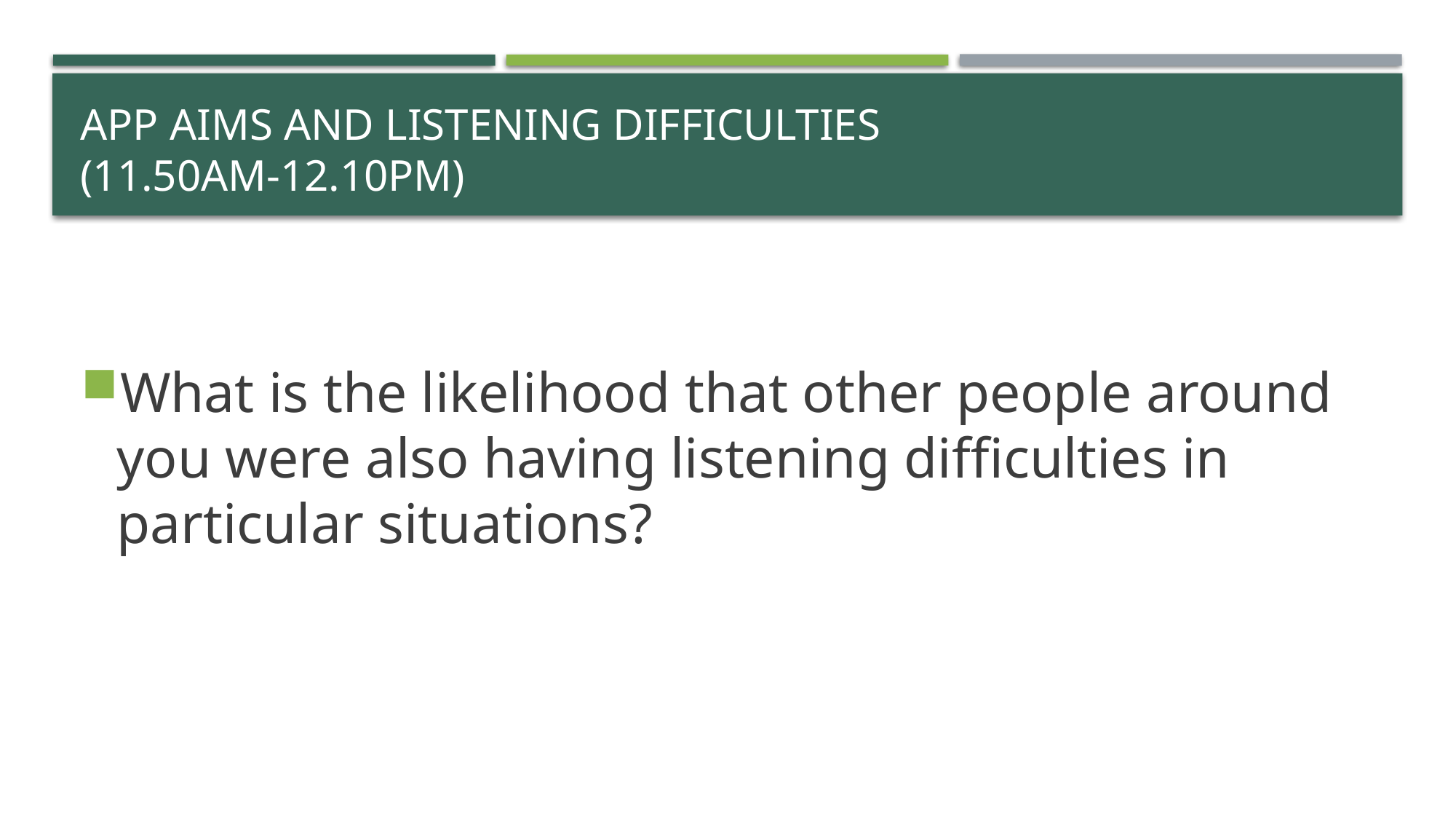

# App aims and listening difficulties (11.50am-12.10pm)
What is the likelihood that other people around you were also having listening difficulties in particular situations?

## Slide 15
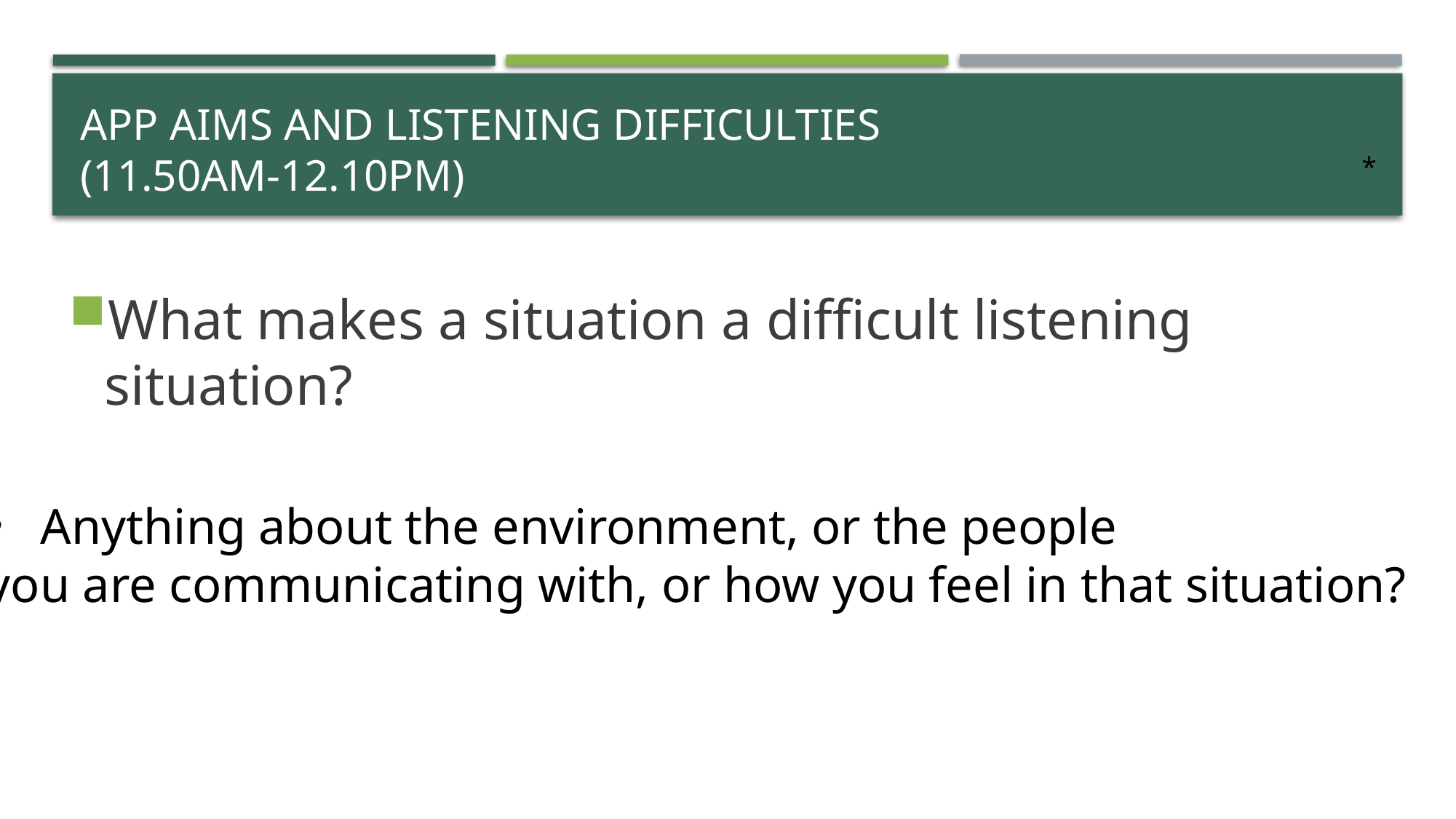

# App aims and listening difficulties (11.50am-12.10pm)
What makes a situation a difficult listening situation?
*
Anything about the environment, or the people
you are communicating with, or how you feel in that situation?

## Slide 16
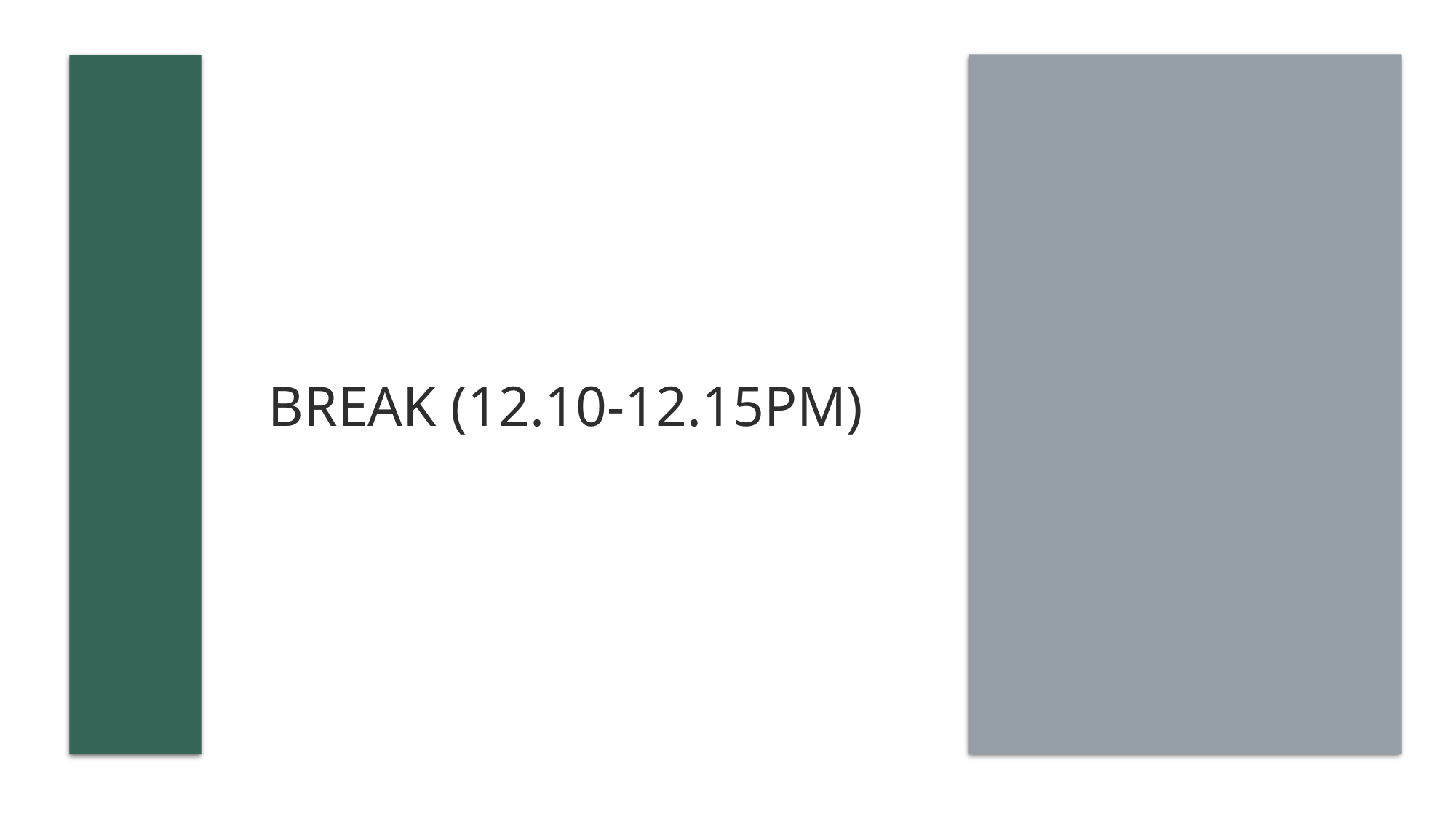

# Break (12.10-12.15pm)

## Slide 17
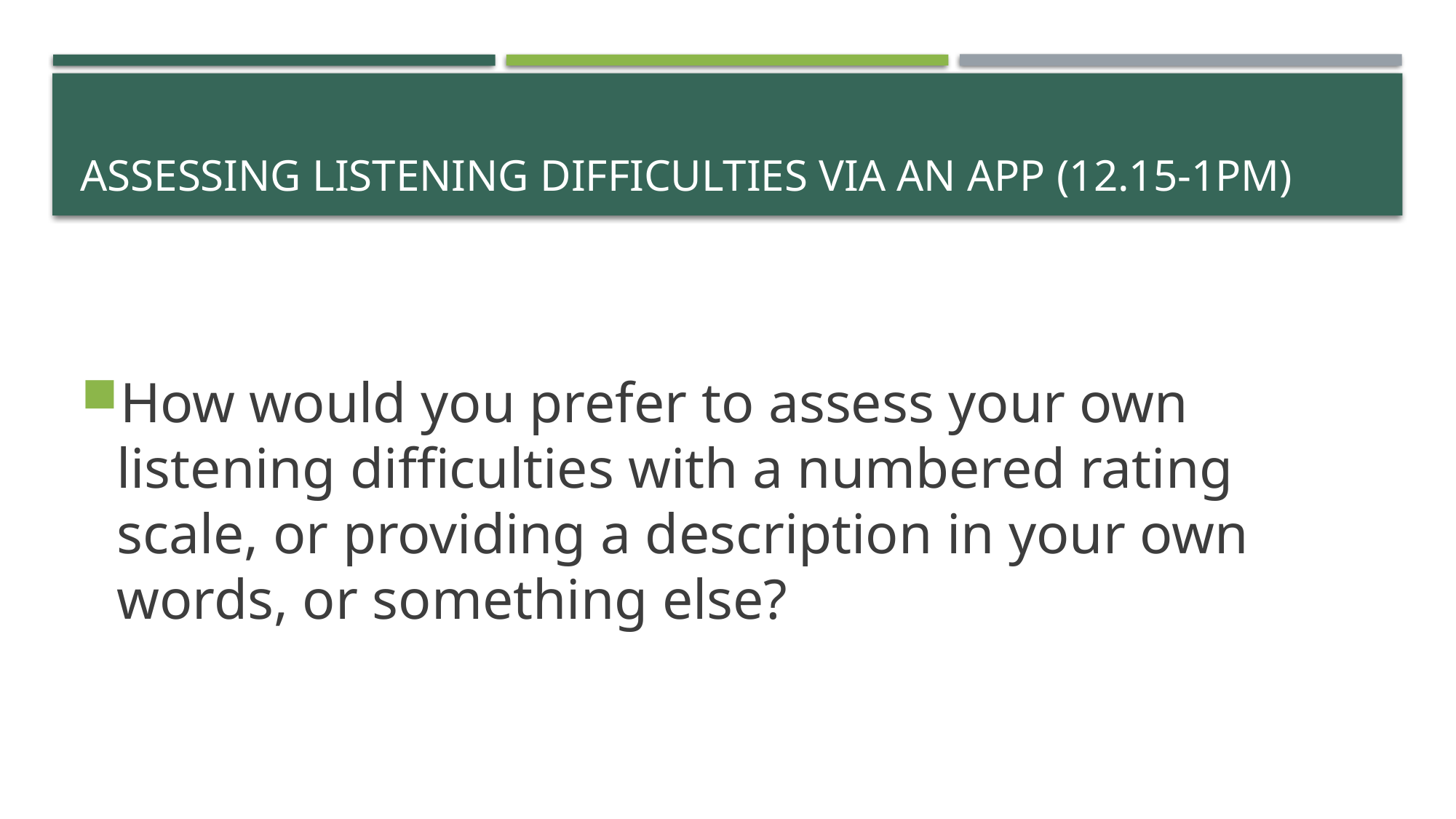

# Assessing listening difficulties via an app (12.15-1pm)
How would you prefer to assess your own listening difficulties with a numbered rating scale, or providing a description in your own words, or something else?

## Slide 18
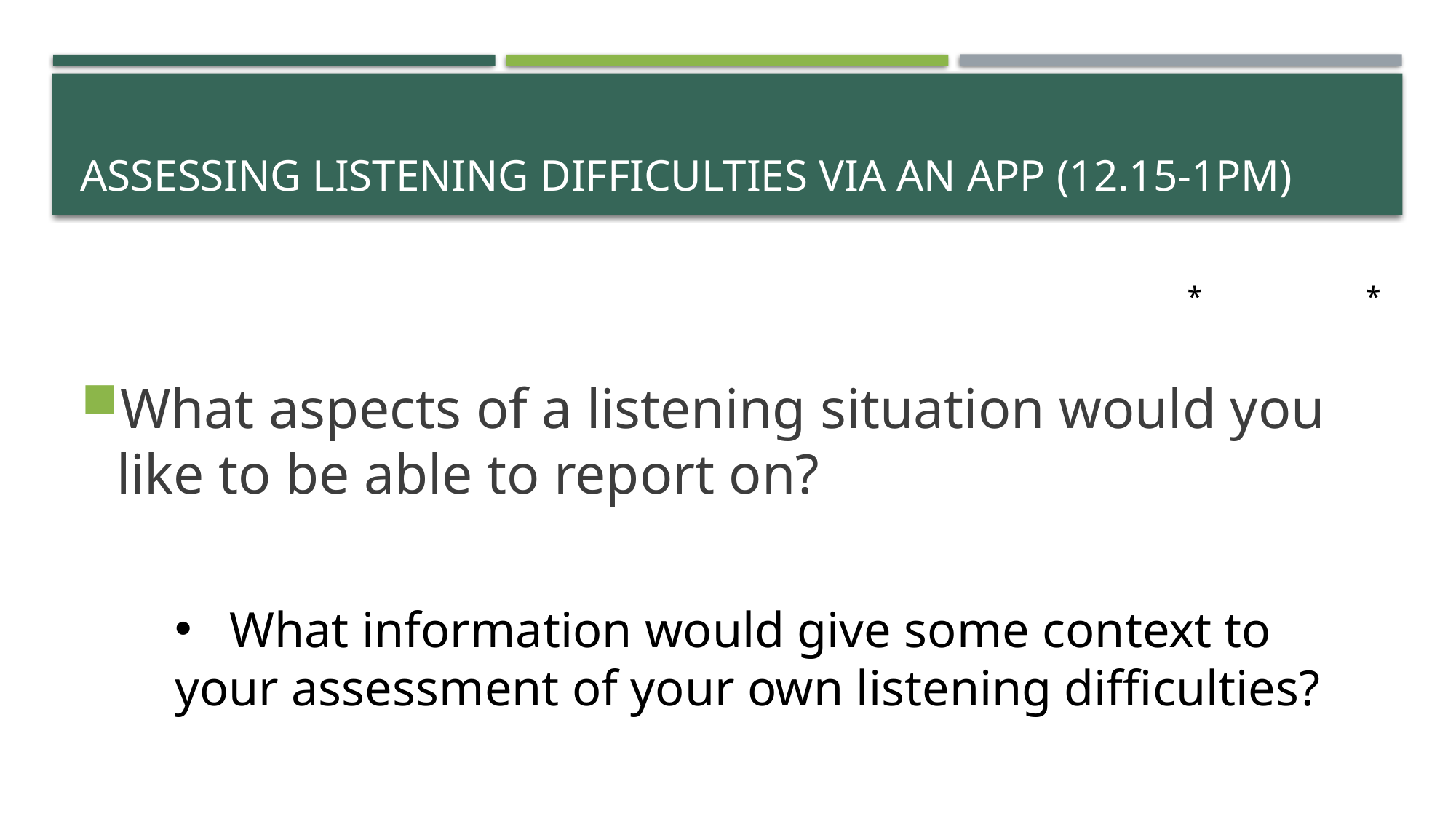

# Assessing listening difficulties via an app (12.15-1pm)
What aspects of a listening situation would you like to be able to report on?
*
*
What information would give some context to
your assessment of your own listening difficulties?

## Slide 19
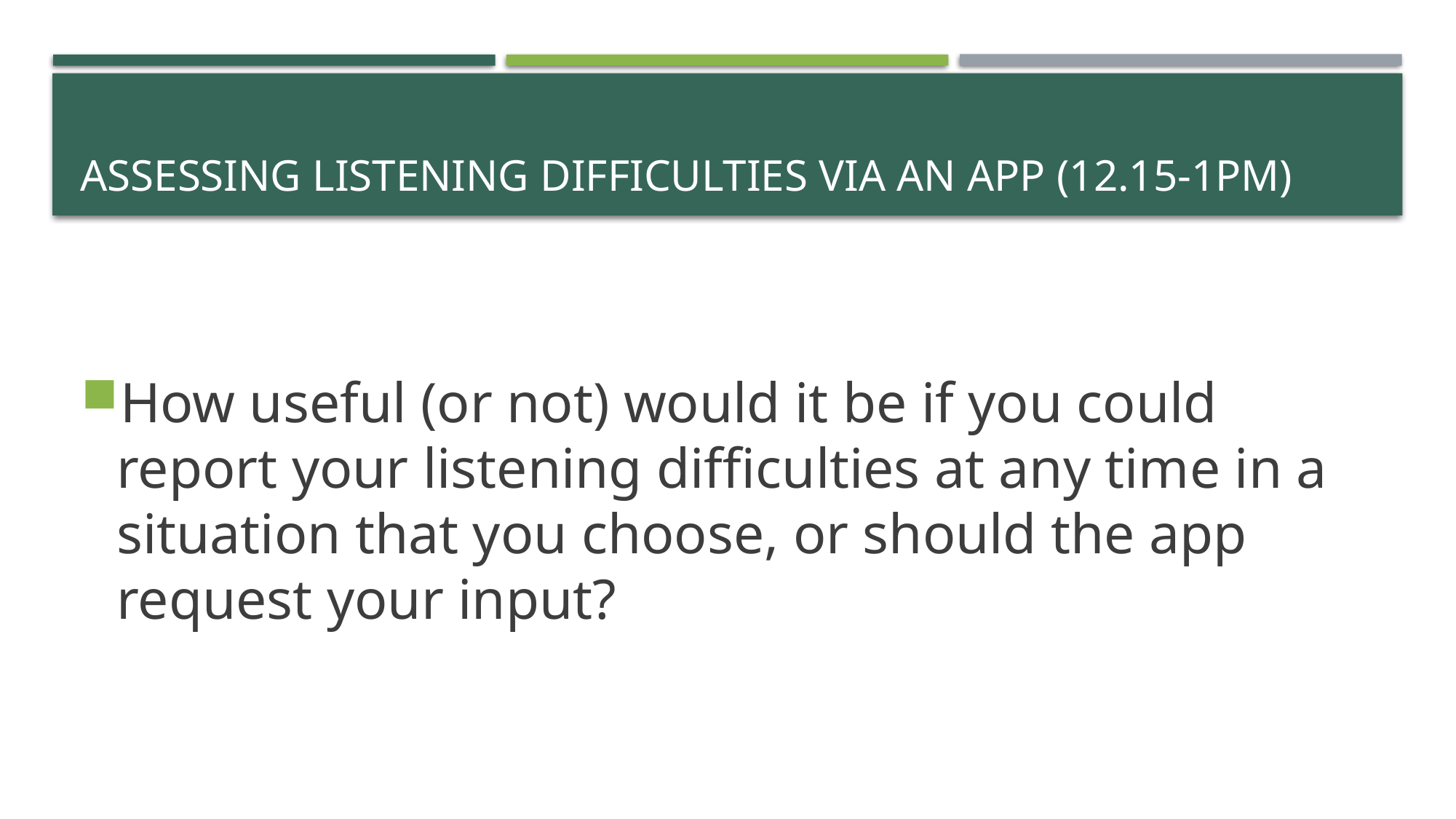

# Assessing listening difficulties via an app (12.15-1pm)
How useful (or not) would it be if you could report your listening difficulties at any time in a situation that you choose, or should the app request your input?

## Slide 20
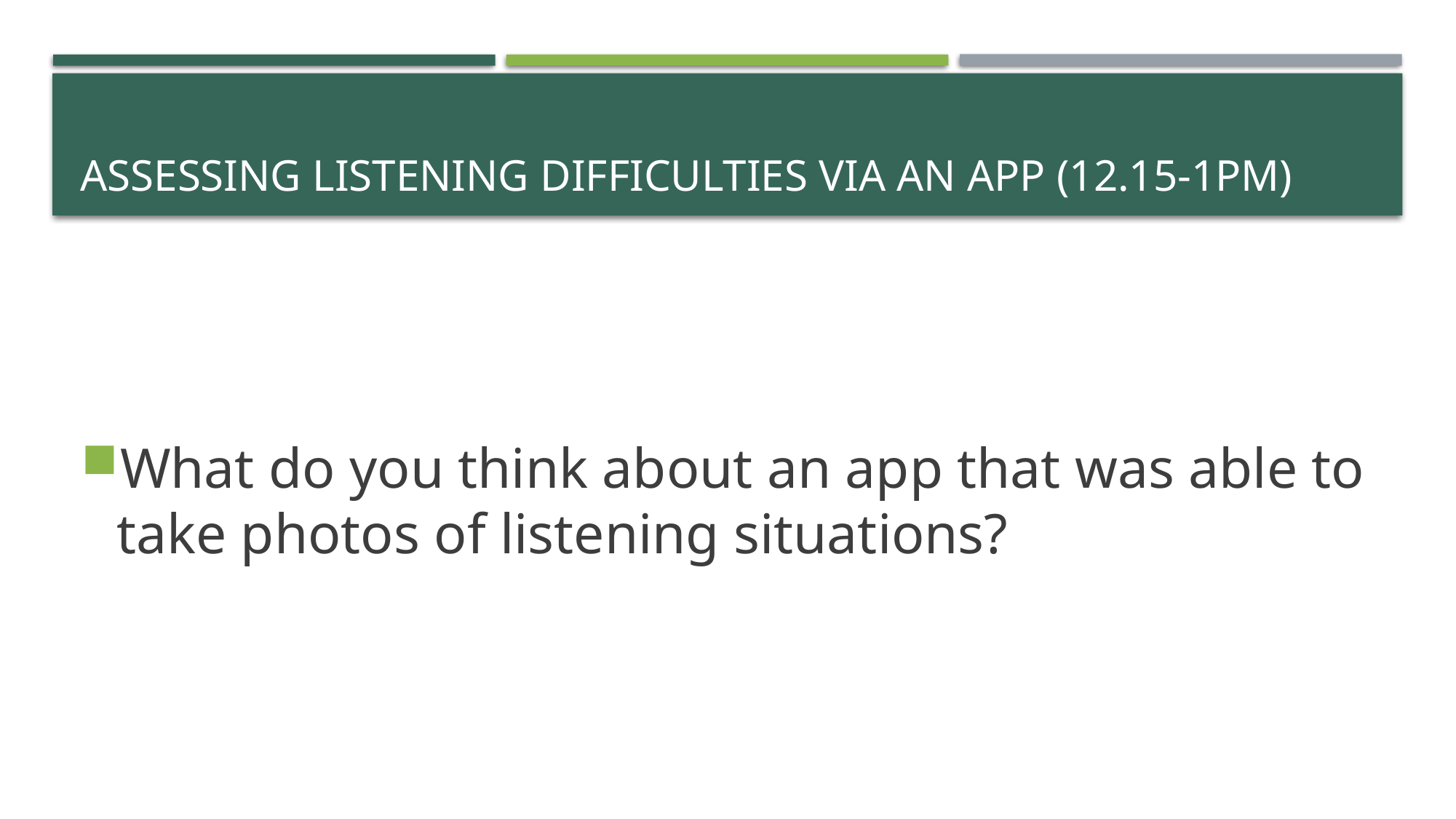

# Assessing listening difficulties via an app (12.15-1pm)
What do you think about an app that was able to take photos of listening situations?

## Slide 21
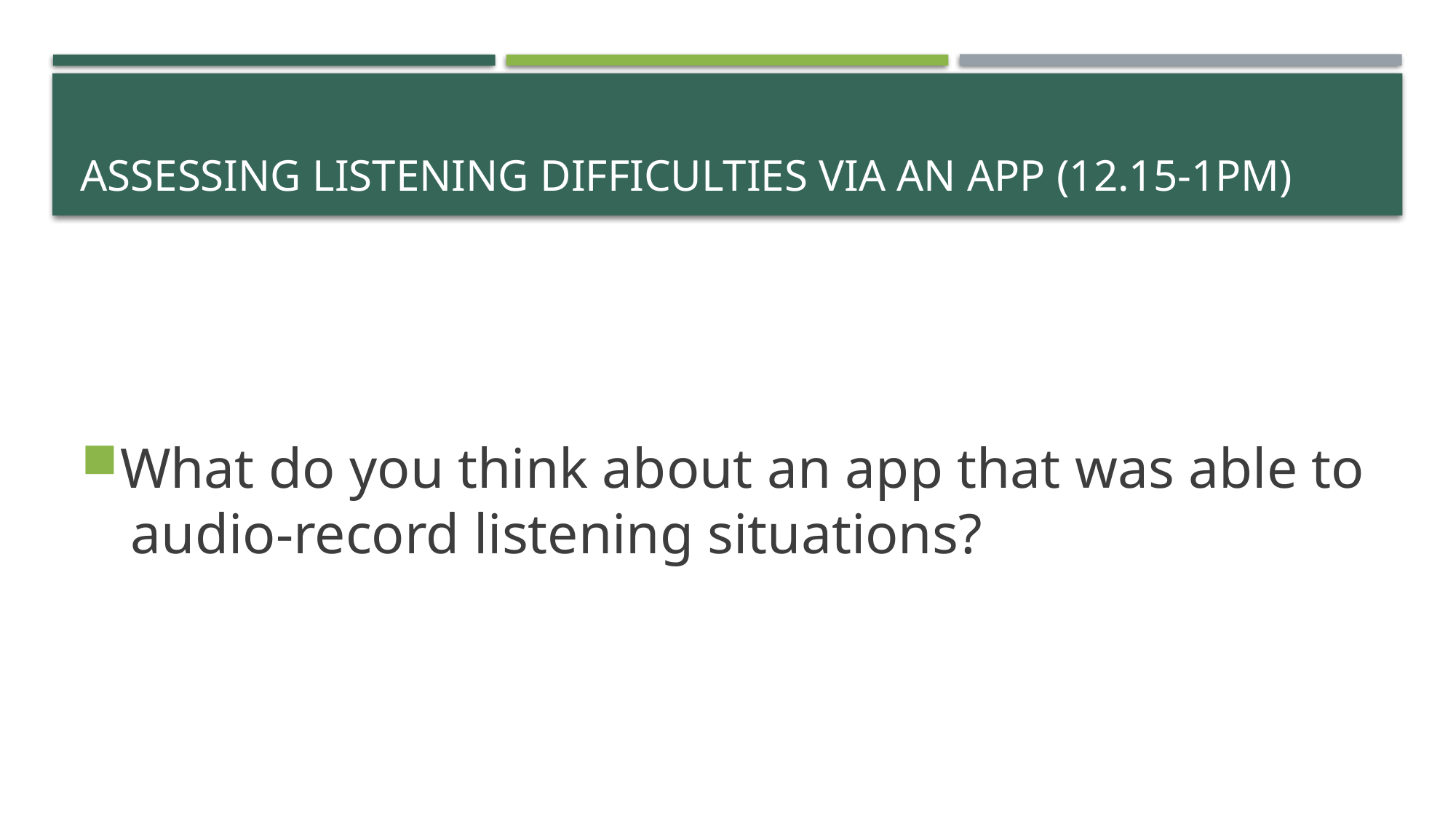

# Assessing listening difficulties via an app (12.15-1pm)
What do you think about an app that was able to audio-record listening situations?

## Slide 22
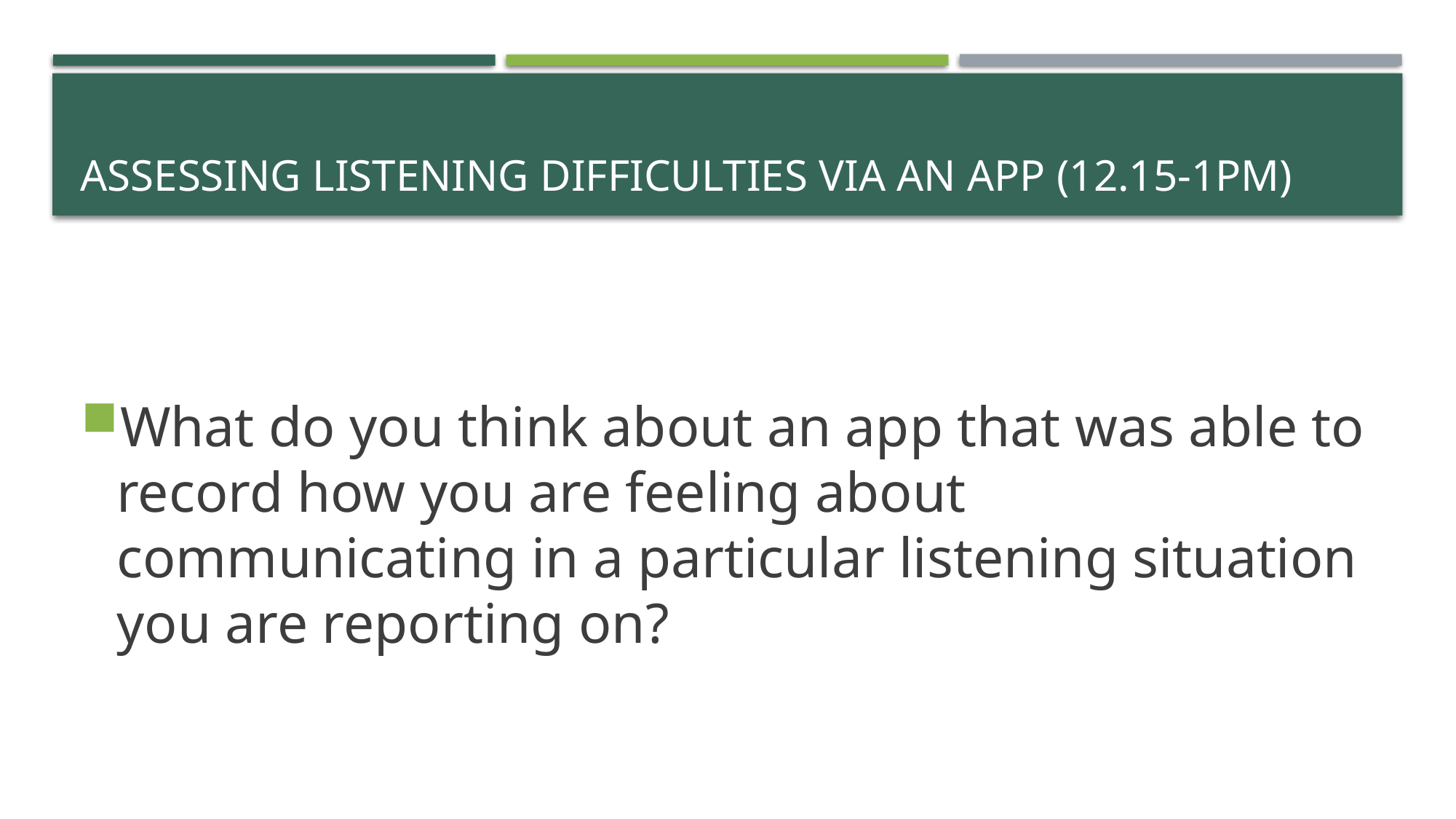

# Assessing listening difficulties via an app (12.15-1pm)
What do you think about an app that was able to record how you are feeling about communicating in a particular listening situation you are reporting on?

## Slide 23
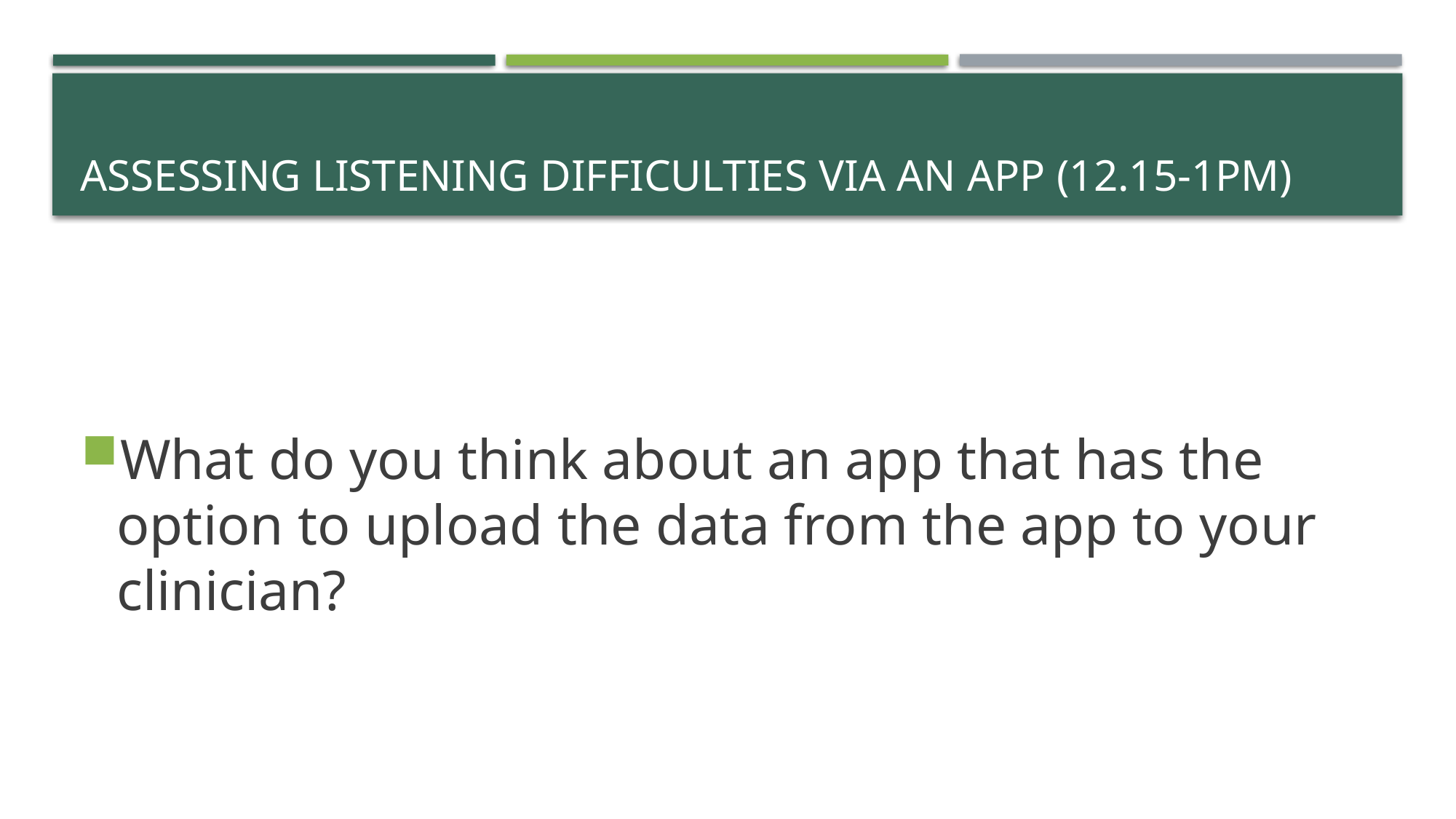

# Assessing listening difficulties via an app (12.15-1pm)
What do you think about an app that has the option to upload the data from the app to your clinician?

## Slide 24
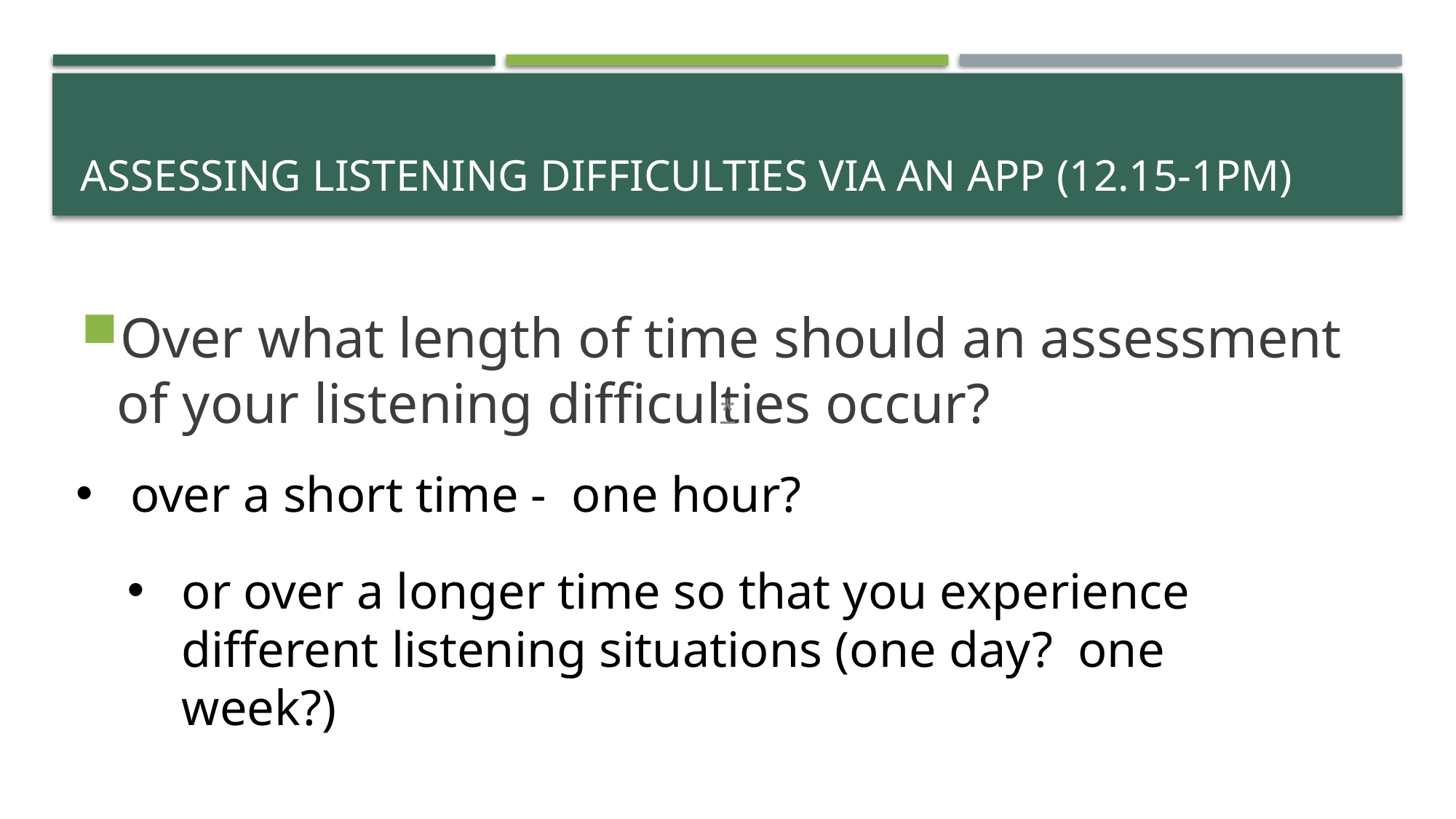

# Assessing listening difficulties via an app (12.15-1pm)
Over what length of time should an assessment of your listening difficulties occur?
*
over a short time - one hour?
or over a longer time so that you experience different listening situations (one day? one week?)

## Slide 25
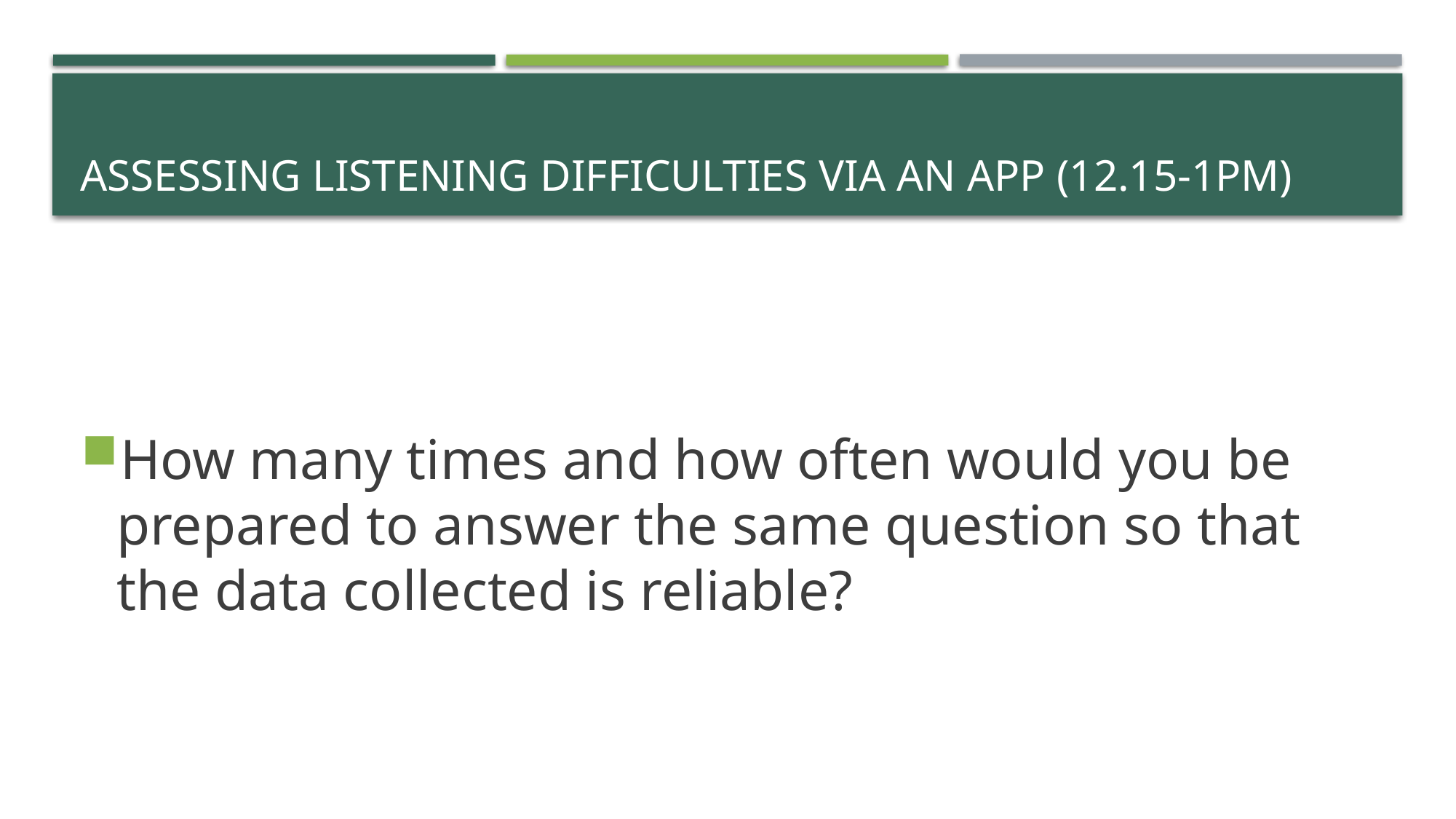

# Assessing listening difficulties via an app (12.15-1pm)
How many times and how often would you be prepared to answer the same question so that the data collected is reliable?

## Slide 26
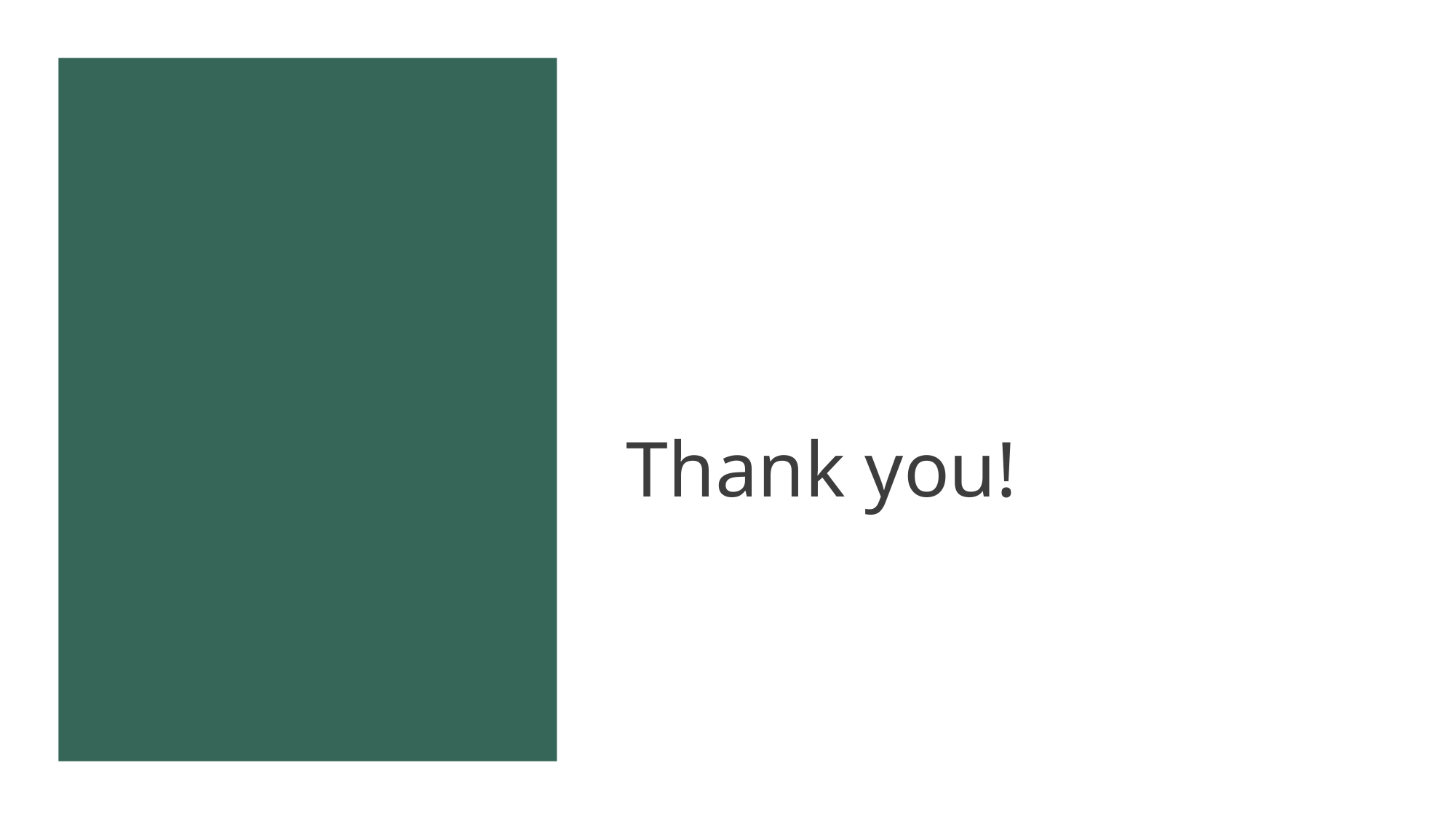

Thank you!
