# Supplementary material for: Consumer Perspectives for a Future Mobile App to Document Real-World Listening Difficulties: Qualitative Study
Source: JMIR Form Res. 2024 Jul 23;8:e47578. doi: 10.2196/47578 (PMC11303898; doi:10.2196/47578)
Supplement: Multimedia Appendix 4 [file formative_v8i1e47578_app4.pptx]

## Slide 1
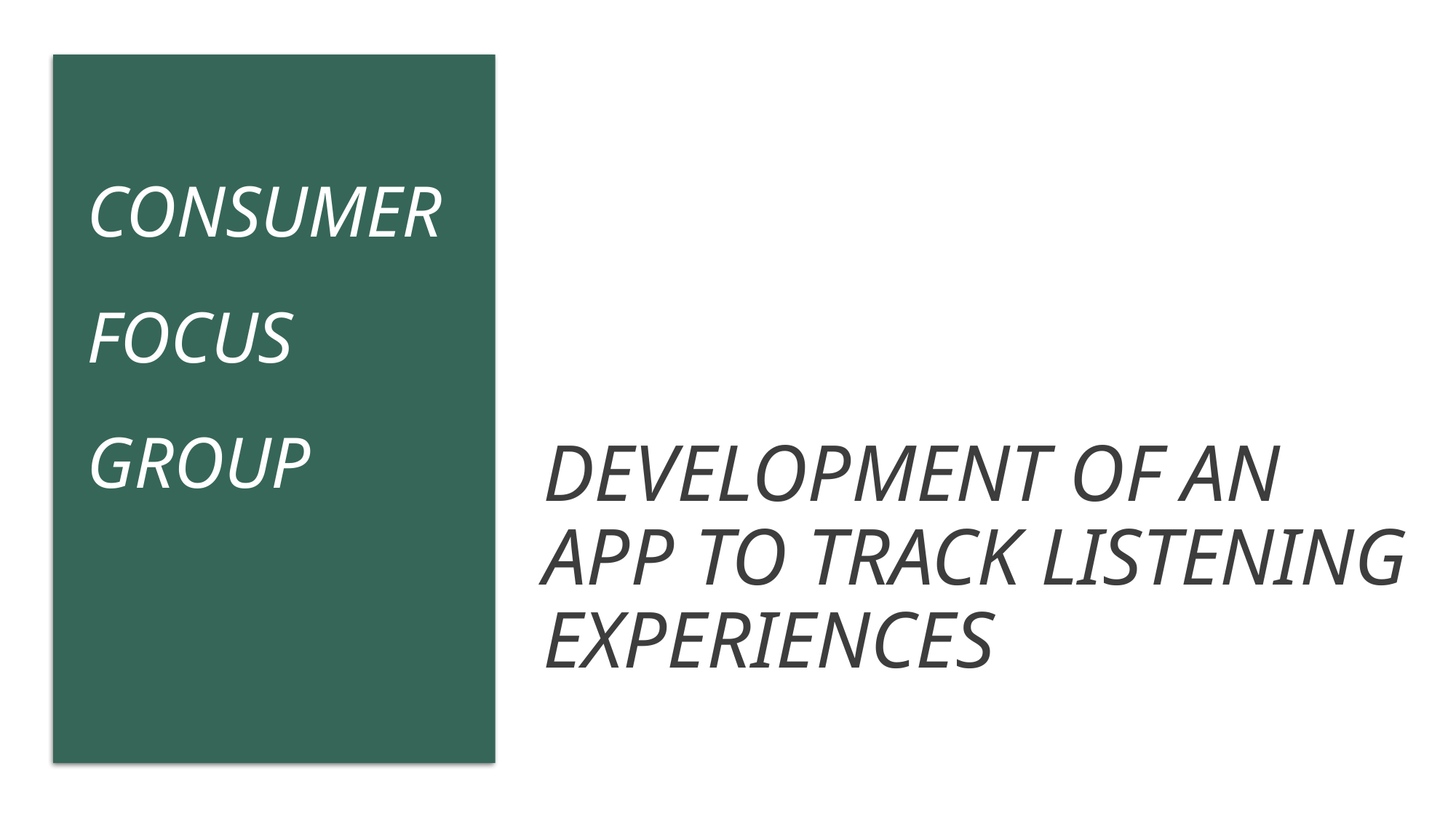

CONSUMER FOCUS GROUP
# development of an app to track listening experiences

## Slide 2
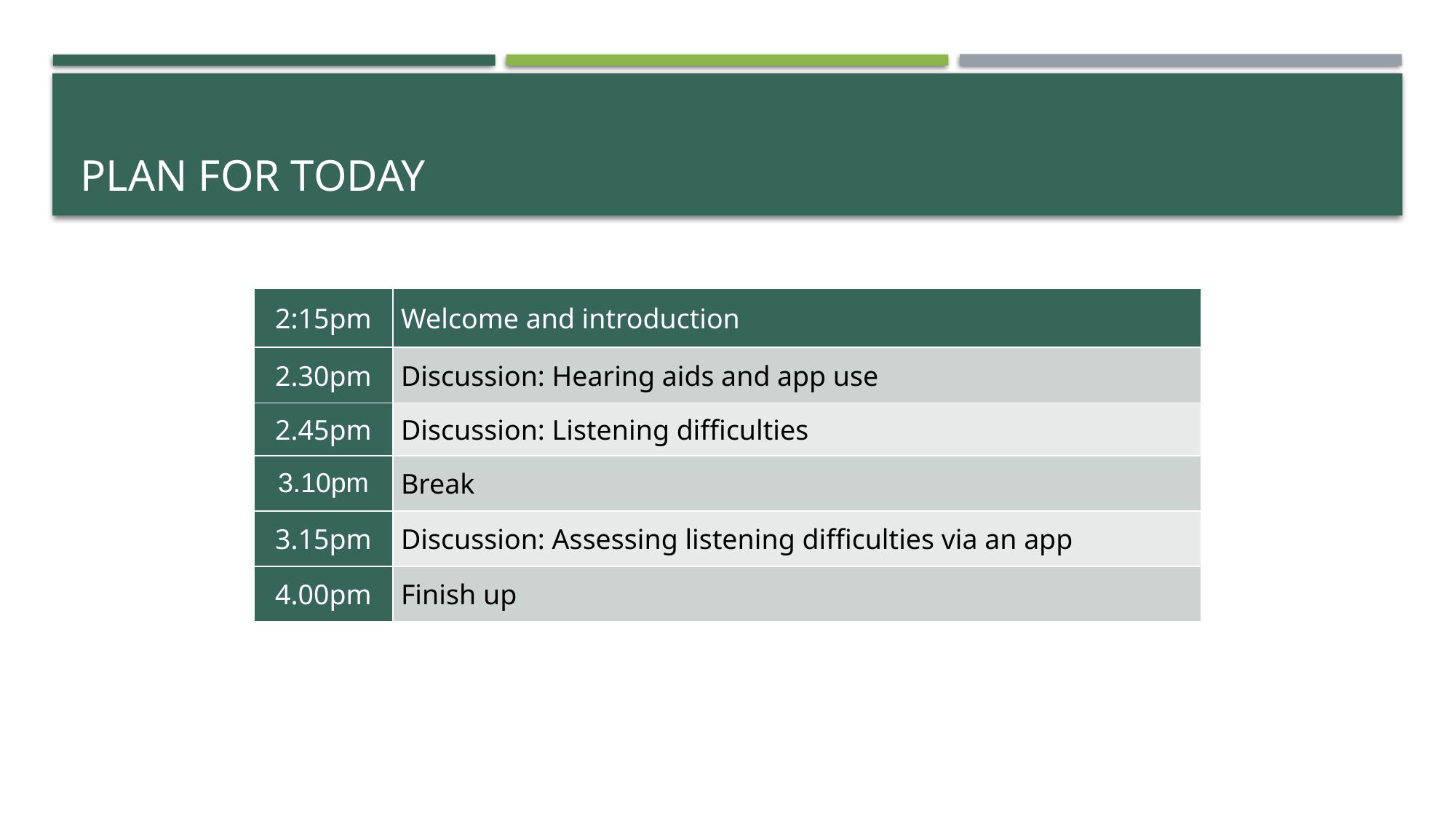

# Plan for today
| 2:15pm | Welcome and introduction |
| --- | --- |
| 2.30pm | Discussion: Hearing aids and app use |
| 2.45pm | Discussion: Listening difficulties |
| 3.10pm | Break |
| 3.15pm | Discussion: Assessing listening difficulties via an app |
| 4.00pm | Finish up |

## Slide 3
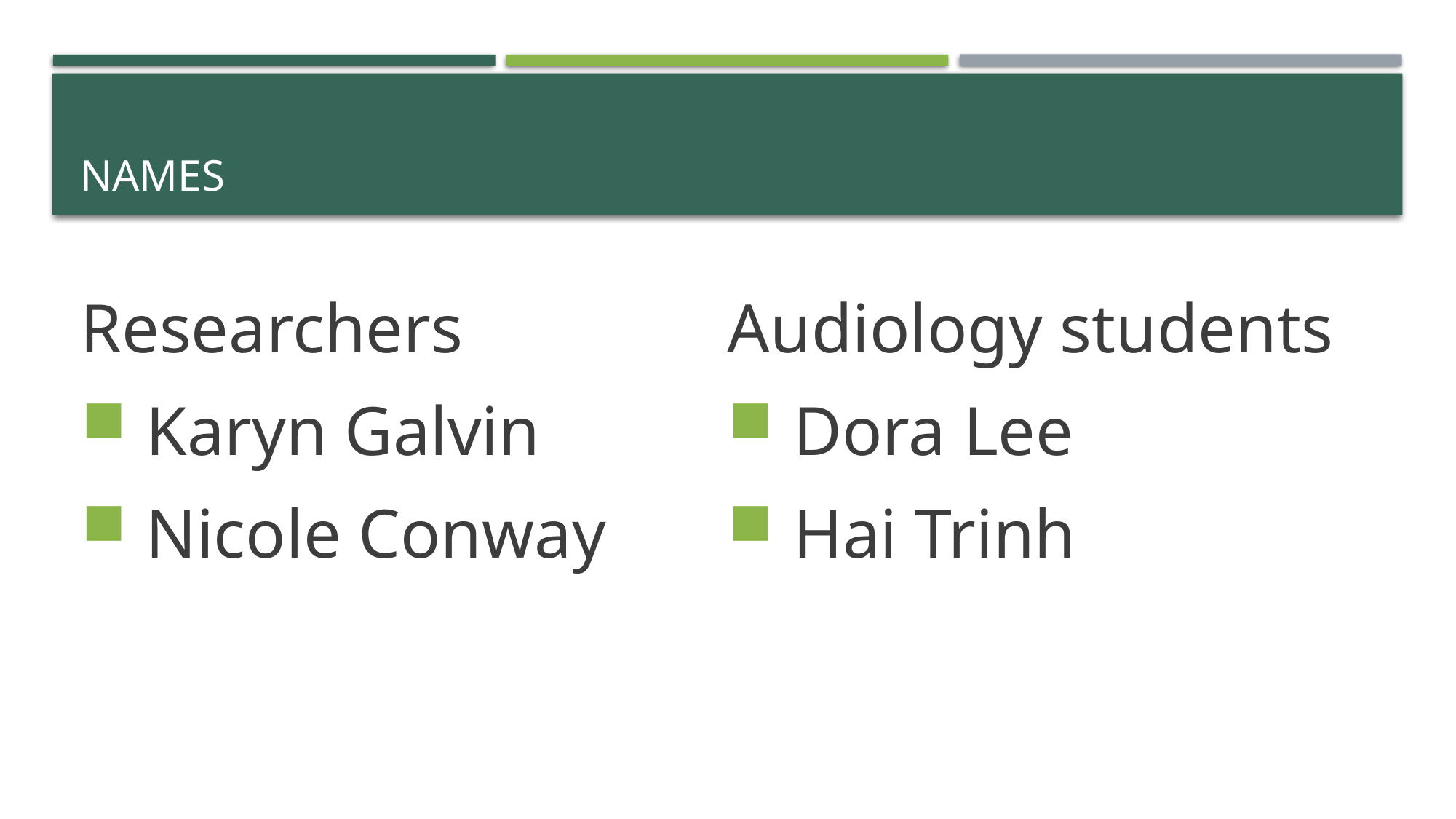

# Names
Researchers
 Karyn Galvin
 Nicole Conway
Audiology students
 Dora Lee
 Hai Trinh

## Slide 4
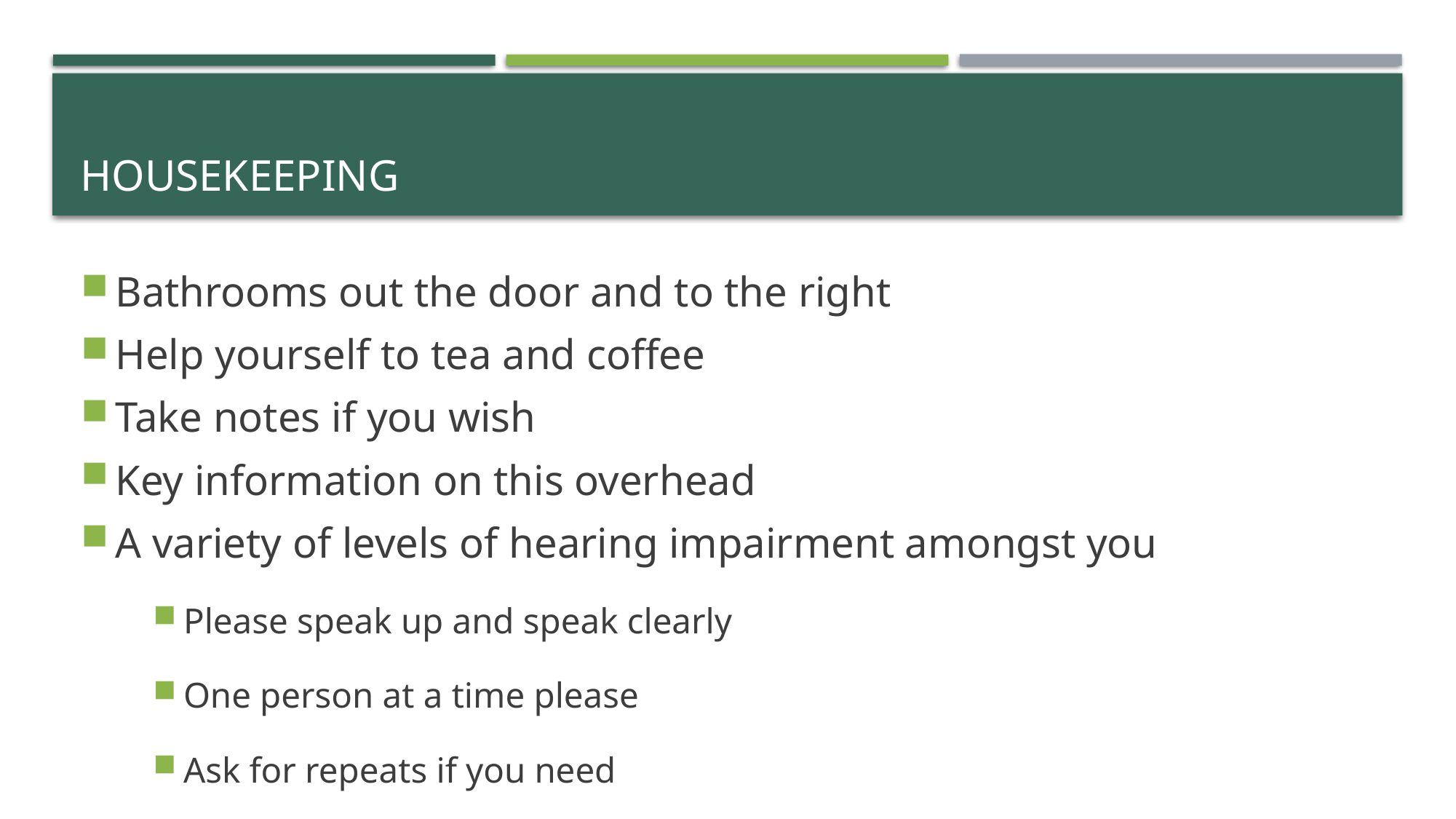

# Housekeeping
Bathrooms out the door and to the right
Help yourself to tea and coffee
Take notes if you wish
Key information on this overhead
A variety of levels of hearing impairment amongst you
Please speak up and speak clearly
One person at a time please
Ask for repeats if you need

## Slide 5
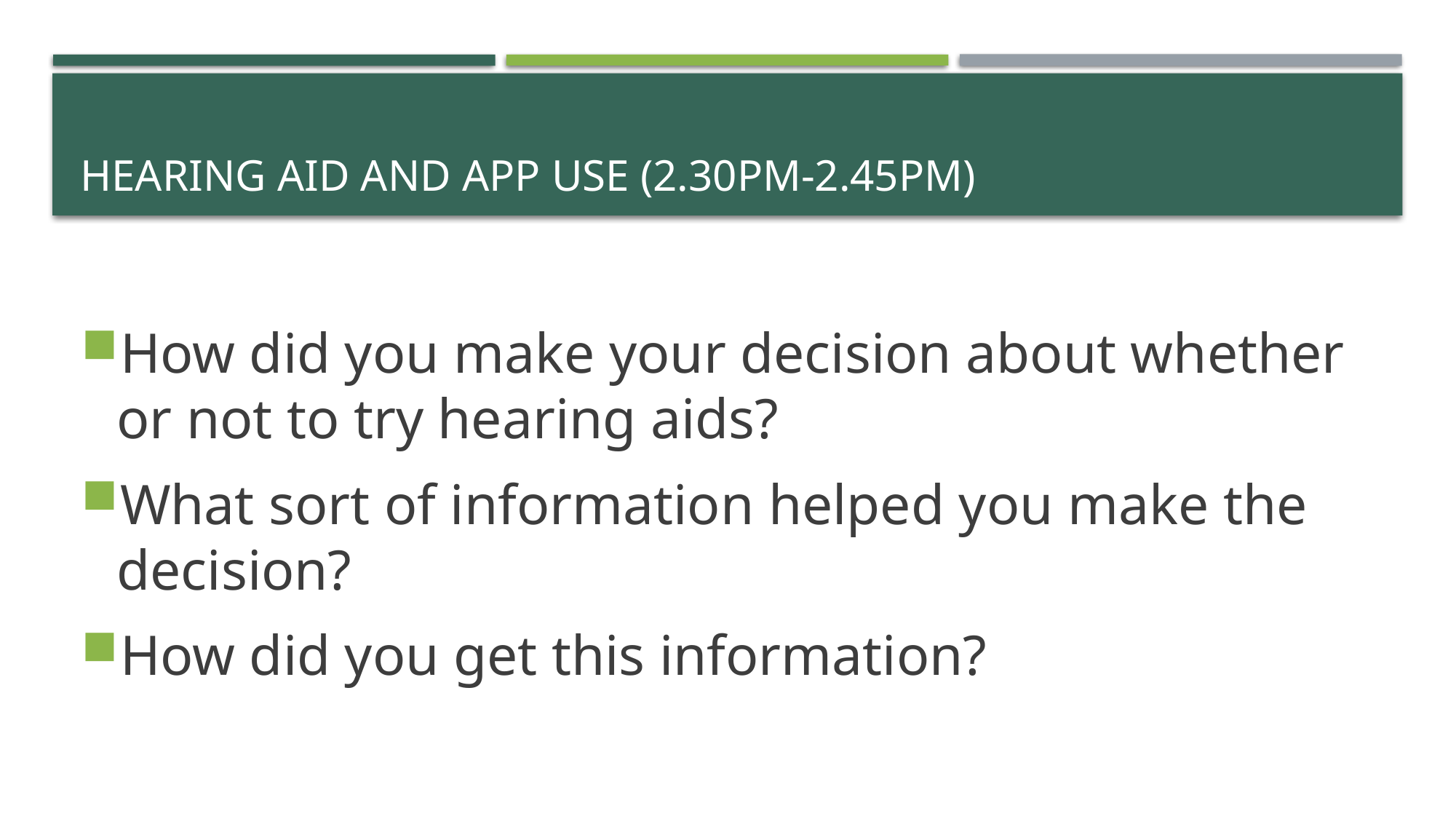

# Hearing aid and app use (2.30pm-2.45pm)
How did you make your decision about whether or not to try hearing aids?
What sort of information helped you make the decision?
How did you get this information?

## Slide 6
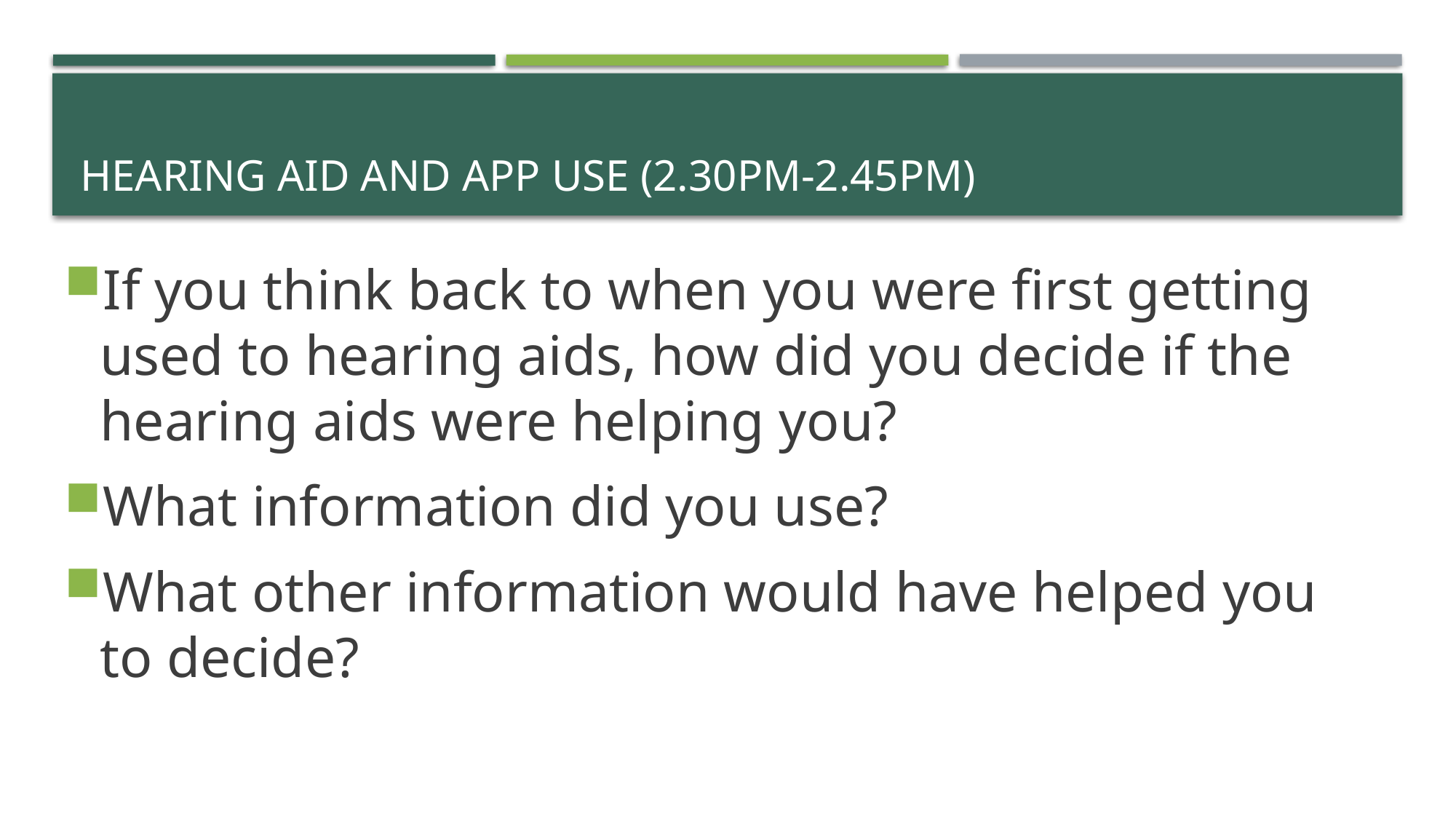

# Hearing aid and app use (2.30pm-2.45pm)
If you think back to when you were first getting used to hearing aids, how did you decide if the hearing aids were helping you?
What information did you use?
What other information would have helped you to decide?

## Slide 7
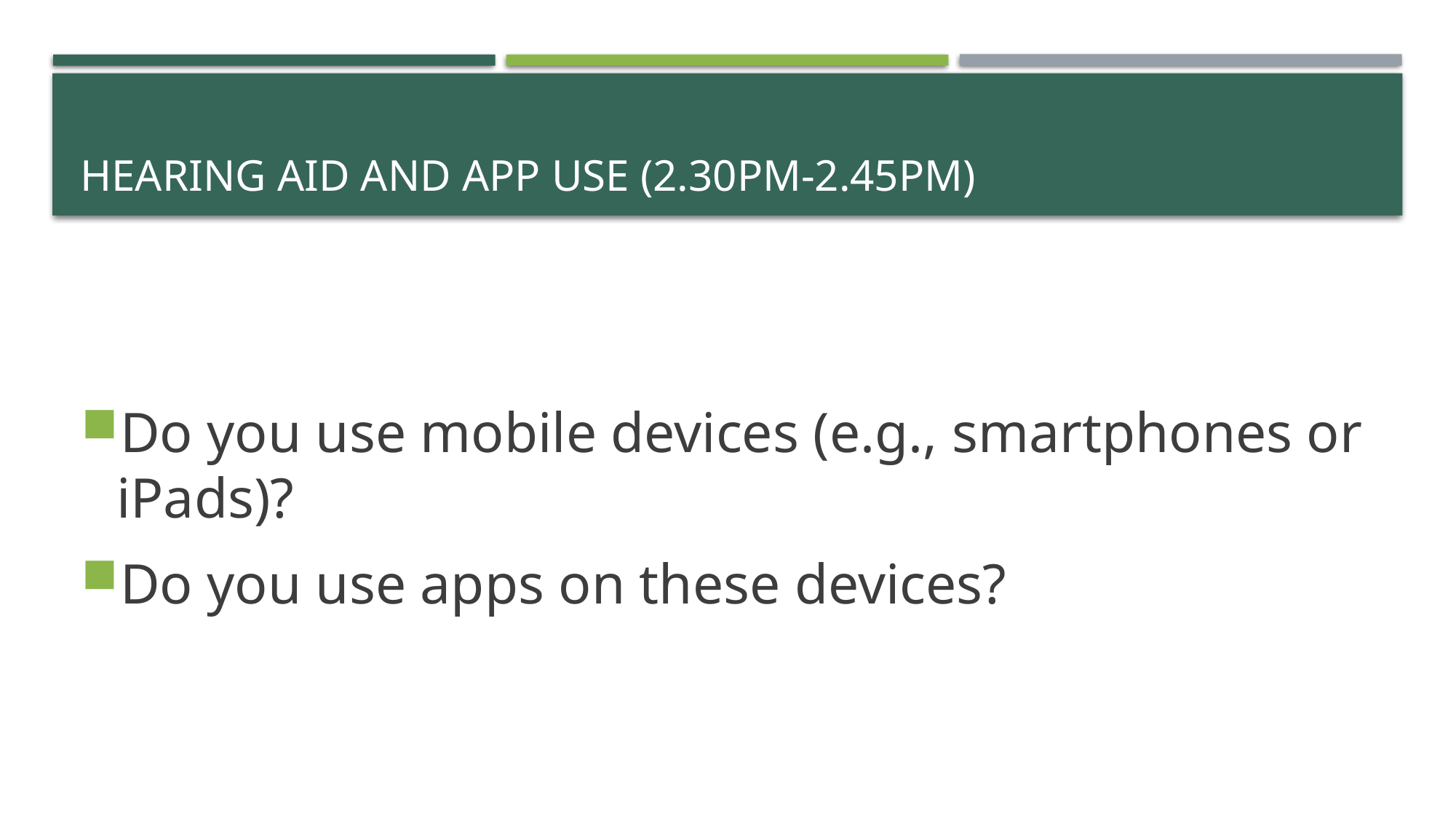

# Hearing aid and app use (2.30pm-2.45pm)
Do you use mobile devices (e.g., smartphones or iPads)?
Do you use apps on these devices?

## Slide 8
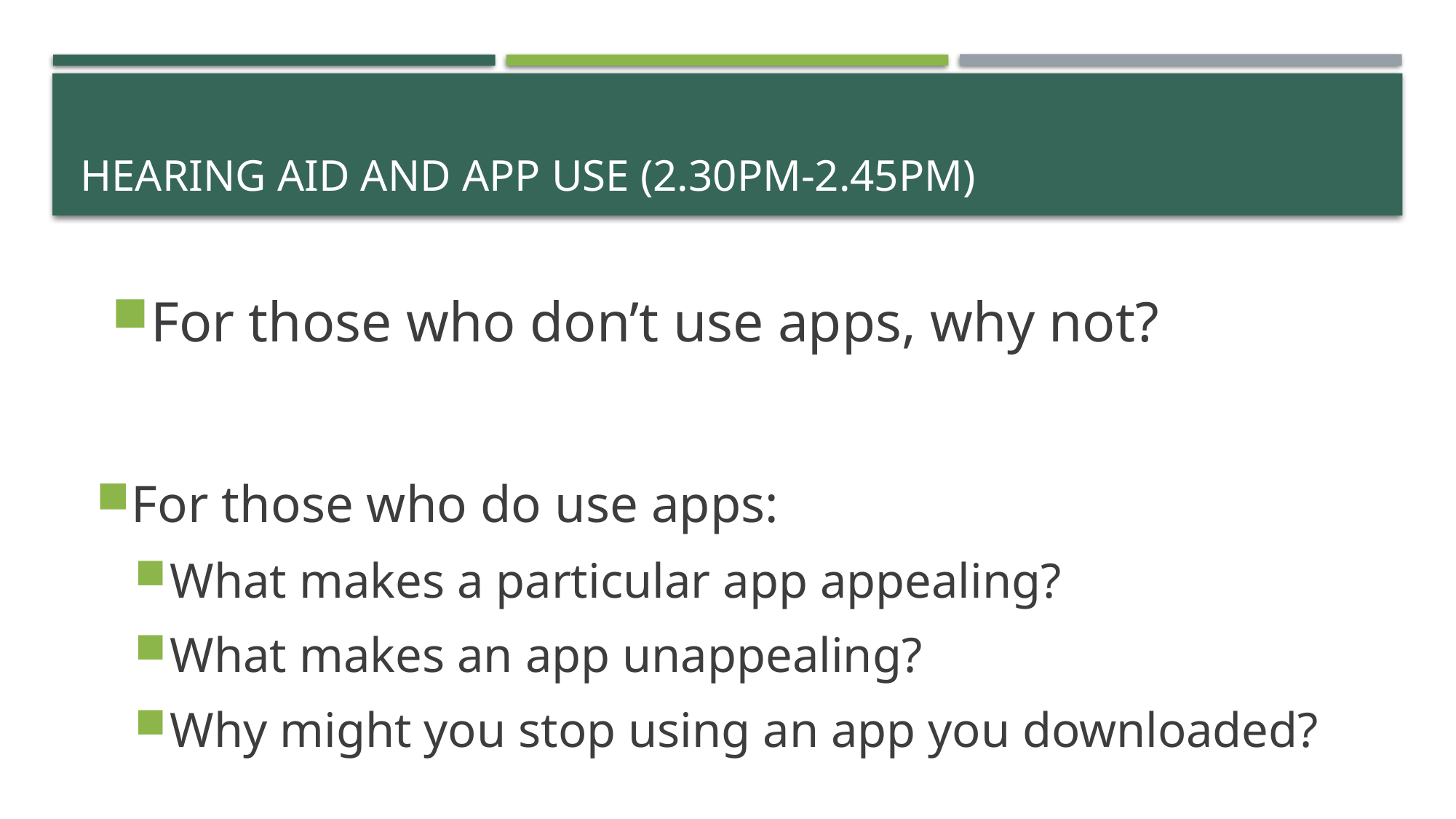

# Hearing aid and app use (2.30pm-2.45pm)
For those who don’t use apps, why not?
For those who do use apps:
What makes a particular app appealing?
What makes an app unappealing?
Why might you stop using an app you downloaded?

## Slide 9
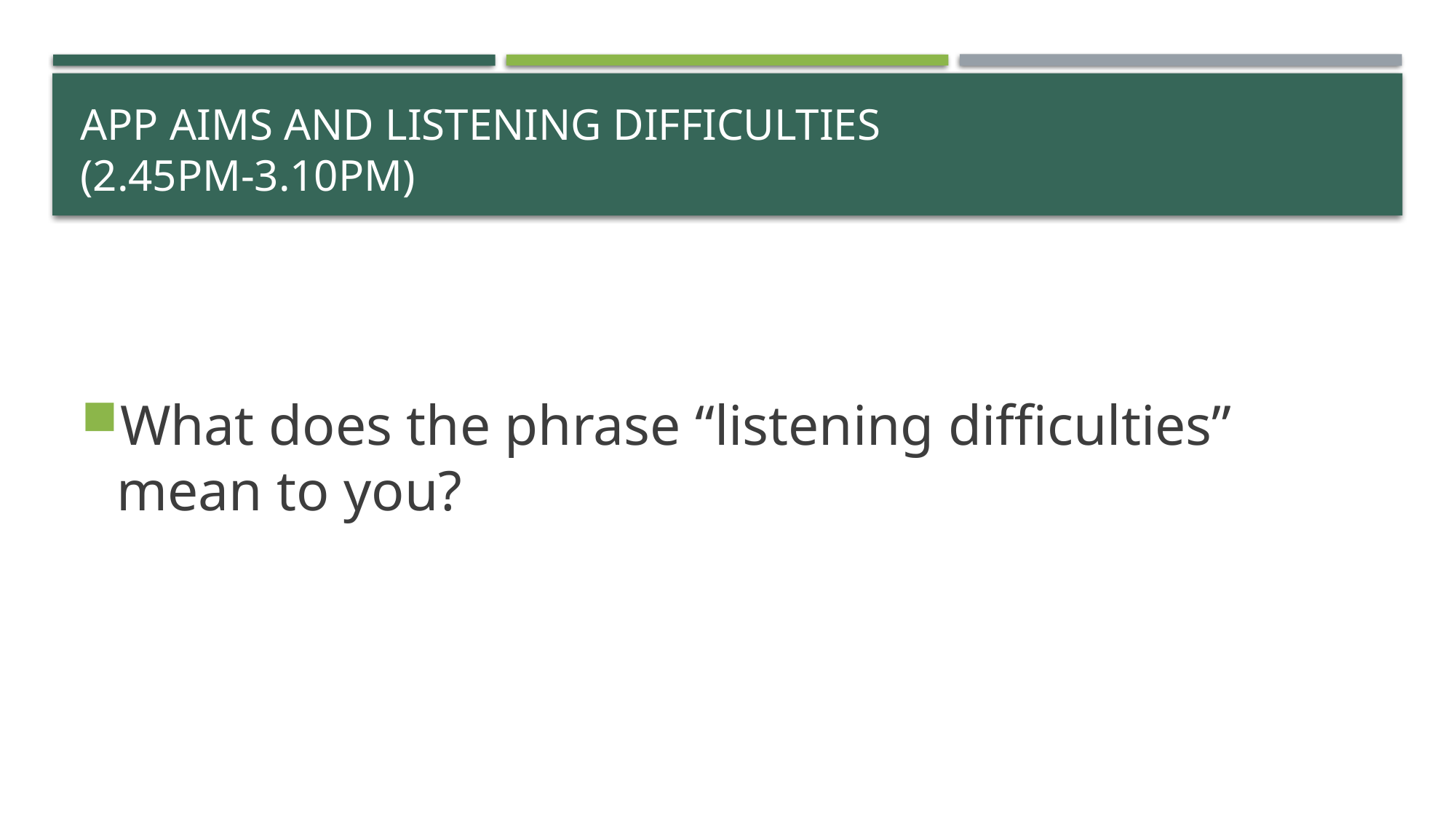

# APP AIMS AND listening difficulties (2.45pm-3.10pm)
What does the phrase “listening difficulties” mean to you?

## Slide 10
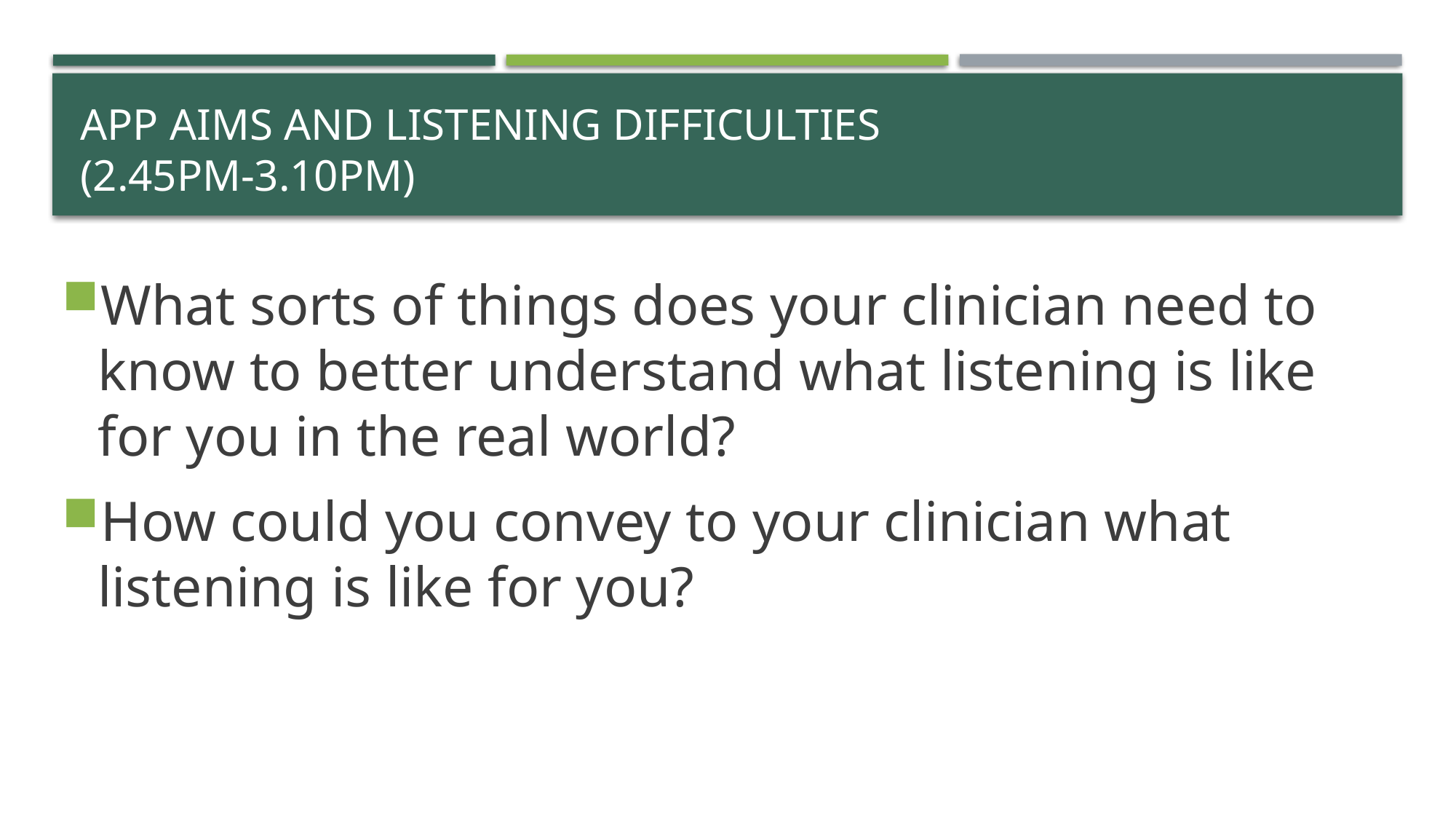

# App aims and listening difficulties (2.45pm-3.10pm)
What sorts of things does your clinician need to know to better understand what listening is like for you in the real world?
How could you convey to your clinician what listening is like for you?

## Slide 11
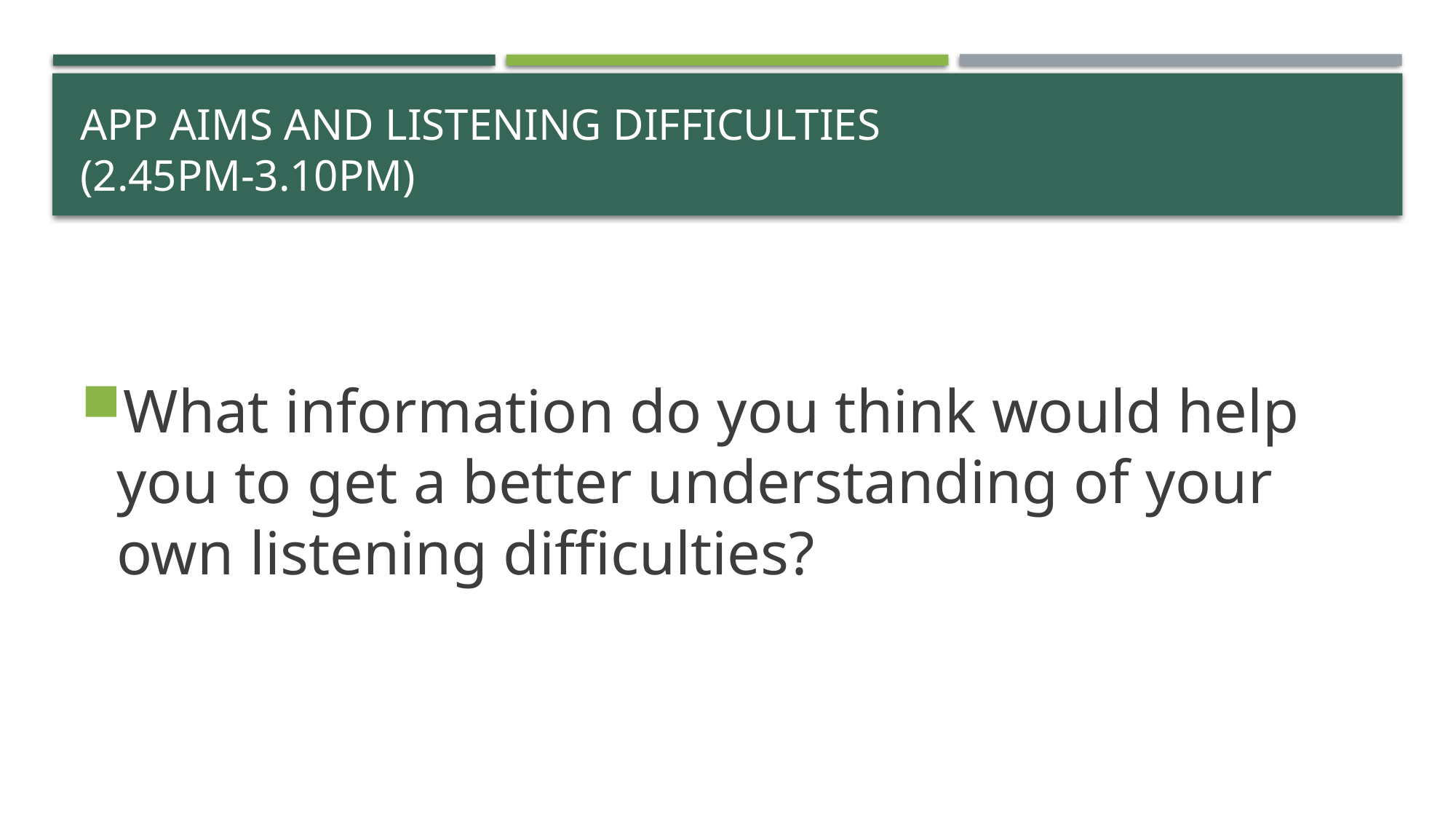

# App aims and listening difficulties (2.45pm-3.10pm)
What information do you think would help you to get a better understanding of your own listening difficulties?

## Slide 12
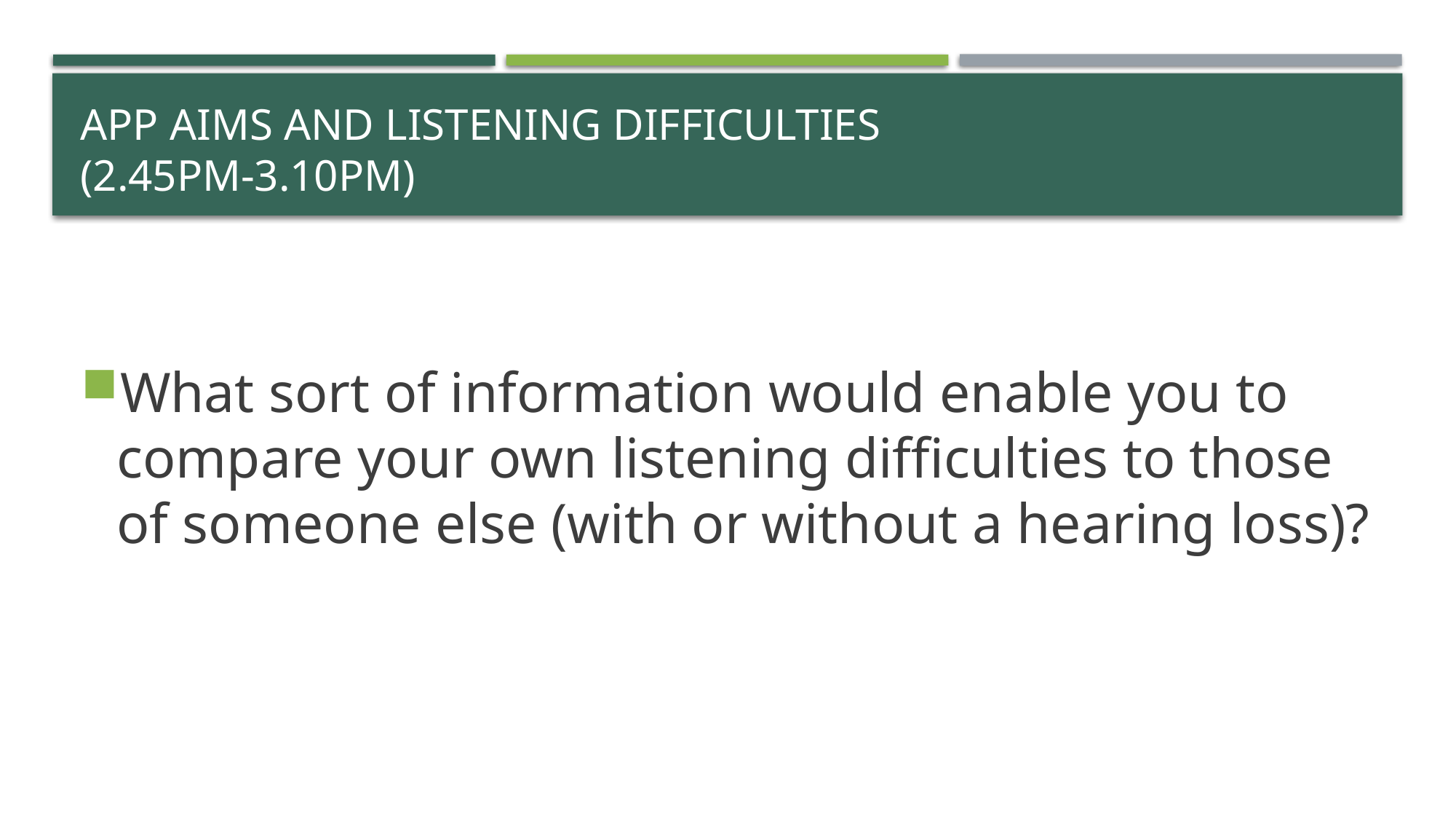

# App aims and listening difficulties (2.45pm-3.10pm)
What sort of information would enable you to compare your own listening difficulties to those of someone else (with or without a hearing loss)?

## Slide 13
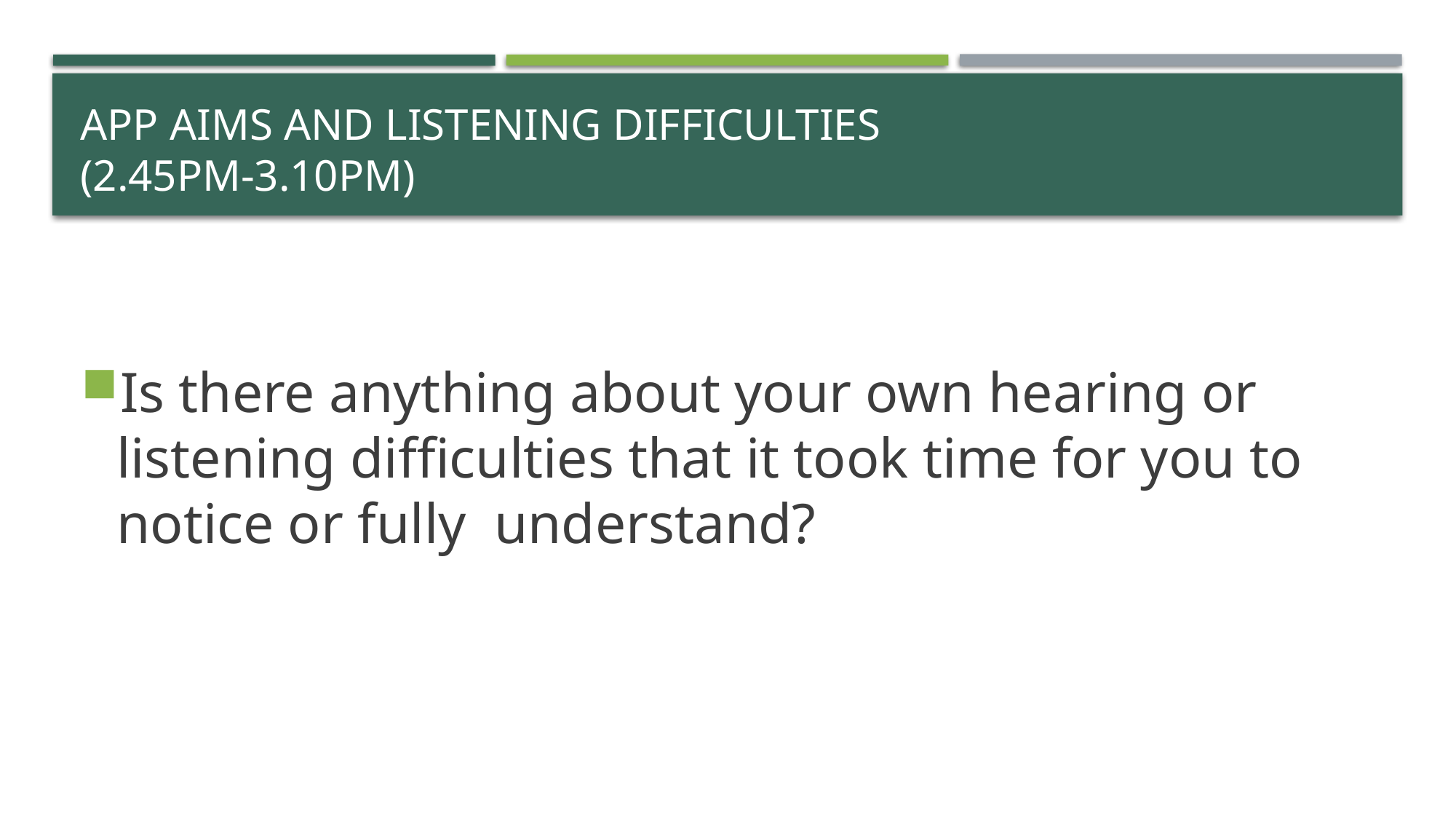

# App aims and listening difficulties (2.45pm-3.10pm)
Is there anything about your own hearing or listening difficulties that it took time for you to notice or fully understand?

## Slide 14
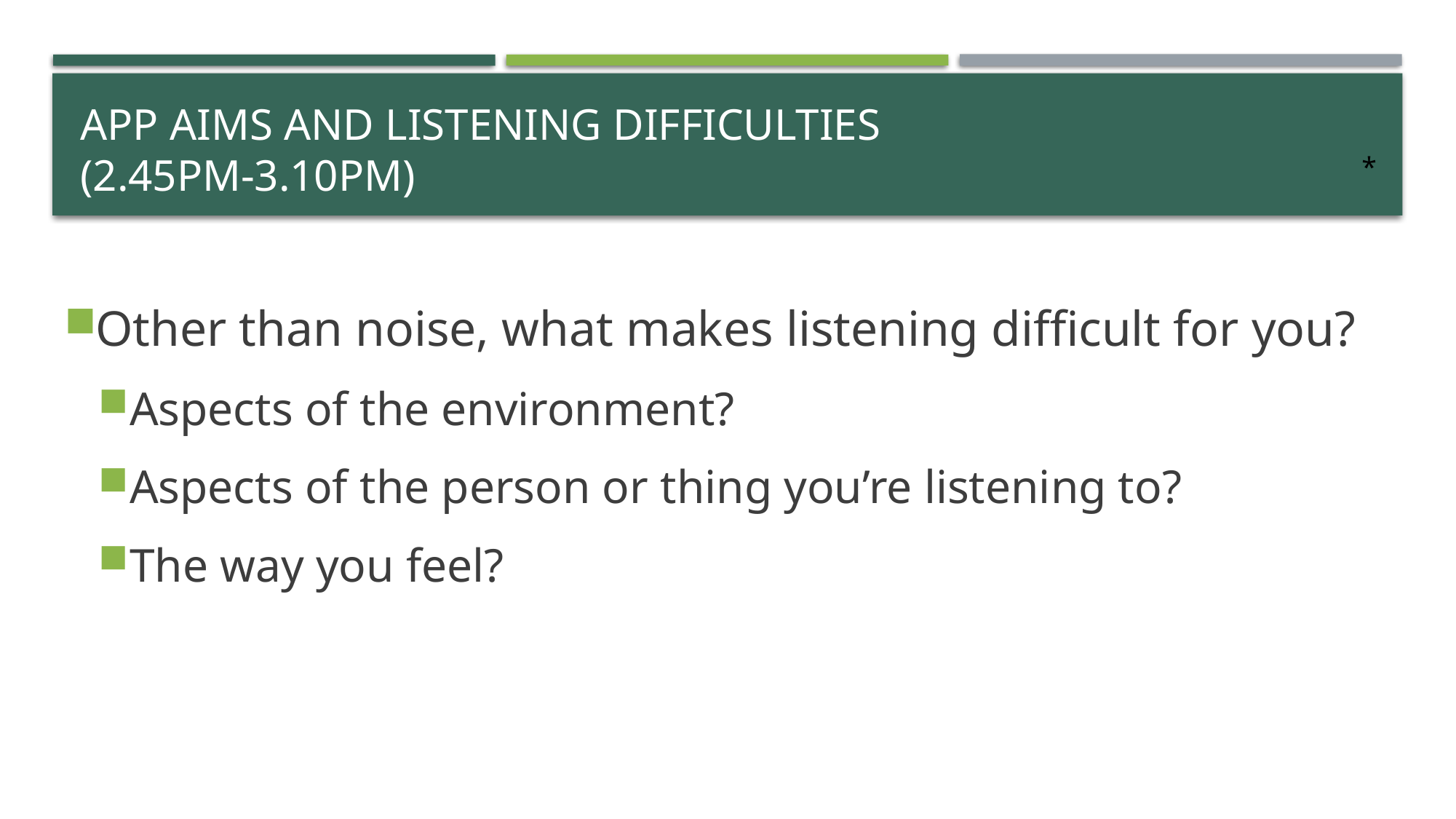

# App aims and listening difficulties (2.45pm-3.10pm)
*
Other than noise, what makes listening difficult for you?
Aspects of the environment?
Aspects of the person or thing you’re listening to?
The way you feel?

## Slide 15
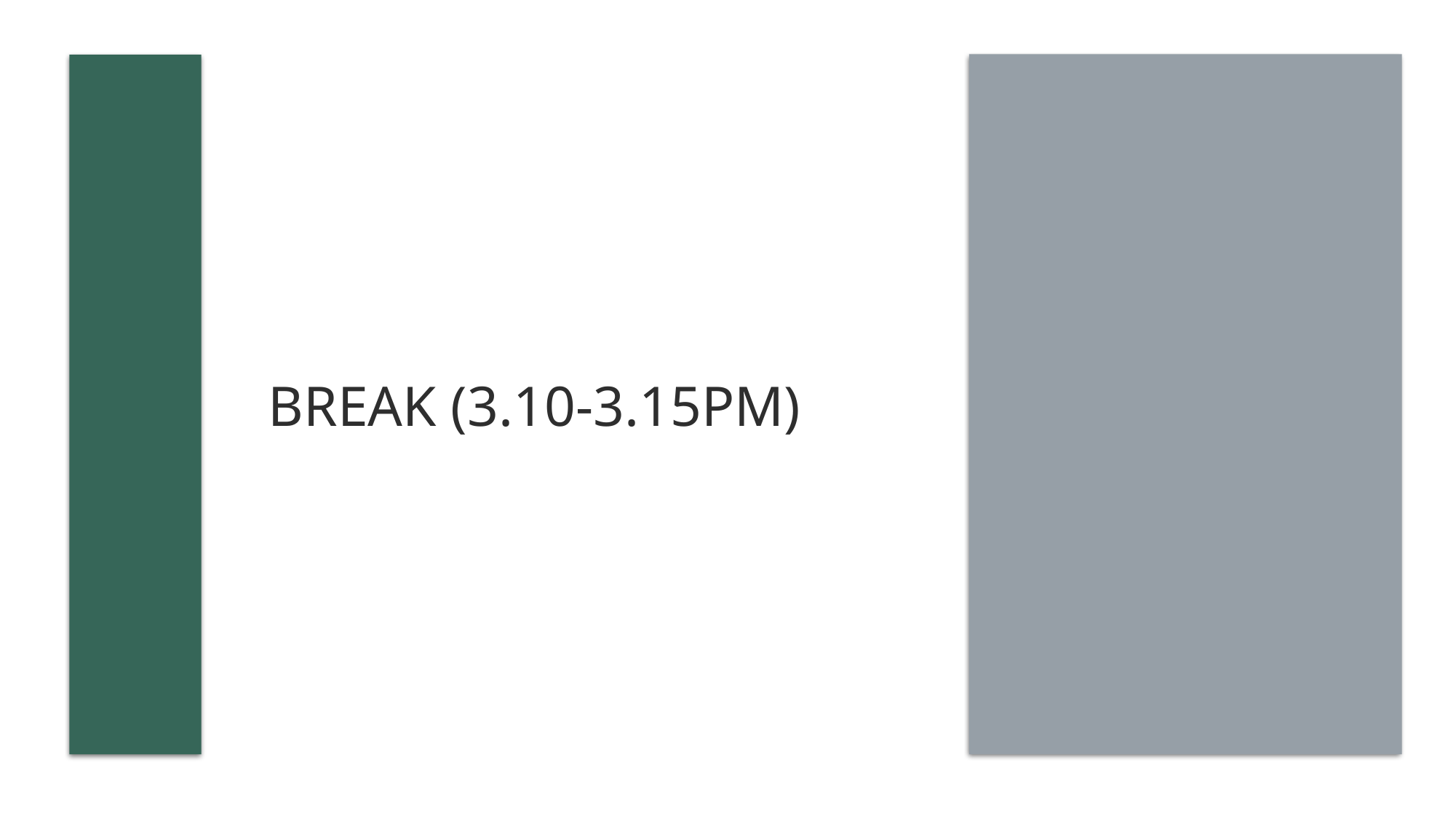

# Break (3.10-3.15pm)

## Slide 16
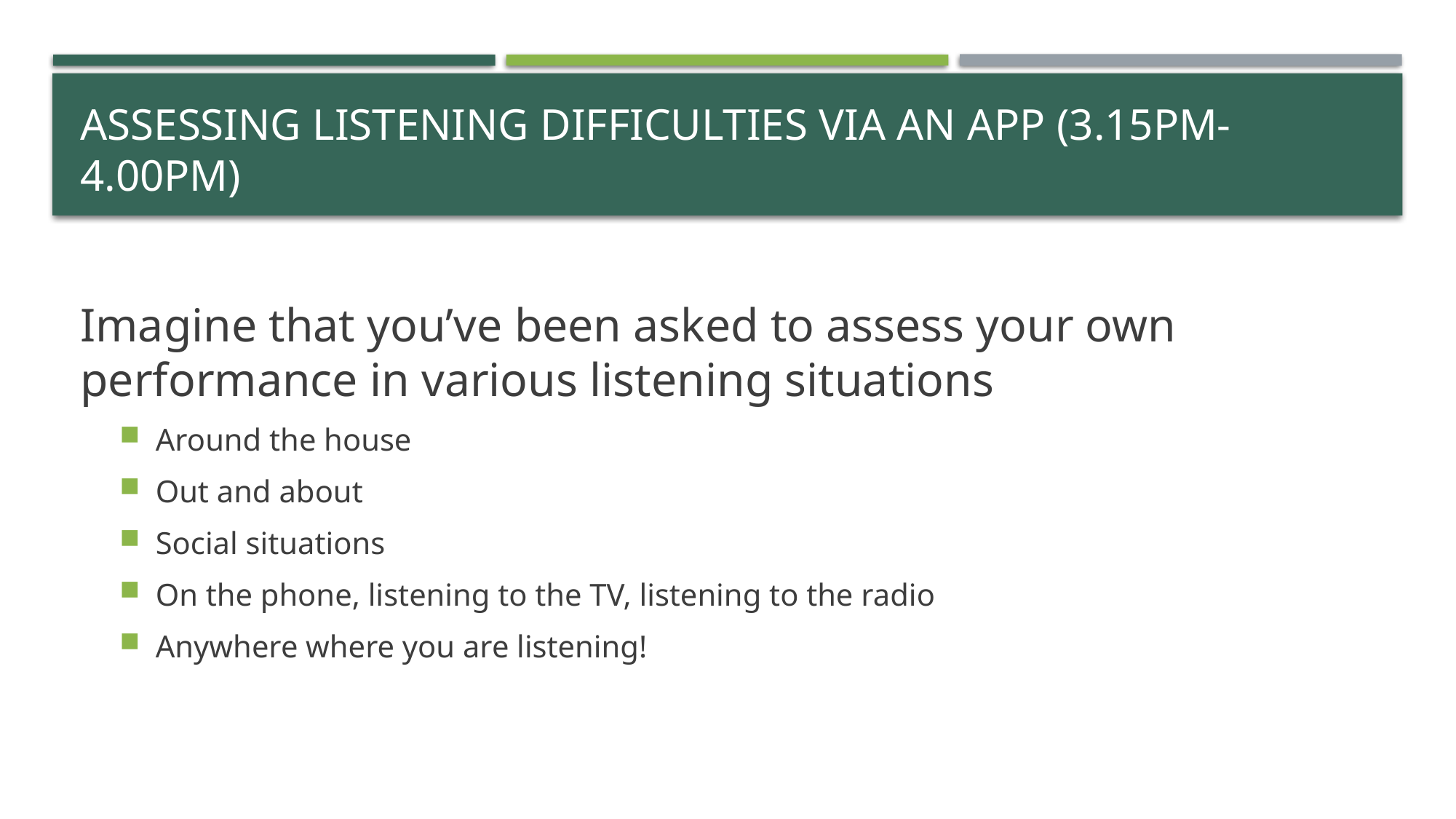

# Assessing listening difficulties via an app (3.15PM-4.00pm)
Imagine that you’ve been asked to assess your own performance in various listening situations
Around the house
Out and about
Social situations
On the phone, listening to the TV, listening to the radio
Anywhere where you are listening!

## Slide 17
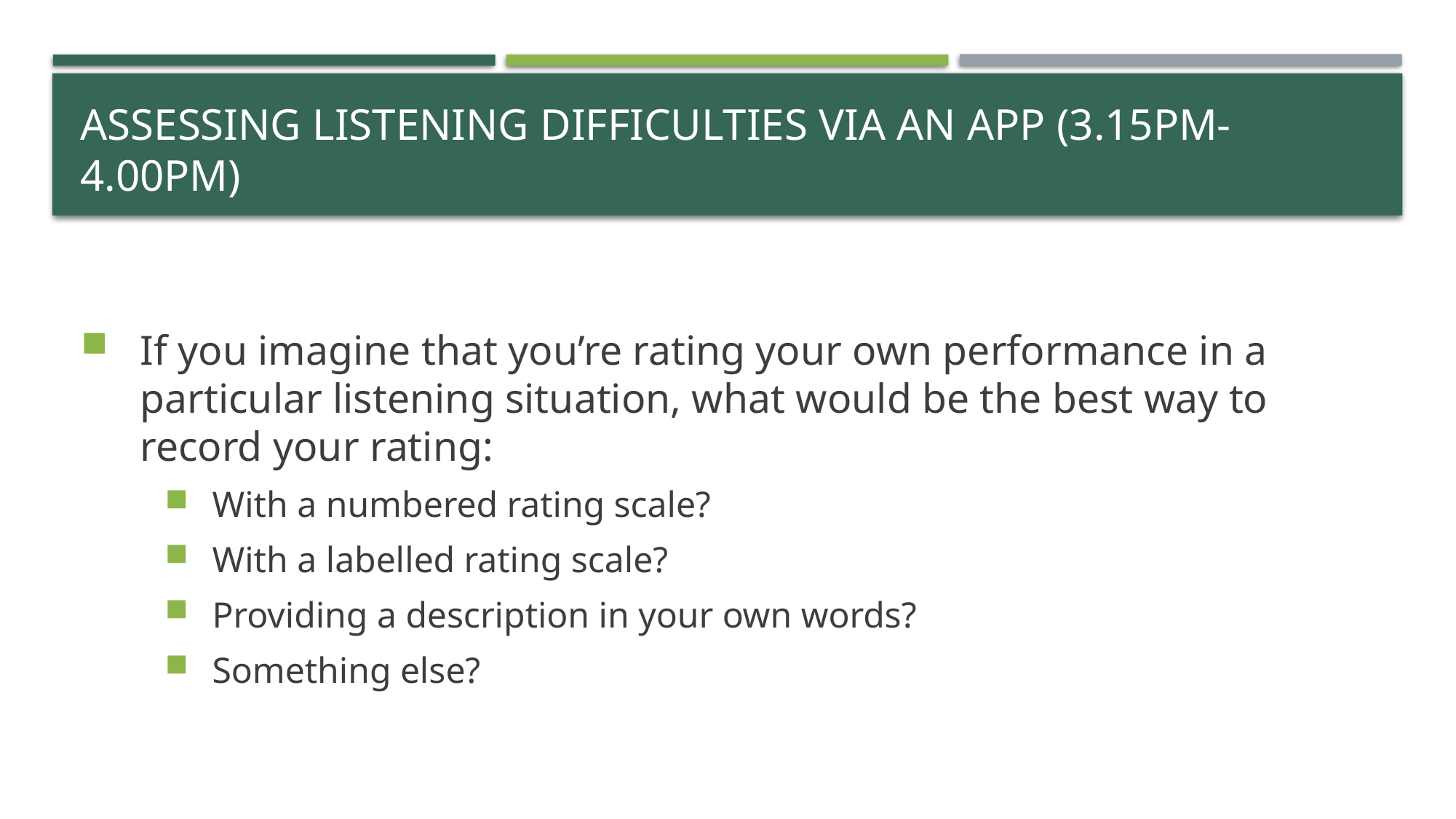

# Assessing listening difficulties via an app (3.15PM-4.00pm)
If you imagine that you’re rating your own performance in a particular listening situation, what would be the best way to record your rating:
With a numbered rating scale?
With a labelled rating scale?
Providing a description in your own words?
Something else?

## Slide 18
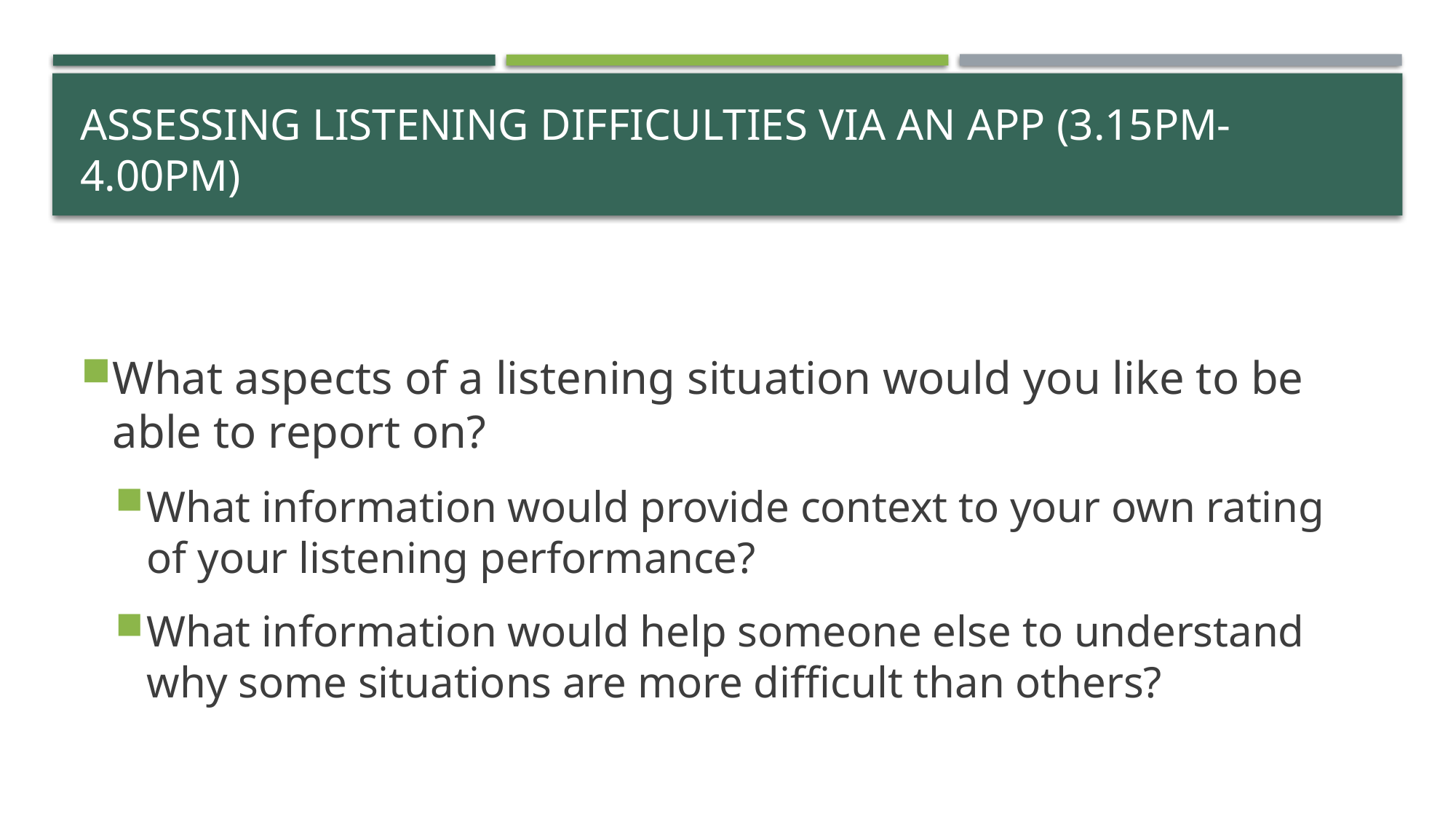

# Assessing listening difficulties via an app (3.15PM-4.00pm)
What aspects of a listening situation would you like to be able to report on?
What information would provide context to your own rating of your listening performance?
What information would help someone else to understand why some situations are more difficult than others?

## Slide 19
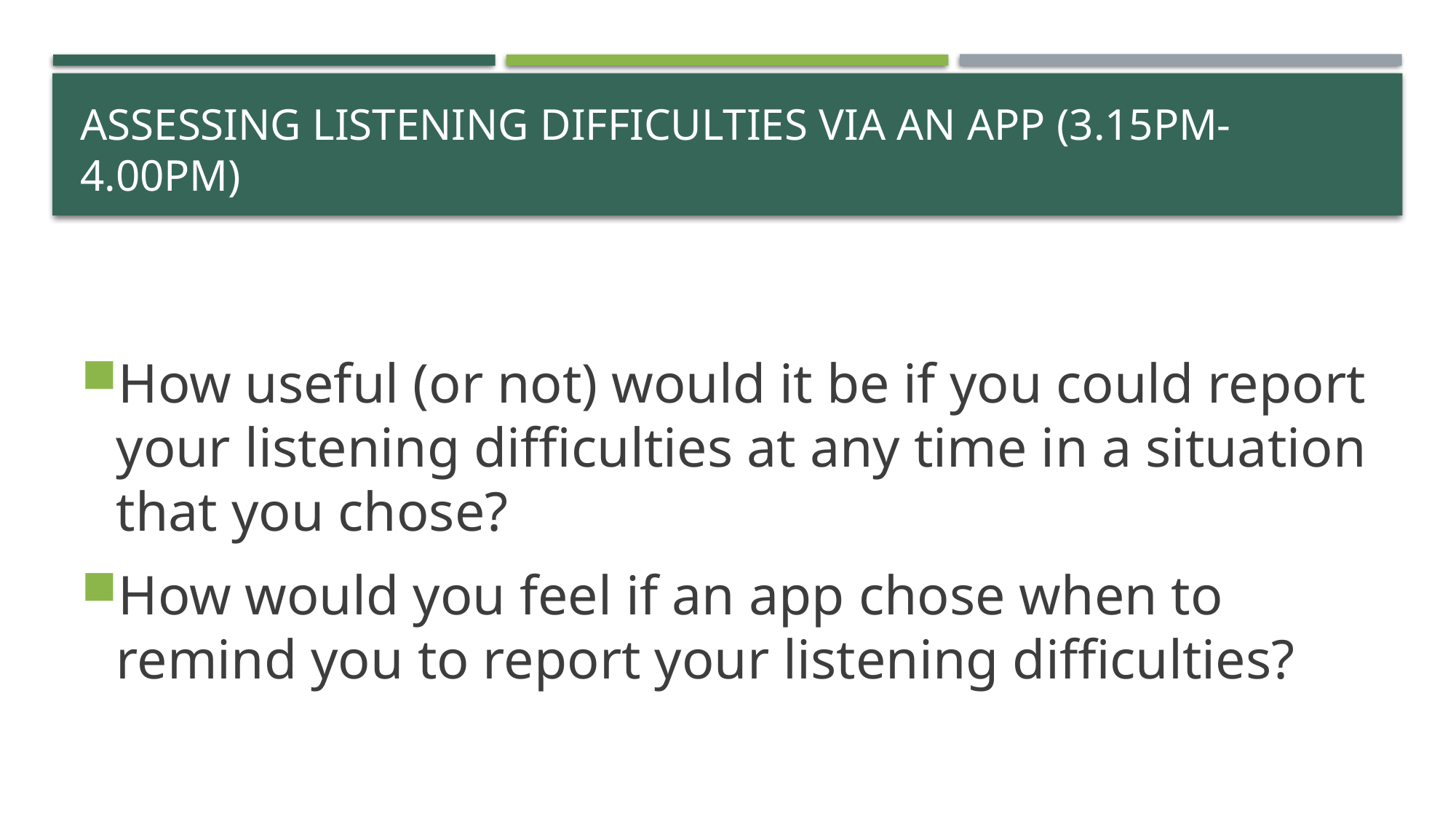

# Assessing listening difficulties via an app (3.15PM-4.00pm)
How useful (or not) would it be if you could report your listening difficulties at any time in a situation that you chose?
How would you feel if an app chose when to remind you to report your listening difficulties?

## Slide 20
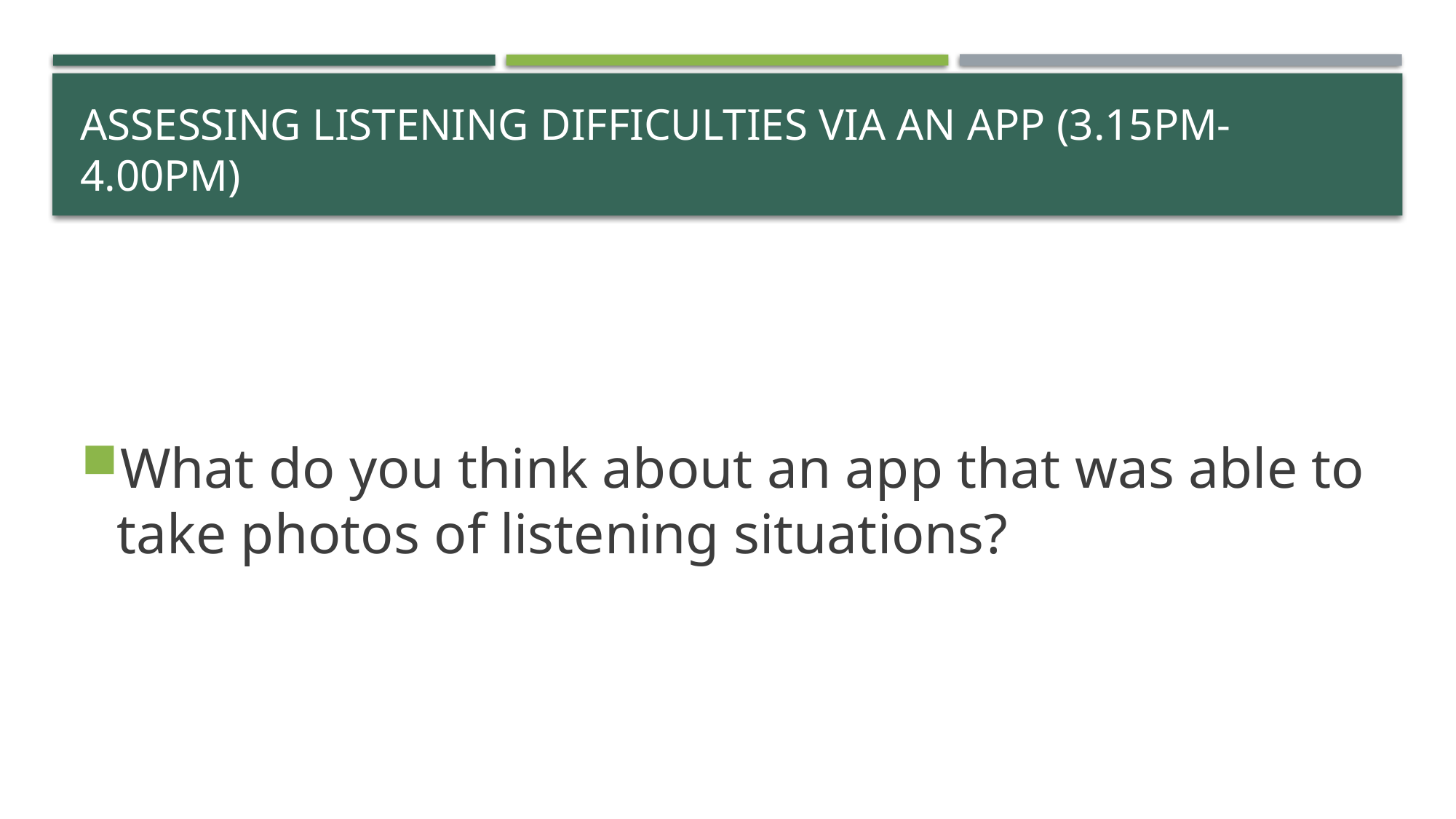

# Assessing listening difficulties via an app (3.15PM-4.00pm)
What do you think about an app that was able to take photos of listening situations?

## Slide 21
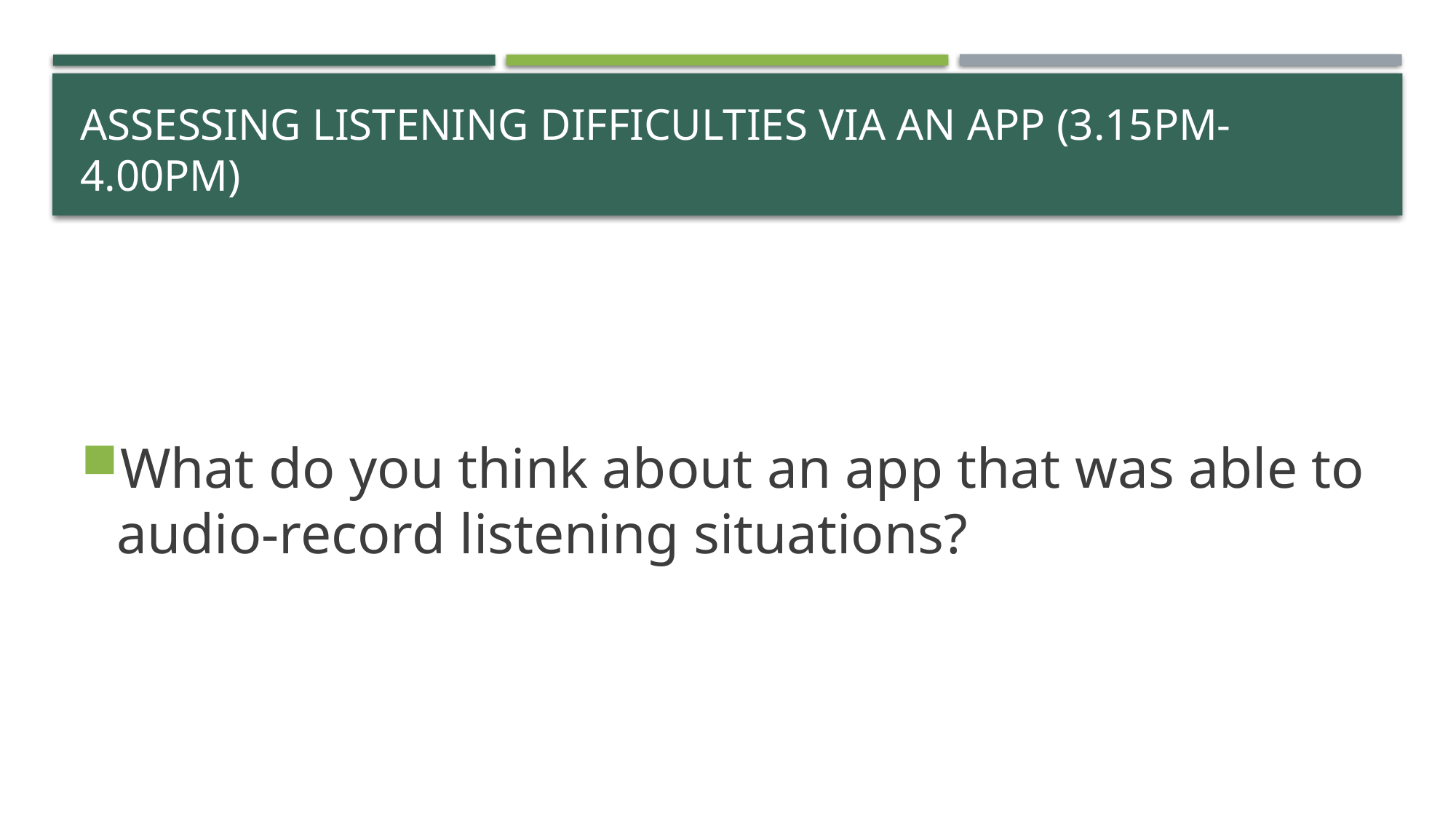

# Assessing listening difficulties via an app (3.15PM-4.00pm)
What do you think about an app that was able to audio-record listening situations?

## Slide 22
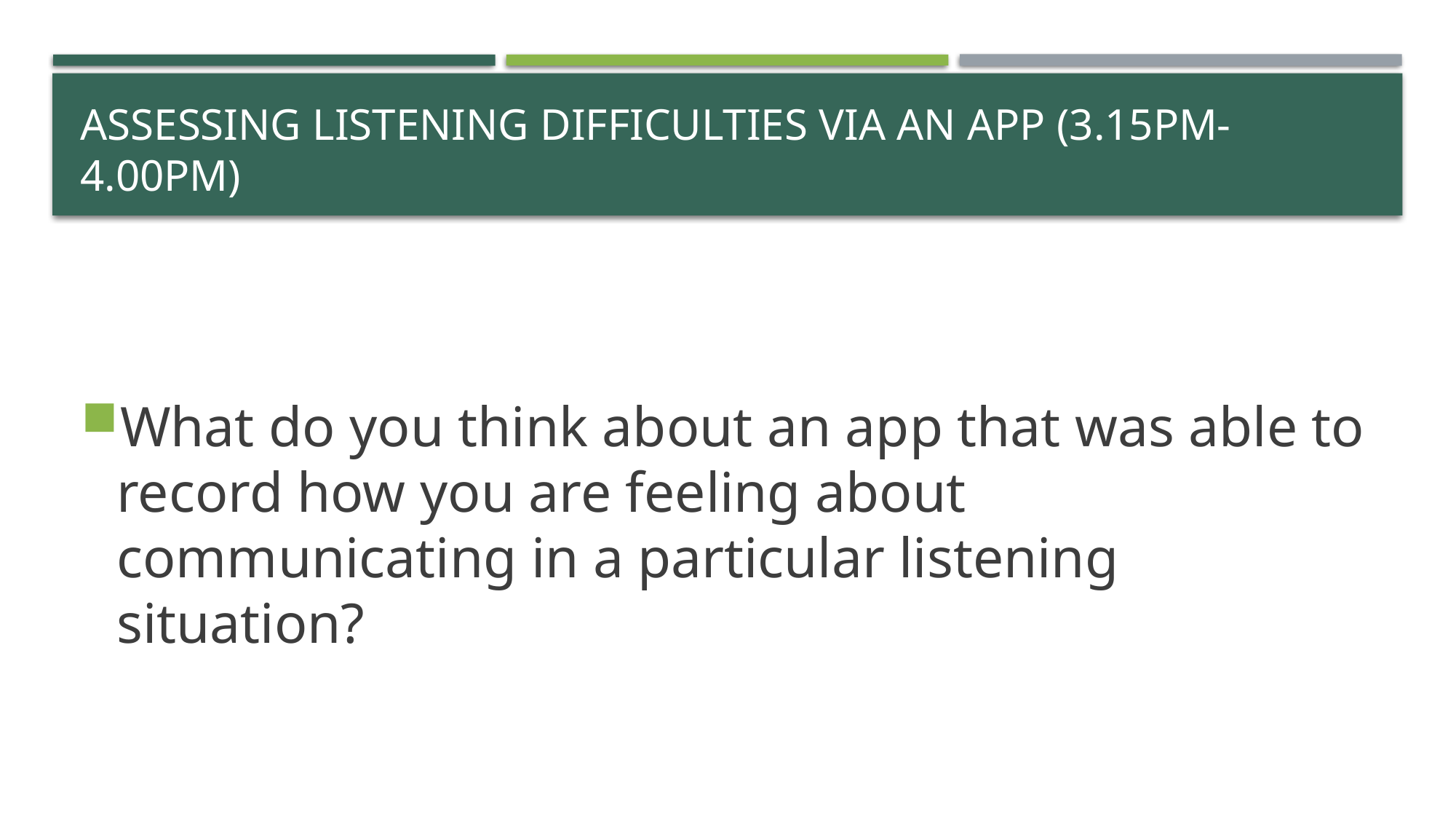

# Assessing listening difficulties via an app (3.15PM-4.00pm)
What do you think about an app that was able to record how you are feeling about communicating in a particular listening situation?

## Slide 23
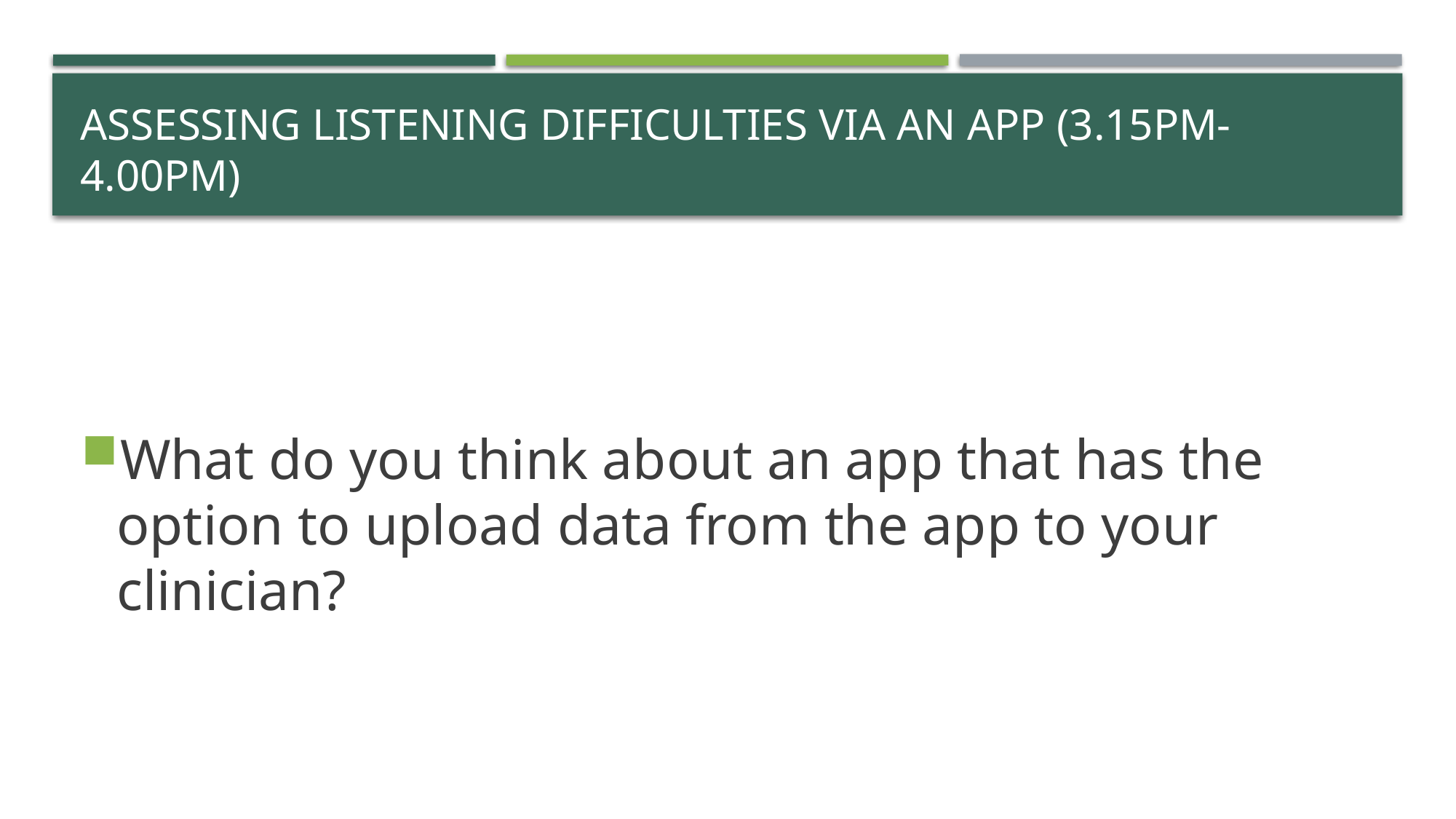

# Assessing listening difficulties via an app (3.15PM-4.00pm)
What do you think about an app that has the option to upload data from the app to your clinician?

## Slide 24
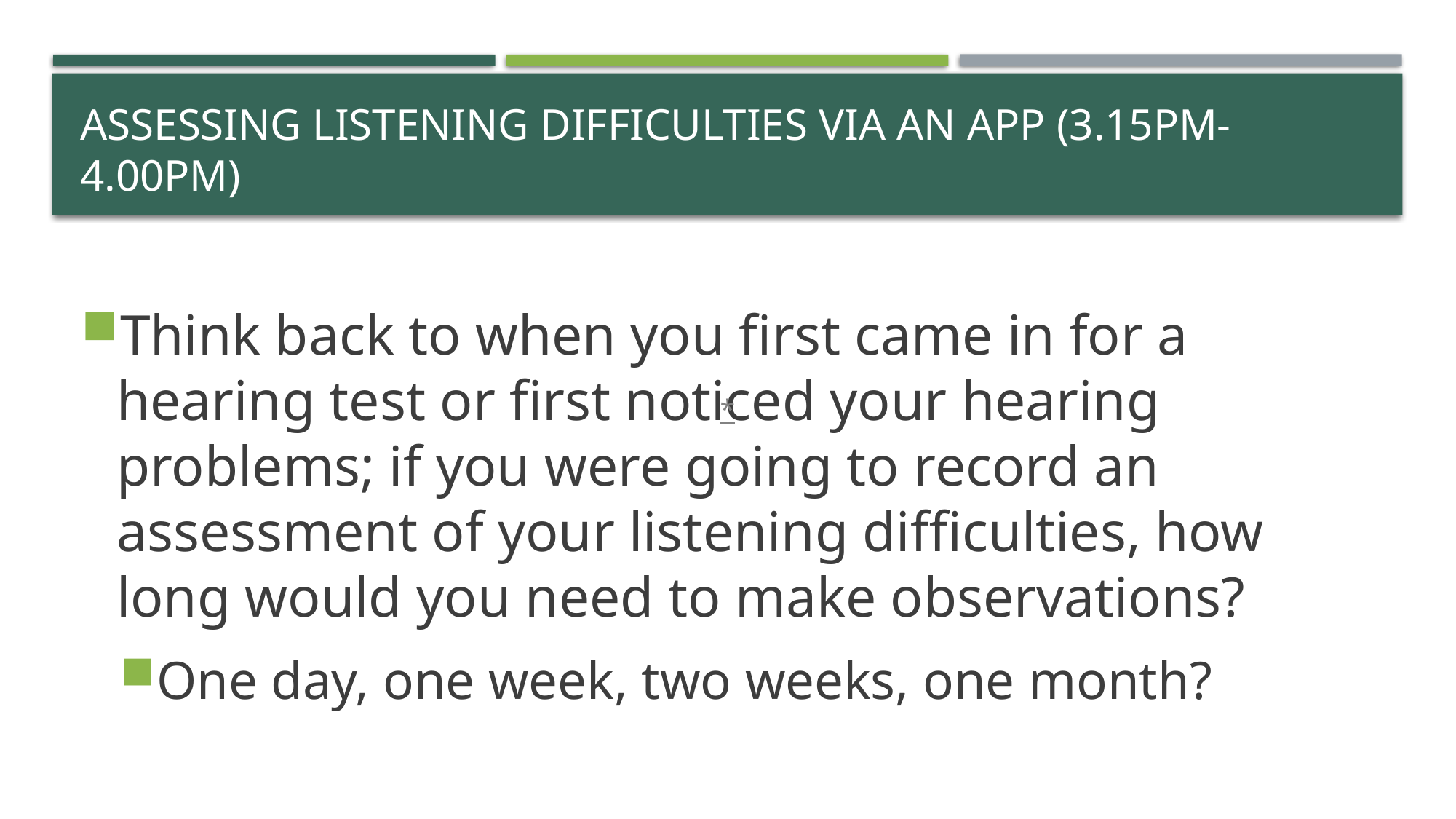

# Assessing listening difficulties via an app (3.15PM-4.00pm)
Think back to when you first came in for a hearing test or first noticed your hearing problems; if you were going to record an assessment of your listening difficulties, how long would you need to make observations?
One day, one week, two weeks, one month?
*

## Slide 25
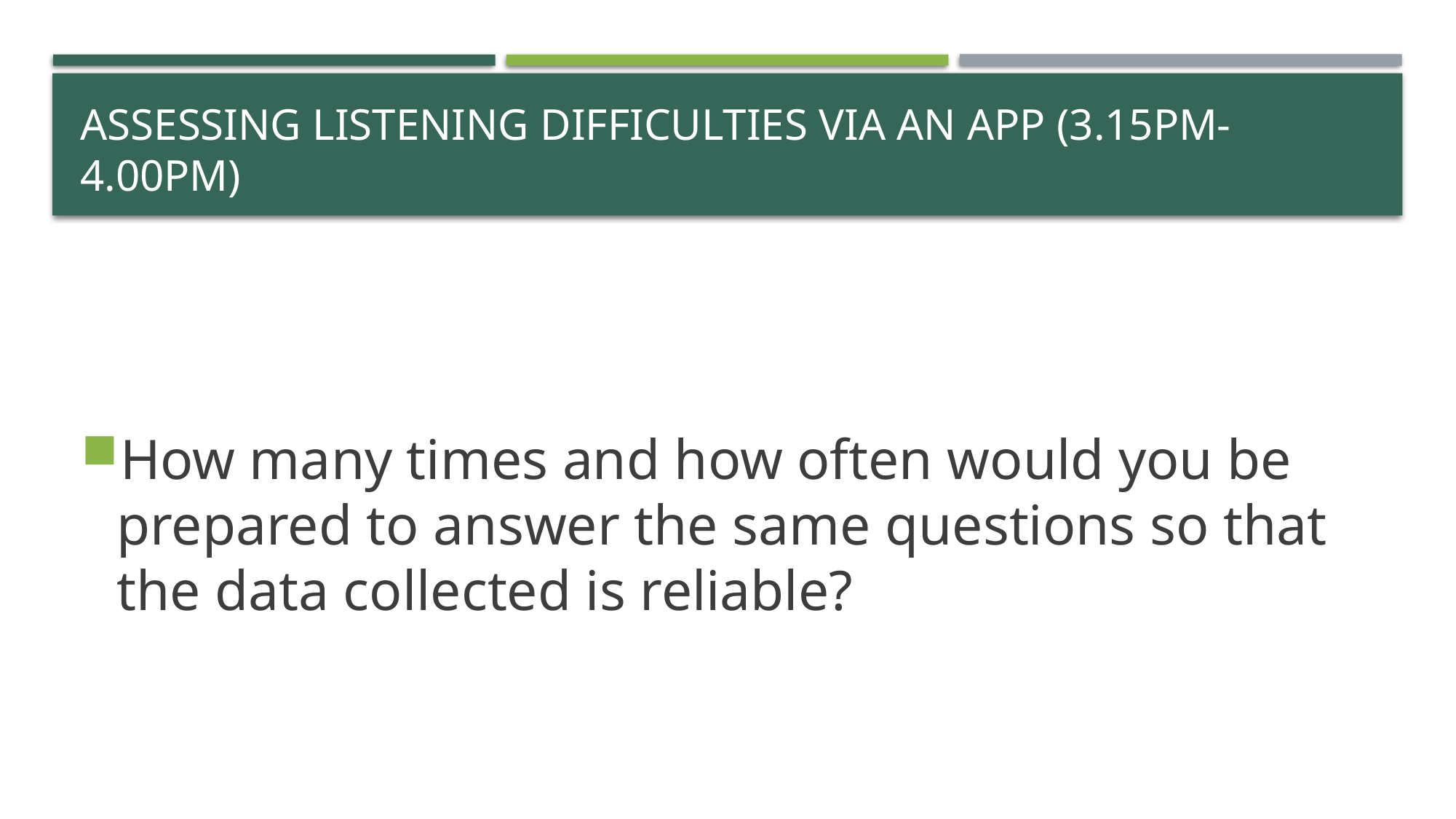

# Assessing listening difficulties via an app (3.15PM-4.00pm)
How many times and how often would you be prepared to answer the same questions so that the data collected is reliable?

## Slide 26
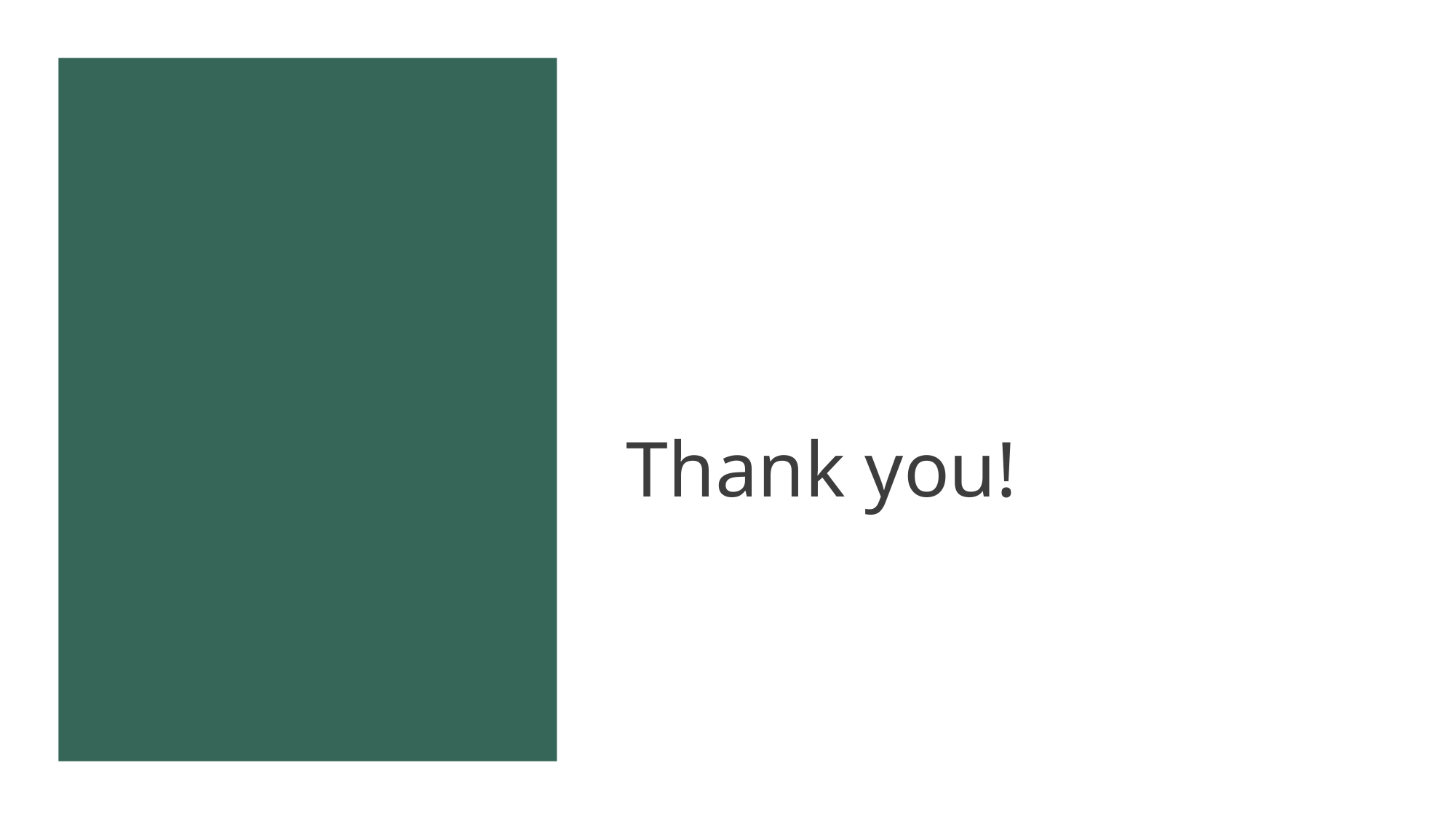

Thank you!
